# Si Doping-Induced Electronic Structure Regulation of Single-Atom Fe Sites for Boosted CO_2_ Electroreduction at Low Overpotentials

**DOI:** 10.34133/research.0079

**Published:** 2023-03-15

**Authors:** Changsheng Cao, Shenghua Zhou, Shouwei Zuo, Huabin Zhang, Bo Chen, Junheng Huang, Xin-Tao Wu, Qiang Xu, Qi-Long Zhu

**Affiliations:** ^1^ State Key Laboratory of Structural Chemistry, Fujian Institute of Research on the Structure of Matter, Chinese Academy of Sciences, Fuzhou, 350002, China.; ^2^ University of Chinese Academy of Science, Beijing, 100049, China.; ^3^ KAUST Catalysis Center (KCC), King Abdullah University of Science and Technology (KAUST), Thuwal, 23955-6900, Saudi Arabia.; ^4^ Department of Chemistry, City University of Hong Kong, Hong Kong, 999077, China.; ^5^ CAS Key Laboratory of Design and Assembly of Functional Nanostructures, Fujian Provincial Key Laboratory of Nanomaterials, FujianInstitute of Research on the Structure of Matter, Chinese Academy of Sciences, Fuzhou, 350002, China.; ^6^ Fujian Science & Technology Innovation Laboratory for Optoelectronic Information of China, Fuzhou, 350108, China.; ^7^ Institute for Integrated Cell-Material Sciences (iCeMS), Kyoto University, Kyoto 606-8501, Japan.; ^8^ Shenzhen Key Laboratory of Micro/Nano-Porous Functional Materials (SKLPM), SUSTech-Kyoto University Advanced Energy Materials Joint Innovation Laboratory (SKAEM-JIL), and Department of Materials Science and Engineering, Southern University of Science and Technology (SUSTech), Shenzhen, 518055, China.

## Abstract

Transition metal-based single-atom catalysts (TM-SACs) are promising alternatives to Au- and Ag-based electrocatalysts for CO production through CO_2_ reduction reaction. However, developing TM-SACs with high activity and selectivity at low overpotentials is challenging. Herein, a novel Fe-based SAC with Si doping (Fe-N-C-Si) was prepared, which shows a record-high electrocatalytic performance toward the CO_2_-to-CO conversion with exceptional current density (>350.0 mA cm^−2^) and ~100% Faradaic efficiency (FE) at the overpotential of <400 mV, far superior to the reported Fe-based SACs. Further assembling Fe-N-C-Si as the cathode in a rechargeable Zn-CO_2_ battery delivers an outstanding performance with a maximal power density of 2.44 mW cm^−2^ at an output voltage of 0.30 V, as well as high cycling stability and FE (>90%) for CO production. Experimental combined with theoretical analysis unraveled that the nearby Si dopants in the form of Si-C/N bonds modulate the electronic structure of the atomic Fe sites in Fe-N-C-Si to markedly accelerate the key pathway involving *CO intermediate desorption, inhibiting the poisoning of the Fe sites under high CO coverage and thus boosting the CO_2_RR performance. This work provides an efficient strategy to tune the adsorption/desorption behaviors of intermediates on single-atom sites to improve their electrocatalytic performance.

## Introduction

Electrocatalytic CO_2_ reduction reaction (CO_2_RR) powered by renewable energy is a promising strategy to reduce anthropogenic CO_2_ emission while producing valuable chemicals and fuels [1–3]. Among the various CO_2_ reduction products including CO, formic acid, methane, ethylene, and ethanol, CO is the most common one and is a vital feedstock for chemical and industrial (e.g., Fischer–Tropsch process) applications [4,5]. More notably, after considering the market size and product price, as well as capital and operating costs, the electrochemical conversion of CO_2_ to CO has proven to be the most economically and technologically feasible way to realize industrial applications in the current stage [4,6]. However, one of the biggest challenges in turning this vision into reality is the development of cost-effective yet high-performance electrocatalysts that can efficiently and selectively convert CO_2_ into CO, especially at low overpotentials.

Noble metal-based materials including Au, Ag, and Pd are recognized as the most efficient CO_2_RR electrocatalysts for CO production at low overpotentials [7–10]. However, the high cost and scarcity limit their further large-scale applications. Recently, as alternatives, earth-abundant transition metal-based single-atom catalysts (SACs) with atomically anchored metal atoms in the form of M-N_4_ on N-doped carbon substrates (also known as M-N-C) have attracted great interest for CO_2_RR due to their maximized atomic utilization efficiency, tunable electronic properties, and distinctive catalytic characteristics [11–15]. Among them, the Fe-N-C catalysts are expected to exhibit high CO_2_RR performance at lower overpotentials, which can be comparable to or even better than noble metal-based electrocatalysts [16–21]. For example, Ye et al. [21] prepared a Fe-N-C catalyst with highly exposed Fe-N sites (C-AFCZIF-8), which displayed high Faradaic efficiency (FE) for CO generation (FE_CO_) at low overpotentials (e.g., 89.1% at −0.33 V), outperforming the nanostructured Pd catalyst. In another work, the Fe^3+^-N-C catalyst reported by Hu et al. exhibited an ultralow onset overpotential of 80 mV for CO generation. More notably, by using a flow cell reactor, the partial current density of CO (*j_CO_*) even can reach 94 mA cm^−2^, without sacrificing the values of FE_CO_ at an overpotential of only 340 mV [16].

Behind the thriving development of Fe-N-C-based CO_2_RR electrocatalysts, 2 main issues deserve attention: (a) despite high FE_CO_ (>90%), low *j_CO_* at low overpotentials; and (b) susceptible to poisoning of the Fe-N_4_ sites from high *CO intermediate coverage at high *j_CO_* [22–25]. According to previous works, heteroatom doping in carbon substrates is feasible to rationally regulate the electronic structure and local environment of metal centers in SACs, which can not only enhance their intrinsic activity, but also optimize the adsorption/desorption behavior of key intermediates, thereby further improving the electrocatalytic performance [26–31]. As a proof of concept, introducing P atoms in the Fe-N-C catalyst (Fe-SAC/NPC) would improve the stabilization of the key *COOH intermediate on Fe sites, thus boosting the CO_2_RR performance [27]. Recently, Liu and coworkers synthesized a Fe-based SAC with B and N co-doped carbon as the substrate (Fe-SA/BNC), which showed a high FE_CO_ of ~94% at −0.7 V in the H-type cell, as well as the high current density (~130 mA cm^−2^) and FE_CO_ (~99%) by using the membrane electrode assembly. Density functional theory (DFT) analysis revealed that the incorporation of B atoms modulates the electronic structure of the Fe sites, thereby enhancing the CO_2_RR performance [30], although it is effective to improve the CO_2_RR performance over Fe-N-C catalysts by heteroatom doping in carbon substrates. However, to the best of our knowledge, there are currently few Fe-N-C catalysts capable of simultaneously achieving high FE_CO_ (>90%) and *j_CO_* (>200 mA cm^−2^), which is a prerequisite for industrial applications [4].

In this work, a DFT-instructed Si-doped Fe-N-C electrocatalyst (Fe-N-C-Si) was developed for CO_2_RR for the first time. Both experimental characterizations, including x-ray absorption spectroscopy (XAS), x-ray photoelectron spectroscopy (XPS), and in situ attenuated total reflection-infrared (ATR-IR) spectra, and theoretical analysis unraveled that the incorporation of Si atoms can regulate the electronic structure of the atomic Fe centers in Fe-N-C-Si, thereby weakening the adsorption of the *CO intermediate and preventing site poisoning. As feedback, the resultant Fe-N-C-Si exhibited an unparalleled CO_2_RR performance with an industry-compatible high *j_CO_* (>230.0 mA cm^−2^) and FE_CO_ (>95%) at an ultralow overpotential of only 300 mV, much superior to the state-of-the-art Fe-N-C-based and noble-metal-based electrocatalysts reported to date. Additionally, an aqueous rechargeable Zn-CO_2_ battery (ZCB) assembled with Fe-N-C-Si as the cathode is capable of delivering a maximal power density of 2.44 mW cm^−2^ at 0.30 V with a current density of 8.2 mA cm^−2^, as well as the high FE_CO_ (>90%) and stability, further demonstrating the feasibility of its practical implementation.

## Results

### Theoretical study of Si-doping effect on the CO_2_RR performance of Fe-N-C

**Fig. 1.**
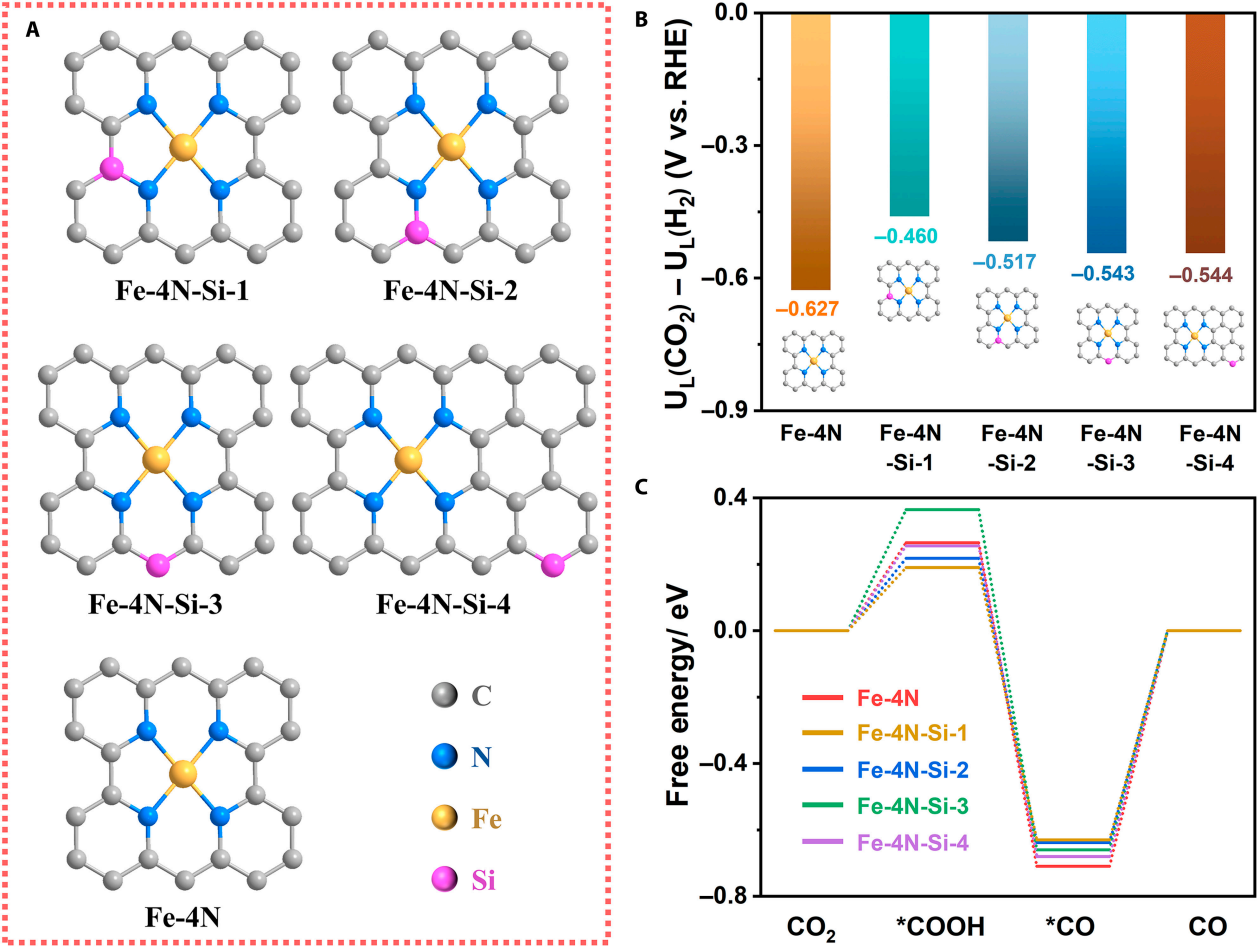
Theoretical study of the Si-doping effect of the CO_2_RR performance of Fe-N-C. (A) Scheme of the structural configurations for DFT calculation. (B) The calculated values of U_L_(CO_2_) − U_L_(H_2_) on the different single-atom Fe configurations. (C) Free energy diagrams of CO_2_RR.

According to previous works, doping heteroatoms (including B, O, F, P, and S) into the carbon substrates of carbon-based SACs will inevitably introduce additional defects due to the different coordination numbers of C and these heteroatoms [26,30,32,33], making it difficult to exactly evaluate the effect of those doped heteroatoms on CO_2_RR performance in the presence of additional defects. Since a Si atom has the same coordination structure as a C atom, substituting part of C atoms with Si atoms will not introduce additional defects, which provides an ideal model for exploring the effect of heteroatom doping on the CO_2_RR performance of SACs, especially Si-doped Fe-N-C catalysts. Accordingly, 4 possible configurations (abbreviated as Fe-4N-Si-1, Fe-4N-Si-2, Fe-4N-Si-3, and Fe-4N-Si-4) were constructed (Fig. 1A), and the possible reaction pathways and corresponding energies during the CO_2_RR process were calculated through DFT. Meanwhile, the conventional Fe-N_4_ configuration (abbreviated as Fe-4N) served as a control. Considering that hydrogen evolution reaction (HER) is the main competitive reaction against CO_2_RR, the corresponding limiting potential differences between CO_2_RR and HER (U_L_(CO_2_) − U_L_(H_2_)) were first calculated to quantify the CO_2_RR selectivity. As displayed in Fig. 1B, 4 configurations with Si doping, especially Fe-4N-Si-1, have more positive U_L_(CO_2_) − U_L_(H_2_) values than that of Fe-4N, demonstrating the higher selectivity for CO_2_RR. Moreover, it is well known that the electroreduction of CO_2_ to CO is a 2-electron process through the formation of *COOH and *CO intermediates [2]. Figure 1C shows the free-energy diagram of the pathways for CO generation. Notably, the formation of the *COOH intermediate via the first electron transfer process and the desorption of the *CO intermediate on the atomic Fe sites are endothermic for all configurations. The higher free energy of the later step means that the desorption of the *CO intermediate determines the total reaction rate, while the former step is the potential-determining step, which is consistent with previous works [31,34]. Notably, the free energy of *CO intermediate desorption on the Fe sites significantly decreases from |−0.71| V in Fe-4N to |−0.64| V in Fe-4N-Si-1, indicating that the incorporation of the neighboring Si atoms promotes the desorption of the *CO intermediate on the Fe-N_4_ moieties, thereby promising to improve CO_2_RR performance, especially at high *CO intermediate coverage.

### Structural and morphology characterizations of catalysts

**Fig. 2.**
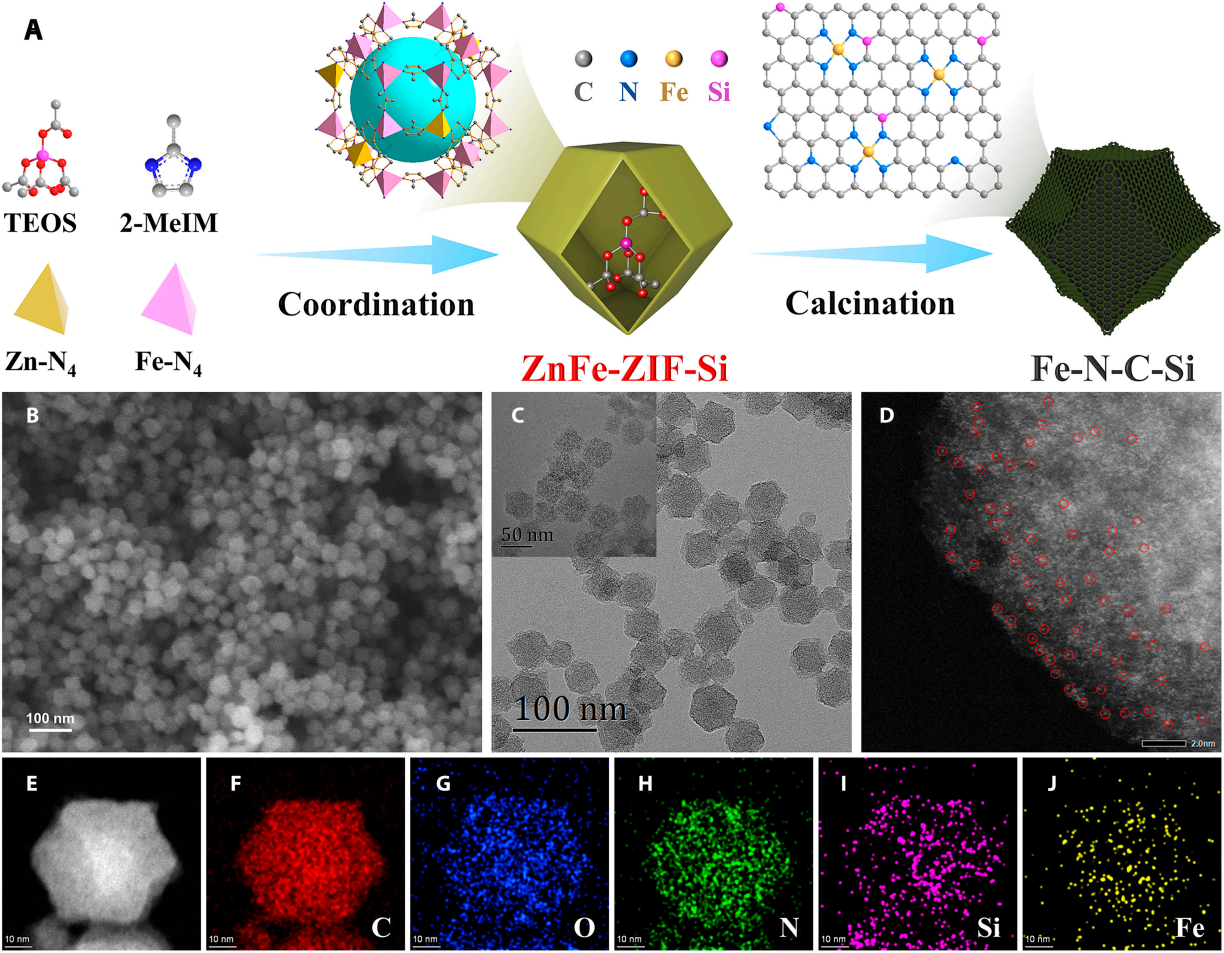
Structural and morphology characterizations. (A) Schematic illustration of the synthesis procedure for Fe-N-C-Si. (B) SEM, (C) TEM, (D) HAADF-STEM, and (E to J) EDX elemental mapping images of Fe-N-C-Si.

Motivated by the theoretical analysis, a Si-doped Fe-N-C electrocatalyst (Fe-N-C-Si) was prepared correspondingly through a 2-step process (Fig. 2A). Specifically, the Fe-doped metal–organic framework (MOF) precursor (ZnFe-ZIF-Si) encapsulated with a Si source (i.e., tetraethyl orthosilicate [TEOS]) was first prepared through the assembly of Zn^2+^ and Fe^3+^ with 2-methylimidazole (2-MeIM). Similarly, the Fe-doped MOF precursor (ZnFe-ZIF) without a Si source was also prepared for comparison. Powder x-ray diffraction (PXRD) patterns (Fig. S1) and scanning electron microscopy (SEM) images (Fig. S2) indicate the successful growth of rhombic dodecahedral ZnFe-ZIF-Si with an average size of about 60 nm. The Brunauer–Emmett–Teller (BET) area and micropore content of ZnFe-ZIF-Si are significantly reduced compared with ZnFe-ZIF, which implies the successful encapsulation of TEOS molecules in the MOF pores (Fig. S3). Afterward, Fe-N-C-Si was prepared through a high-temperature pyrolysis process. SEM and transmission electron microscopy (TEM) images show that Fe-N-C-Si well inherits the rhombic dodecahedral morphology of the MOF precursor, except for some shrinkage (Fig. 2B and C and Fig. S4). Moreover, as shown in Fig. 2D, many bright spots in the atomic range, rather than any Fe nanoparticles or clusters, were observed in the high-angle annular dark-field scanning TEM (HAADF-STEM) image, reflecting the atomic dispersion of Fe atoms in Fe-N-C-Si. Meanwhile, energy-dispersive x-ray (EDX) elemental mappings show that C, O, N, Si, and Fe elements are uniformly distributed throughout the polyhedra of Fe-N-C-Si (Fig. 2E to J). Similarly, SEM, TEM, and HAADF-STEM images also confirm the atomic dispersion of Fe atoms in shrunken rhombic dodecahedral Fe-N-C (Fig. S5). Besides, only 2 broad peaks centered at 2θ ≈ 24° and 43° are observed in the PXRD patterns of Fe-N-C-Si and Fe-N-C, which belong to the (002) and (101) planes of graphitized carbon, respectively (Fig. S6), further indicating that there is no metallic Fe species. Based on inductively coupled plasma optical emission spectrometry (ICP-OES) analysis, the Fe content in Fe-N-C-Si was assessed to be about 1.24 wt%, which is about 3 times higher than that of Fe-N-C (0.42 wt%). Raman spectra of Fe-N-C-Si and Fe-N-C show the similar D- and G-band intensity ratios (*I*_D_/*I*_G_), suggesting the similar degree of disorder in these samples (Fig. S7A) [35], which is further evidenced by their similar peak intensities in solid electron paramagnetic resonance (EPR) spectra (Fig. S7B), proving that Si doping will not introduce additional defects. Additionally, N_2_ adsorption–desorption isotherms imply that Fe-N-C-Si possesses much larger BET-specific surface area (1,172.4 m^2^/g) and pore volume (2.014 m^3^/g^−1^) than Fe-N-C (635.0 m^2^/g and 0.819 m^3^/g^−1^) (Fig. S8 and Table S1), which is beneficial to expose more active sites and accelerate mass transfer, thereby improving the electrocatalytic performance [36].

**Fig. 3.**
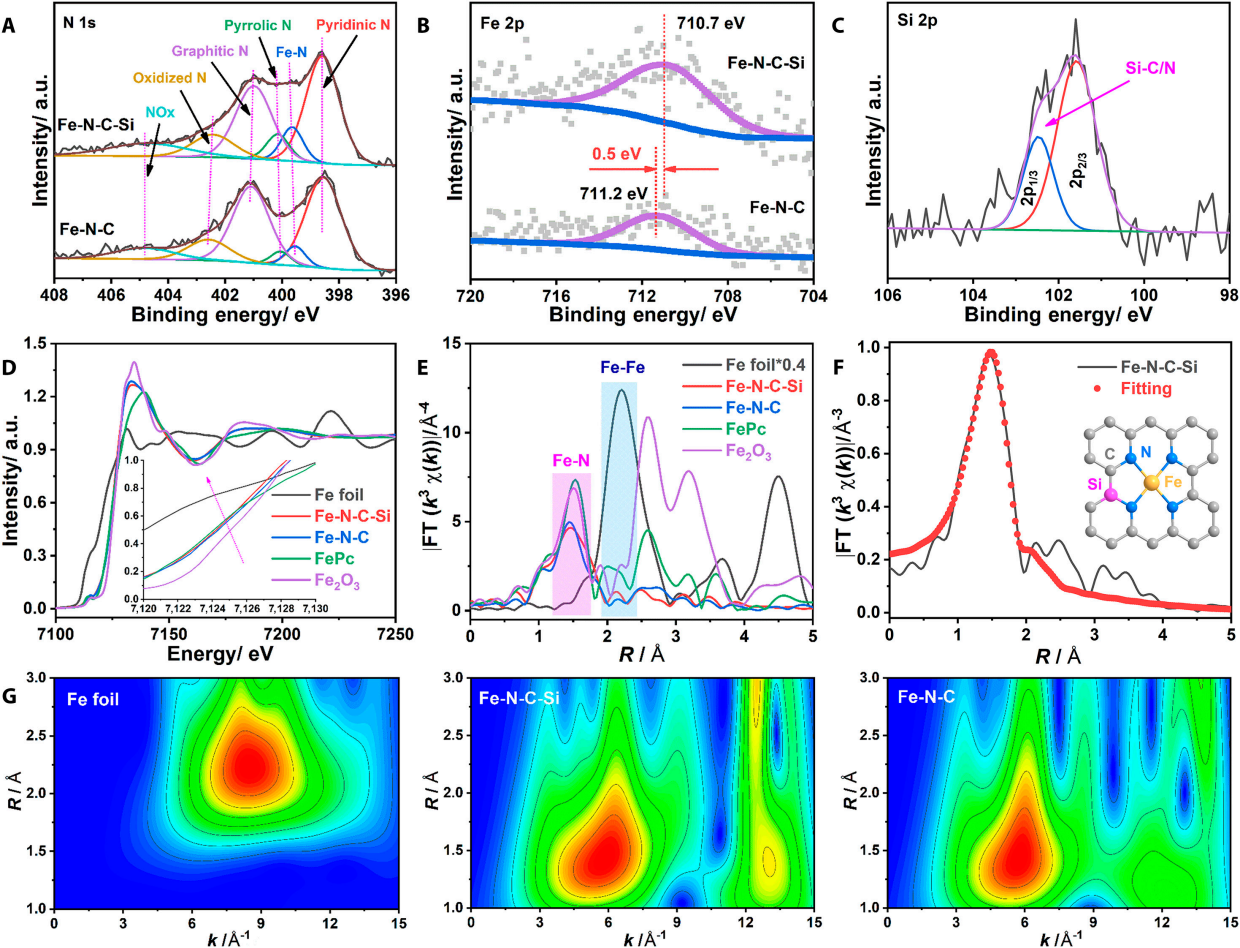
Structure characterizations. (A) N 1s and (B) Fe 2p XPS spectra of Fe-N-C and Fe-N-C-Si. (C) Si 2p XPS spectrum of Fe-N-C-Si. (D) Normalized Fe K-edge XANES and (E) Fourier-transformed EXAFS spectra of Fe-N-C-Si, Fe-N-C, and references. (F) Wavelet transform EXAFS plots of Fe foil, Fe-N-C-Si, and Fe-N-C. (G) The fitting EXAFS spectrum of Fe-N-C-Si and the fitting model (inset).

The surface chemical compositions and electronic states of Fe-N-C-Si and Fe-N-C were firstly explored by XPS spectroscopy. Survey spectra show the existence of C, O, and N in both samples (Fig. S9), and the N contents in Fe-N-C-Si and Fe-N-C are 5.56 and 4.69 at%, respectively. However, the signals of both Fe (0.29 and 0.15 at% for Fe-N-C-Si and Fe-N-C, respectively) and Si (0.39 at% in Fe-N-C-Si) are very weak due to their low contents. In the high-resolution N 1s spectra (Fig. 3A and Fig. S10), the peaks at around 398.9, 399.1, 400.1, 401.0, 403.0, and 405.1 eV are ascribed to pyridinic-N, Fe-N, pyrrolic-N, graphitic-N, oxidized N, and NO_x_, respectively [19,37]. Regarding Fe 2p spectra (Fig. 3B), the peak at around 711 eV is attributed to Fe 2p_3/2_ electronic configurations with an oxidation state close to +2, manifesting the partial oxidation of Fe atoms [34,38]. Compared with Fe-N-C, the Fe 2p spectrum of Fe-N-C-Si slightly shifts toward the lower binding energy, indicating that partial electrons are transferred from Si atoms to central Fe atoms through N atoms [28]. Such electron transfer could lead to a negative shift of the d-band center of Fe atoms relative to the Fermi level, which may enhance the electronic localization and thereby weaken the adsorption of the *CO intermediate on atomic Fe sites [39,40]. Additionally, the Si 2p spectrum of Fe-N-C-Si can be deconvoluted into 2 peaks located around 101.9 and 102.5 eV (Fig. 3C), which are related to the presence of C-Si and N-Si bonds, respectively [41,42]. Meanwhile, the absence of characteristic peaks associated with the Fe-Si bond (~99 to 100 eV) further implies that the doped Si atoms are bonded to the surrounding C/N atoms, rather than directly to the central Fe atoms [43]. Furthermore, the similar signal intensities of Si 2p spectra with different Ar etch times firmly imply the uniform doping of Si atoms throughout Fe-N-C-Si (Fig. S11). Although the exact positions of Si doped on the catalyst cannot be well confirmed, the aforementioned theoretical calculation results indicate that the Si dopants at the possible locations are beneficial to improve the CO_2_RR performance of Fe-N-C-Si.

To further identify the local electronic and geometrical structures of the Fe centers in Fe-N-C-Si and Fe-N-C, XAS spectra were collected and analyzed. As shown in the Fe K-edge x-ray absorption near-edge structure (XANES) spectra (Fig. 3D), the absorption energy edges of Fe-N-C-Si and Fe-N-C are presented between those of Fe foil and Fe_2_O_3_ and close to that of FePc, implying that the oxidation state of the atomic Fe centers should be around +2 [44]. Meanwhile, it can also be observed that the pre-edge absorption energy of Fe-N-C-Si is slightly lower than that of Fe-N-C, suggesting that the incorporation of Si induces the electron enrichment on Fe sites [26], which is in line with the XPS results. Moreover, as shown in Fig. 3E, the Fourier-transformed extended x-ray absorption fine structure (FT-EXAFS) spectra show that Fe-N-C-Si, Fe-N-C, and FePc display similar peaks at ~1.45 Å, and have no peak at ~2.2 Å, proving the atomic dispersion of Fe atoms in Fe-N-C-Si and Fe-N-C. Besides, for Fe-N-C-Si, no peak corresponding to Fe-Si bonds (>2 Å) is observed in the FE-EXAFS spectra [45–47], manifesting that Si is bonded to C/N atoms, rather than Fe atoms in Fe-N-C-Si, consistent with the XPS results. In addition, the wavelet transform EXAFS contour plot of the Fe foil exhibits an intensity maximum at about 8.5 Å^−1^ (Fig. 3F), which is attributed to the Fe-Fe signal. However, Fe-N-C-Si and Fe-N-C only present a similar intensity maximum that can be assigned to the Fe-N path at around 4.7 Å^−1^ (Fig. 3F), further endorsing the atomic dispersion of Fe atoms [48]. According to the optimized DFT calculation models (Fig. 1A) and the fitting of EXAFS spectra (Fig. 3G, Figs. S12 to S14, and Table S2), it suggests that the Fe centers are 4-coordinated in the form of Fe-N_4_ configuration with the Si doping in the second shell for Fe-N-C-Si.

### Electrocatalytic CO_2_RR performances of catalysts

**Fig. 4.**
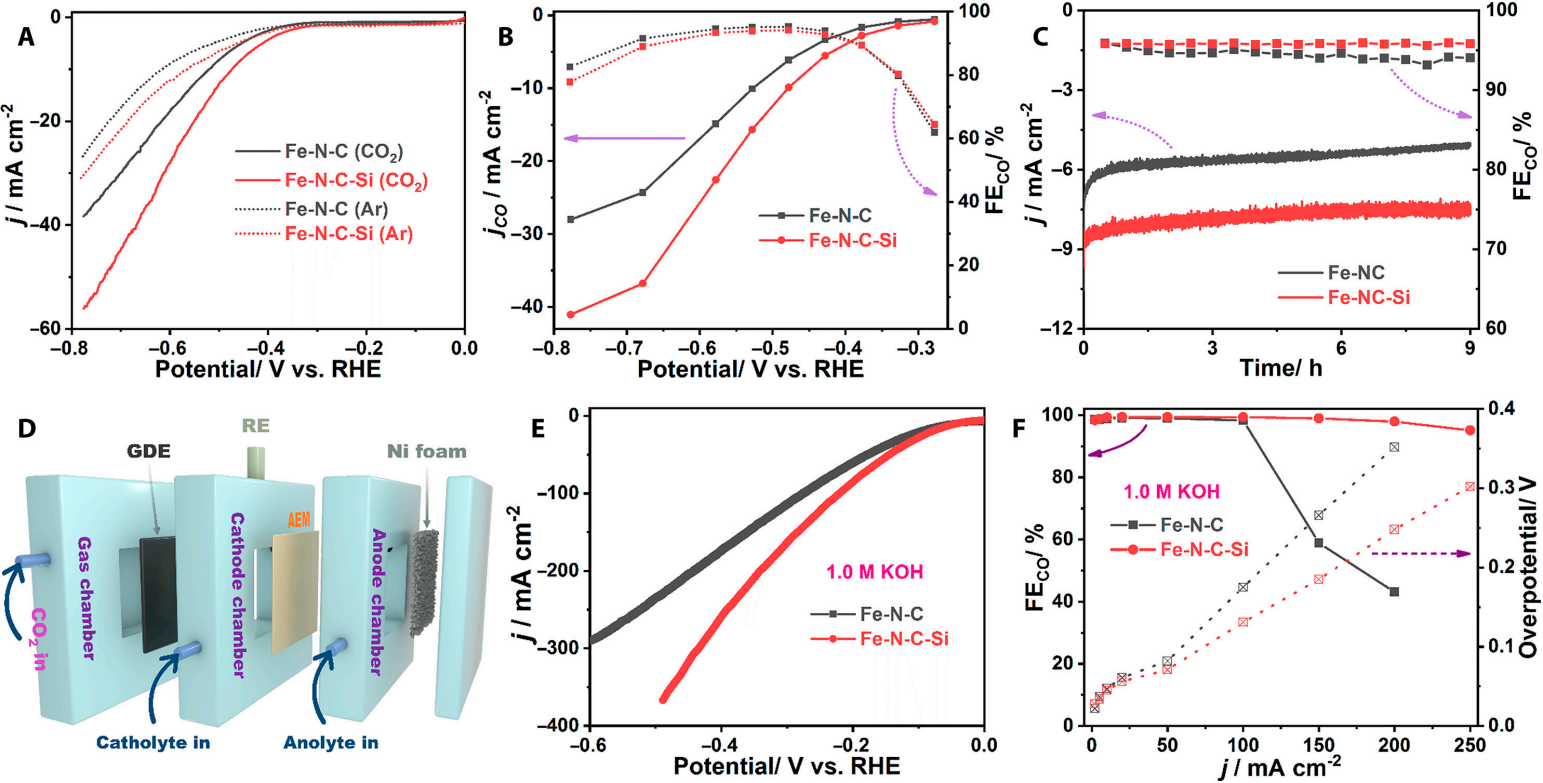
Electrocatalytic CO_2_RR measurements. (A) LSV curves collected in Ar- or CO_2_-saturated 0.5 m KHCO_3_. (B) Potential-dependent FE_CO_ and *j_CO_*. (C) Long-term stability test at −0.48 V. (D) Schematic illustration of the self-designed flow cell. (E) LSV curves and (F) FEs and corresponding overpotentials at different current densities for CO generation by using 1.0 m KOH as the electrolyte.

The electrocatalytic CO_2_RR performance over Fe-N-C-Si and Fe-N-C was first evaluated in a typical H-type cell. As shown in the polarization curves (Fig. 4A), both of them delivered much larger current densities under a CO_2_ atmosphere than those recorded under an Ar atmosphere, manifesting their CO_2_RR activities. Meanwhile, the calculated CO_2_RR efficiency of Fe-N-C-Si is higher than that of Fe-N-C (Fig. S15), which preliminarily confirms its better CO_2_RR performance. Moreover, the products during the CO_2_RR process were quantitatively analyzed, with H_2_ and CO as the only detectable products. For Fe-N-C-Si, as shown in Fig. 4B, CO can be reliably detected at −0.28 V (vs. RHE, the same below), corresponding to an ultralow overpotential of only 170 mV, which is one of the lowest overpotentials for CO generation reported to date (Table S3). Afterward, with the decrease of the applied potentials, FE_CO_ quickly increases to the maximum of 95.22% at −0.48 V, and can be kept >80% in a large potential window from −0.33 to −0.78 V. The decrease of FE_CO_ at more negative applied potentials should be caused by the enhanced competing hydrogen evolution reaction (HER), which may originate from the combination of the difficulty of *CO intermediate desorption on Fe sites at high current densities and the limitation of solubility and mass transfer of CO_2_ in aqueous solution [49]. For Fe-N-C, despite having the same onset potential, and the similar FE_CO_ at the same applied potentials, the values of *j_CO_* are much lower compared to Fe-N-C-Si (Fig. 4B). In particular, Fe-N-C-Si can deliver a *j_CO_* of about −41.05 mA cm^−2^ at −0.78 V, which is about 1.46 times higher than that of Fe-N-C (28.03 mA cm^−2^), indicating its much superior CO_2_RR activity. Further characterizations showed that Fe-N-C-Si possesses a higher CO_2_ adsorption ability (Fig. S16) and smaller Tafel slopes and charge transfer resistance in comparison with Fe-N-C (Fig. S17A and B), which should be helpful to improving the CO_2_RR activity. Moreover, it is worth noting that Fe-N-C-Si afforded the much higher *j_CO_* normalized by electrochemically active surface area, manifesting that Si doping is capable of promoting the intrinsic activity of the single-atom Fe sites (Figs. S17C and D and S18). Besides, the long-term electrolysis stability of Fe-N-C-Si and Fe-N-C was further measured. As shown in Fig. 4C, the gradual decrease of the total current density for both samples may be caused by the consumption of CO_2_ near the catalyst at the initial stage and the gradual weakening of the cathode hydrophobicity during the long-term electrolysis [50]. However, the almost unchanged FE_CO_ values, compositions, morphology, and structure (Figs. S19 to S24) demonstrate the satisfactory durability of Fe-N-C and Fe-N-C-Si during long-term electrolysis, which further suggests that Si doping has a slight effect on the stability of Fe-N-C electrocatalysts at low current densities (i.e., low CO coverage) during the CO_2_RR process. In addition, control experiments revealed that the atomic Fe centers should be the actual active sites (Figs. S25 and S26), and the content of the doped Si has little effect on the structure, morphology, and CO_2_RR performance of the Fe-N-C-Si catalysts (Figs. S27 to S30 and Table S1). Consequently, the outstanding electrocatalytic performance toward the CO_2_-to-CO conversion makes Fe-N-C-Si stand out against the previously reported Fe-based and other transition metal-based SACs, and even noble metal-based electrocatalysts (Table S3).

To overcome the mass transfer limitation of CO_2_, the CO_2_RR performances of Fe-N-C-Si and Fe-N-C were further measured in a self-designed flow cell reactor equipped with the gas diffusion electrode (GDE) (Fig. 4D and Fig. S31), in which 1.0 m KOH was used as the electrolyte. According to previous works, the use of alkaline electrolytes can not only accelerate the activation of CO_2_ molecules and the overall reaction rate, but also suppress the competitive HER during CO_2_ electroreduction, thereby further promoting the CO_2_RR performance [51]. As displayed in linear sweep voltammetry (LSV) curves (Fig. 4E), the onset potentials of Fe-N-C-Si and Fe-N-C are further decreased in the flow cell, as compared to those obtained in the H-type cell, while the current densities are significantly increased. Specifically, the onset overpotentials for CO generation are as low as 28 and 22 mV for Fe-N-C-Si and Fe-N-C, respectively. Moreover, it is worth noting that Fe-N-C-Si can deliver the current density over 350 mA cm^−2^ at −0.45 V, much higher than that of Fe-N-C (~290 mA cm^−2^ at −0.60 V). Not only that, the FE_CO_ of Fe-N-C-Si can reach 99.44% at 50 mA cm^−2^ and retain above 95% in the current density range from 2 to 250 mA cm^−2^ (Fig. 4F). Nevertheless, despite possessing a similar maximum (99.11% at 20 mA cm^−2^), the FE_CO_ of Fe-N-C dropped sharply at the current densities exceeding 100 mA cm^−2^, which may be due to the poisoning of the atomic Fe sites by the high CO coverage at high current densities, as well as the flooding of the GDE, consistent with most of the previously reported results [16,50]. Consequently, Fe-N-C-Si is capable of delivering large CO/H_2_ ratios and an incredible *j_CO_* > 230 mA cm^−2^(Fig. S33), much higher than that of Fe-N-C, and almost outperforms all the reported Fe-based CO_2_RR electrocatalysts (Table S4). To the best of our knowledge, previous Fe-based electrocatalysts can hardly provide a *j_CO_* > 150 mA cm^−2^, or even 200 mA cm^−2^. Therefore, we believe that Fe-N-C-Si becomes the best Fe-based CO_2_RR electrocatalyst by far. Additionally, it has been reported that the hydrophilicity of GDEs has a significant effect on their CO_2_RR performance [52]. However, the similar water contact angles of the GDEs coated with Fe-N-C and Fe-N-C-Si preclude the effect of hydrophilicity on their different CO_2_RR performance (Fig. S32), further illustrating that Si doping-induced electronic structure regulation of the single-atom Fe sites in Fe-N-C-Si is responsible for the boosted CO_2_RR performance, especially at high CO coverage. Meanwhile, characterizations show that the composition, morphology, and structure of Fe-N-C-Si were maintained well during long-term electrolysis at high current density (Figs. S34 to S37).

### Zn-CO_2_ battery performances of catalysts

**Fig. 5.**
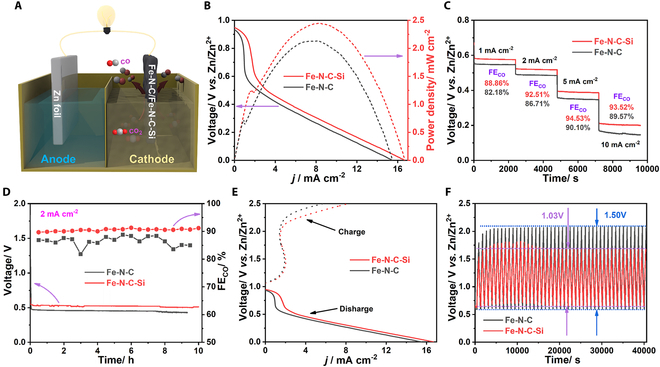
Zn-CO_2_ battery measurements. (A) Schematic diagram of the ZCB assembled with Fe-N-C-Si or Fe-N-C as the cathode. (B) Discharge polarization and power density curves. (C) Discharge curves and corresponding FE_CO_ at the current densities of 1, 2, 5, and 10 mA cm^−2^. (D) Galvanostatic discharge curves and corresponding FE_CO_ at 2 mA cm^−2^. (E) Discharge and charge polarization curves. (F) Galvanostatic discharge–charge cycling curves at 0.5 mA cm^−2^.

Encouraged by the excellent CO_2_RR performance, a rechargeable ZCB with Fe-N-C-Si as the cathode was assembled to further assess the feasibility of its practical implementation (Fig. 5A). As shown in Fig. 5B, the ZCB driven by Fe-N-C-Si provides a maximum power density of 2.44 mW cm^−2^ at 0.30 V with a current density of 8.2 mA cm^−2^, much better than those of Fe-N-C (2.13 mW cm^−2^ and 8.0 mA cm^−2^). Moreover, although both Fe-N-C-Si- and Fe-N-C-based ZCBs display considerably stable voltage responses at various discharge current densities ranging from 1 to 10 mA cm^−2^ (Fig. 5C), Fe-N-C-Si always presents a higher output potential at each discharge current density. Meanwhile, the CO_2_RR products during the discharge process were also detected. For Fe-N-C-Si-based ZCB, the stable and high FE_CO_ of 88.86% to 94.53% can be obtained at diverse discharge current densities of 1 to 10 mA cm^−2^, which are superior to Fe-N-C-based ZCB. Furthermore, as displayed in Fig. 5D, the output voltage and high FE_CO_ of Fe-N-C-Si-based ZCB can be well maintained for more than 10 h during the discharge process at a current density of 2 mA cm^−2^, suggesting its excellent durability. Additionally, it is generally known that CO_2_RR and oxygen evolution reaction (OER) occur at the cathode during the discharge and charge process, respectively [48,53,54]. Therefore, the OER performances of Fe-N-C-Si and Fe-N-C were also evaluated. As shown in Fig. S38, Fe-N-C-Si exhibits a higher OER activity compared to Fe-N-C, in which the Fe-N_4_ sites and/or N-doped carbon should be the active sites for OER [48,54]. Moreover, Fig. 5E shows the discharge and charge polarization curves of Fe-N-C-Si- and Fe-N-C-based ZCBs. It can be clearly observed that the voltage gap of Fe-N-C-Si-based ZCB is narrower than that of the Fe-N-C-based one. Consequently, Fe-N-C-Si-based ZCBs exhibit an impressive rechargeable durability with a charge–discharge gap of only 1.03 V at a current density of 0.5 mA cm^−2^ during continuous operation for at least 40,000 s (Fig. 5F), far superior to that of Fe-N-C-based ZCBs. To sum up, the outstanding performance of Fe-N-C-Si-based ZCBs makes it almost the best among the current state-of-the-art ZCBs (Table S5).

### Electrocatalytic CO_2_RR mechanism analysis

**Fig. 6.**
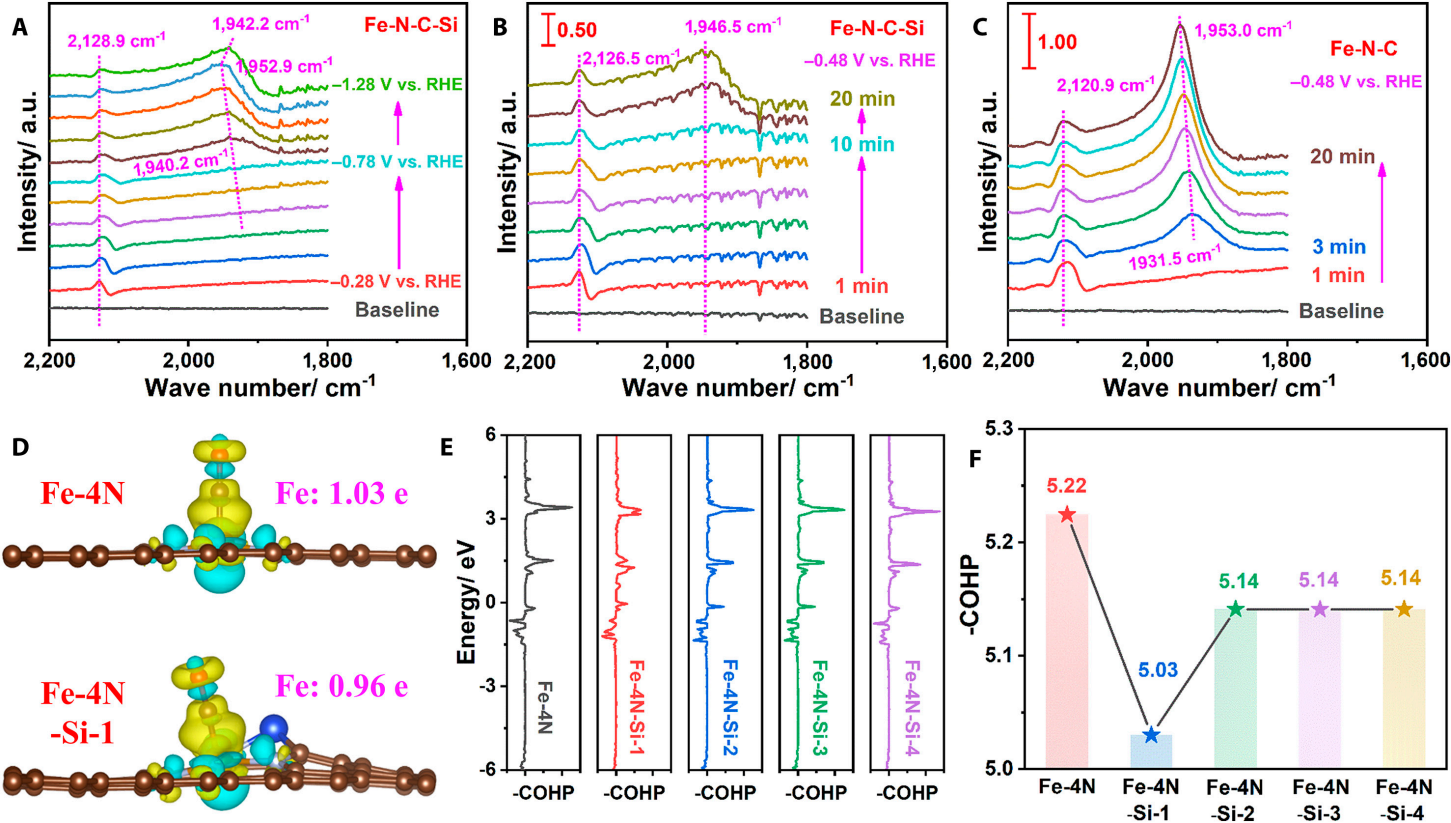
Electrocatalytic CO_2_RR mechanism analysis. (A) In situ ATR-IR spectra of Fe-N-C-Si recorded while stepping the potentials in CO_2_-saturated 0.5 m KHCO_3_. Real-time ATR-IR spectra of (B) Fe-N-C-Si and (C) Fe-N-C collected at −0.28 V in CO_2_-saturated 0.5 m KHCO_3_. (D) Differential charge densities of Fe-4N and Fe-4N-Si-1 configurations after adsorbing the *CO intermediate to the single Fe atom. Yellow and cyan isosurfaces show the electron accumulation and depletion, respectively (isosurface value = 0.0026 e Å^−3^). (E) –COHP curves and (F) values for the interaction between Fe and C atoms after the adsorption of the *CO intermediate.

To further reveal the underlying reaction mechanism over Fe-N-C-Si and Fe-N-C, the in situ ATR-IR spectroscopy was employed to detect the reaction intermediates. It has been widely reported that *COOH is the key intermediate for CO generation [22,55]. However, the absence of the IR signals at around 1,400 cm^−1^ suggests that the conversion of *COOH into *CO species is very fast over both samples (Figs. S39 to S41), which is consistent with the theoretical calculation results mentioned above [55,56]. Moreover, as shown in Fig. 6A and Fig. S40, the peak at around 2,130 cm^−1^ appeared at applied potentials more negative than −0.28 V for both samples, which is attributed to the generation of the bridge-bonded *CO species [39] or/and the presence of N-C triple bonds in the N-doped carbon [22]. Therefore, another peak between 1,940 and 1,960 cm^−1^, assigned to the linear-bonded *CO species [22], was chosen to more accurately track the generation of CO, which appeared at applied potentials more negative than −0.78 V and −0.88 V for Fe-N-C and Fe-N-C-Si, respectively, suggesting that the *CO intermediate is easier to desorb from Fe-N-C-Si, as compared to Fe-N-C. Additionally, the desorption of the *CO intermediate was further studied by the time-dependent in situ ATR-IR spectra at −0.48 V. As displayed in Fig. 6B and C and Fig. S41, the peak with a low intensity at 1,946.5 cm^−1^ appeared after 10 min for Fe-N-C-Si, while for Fe-N-C, the peak with a high intensity at 1,953.0 cm^−1^ appeared only after 1 min. With prolonged time, the intensity of the peak increased rapidly until it becomes steady, illustrating that the *CO intermediate desorption is more difficult on Fe-N-C, which was further confirmed by the higher CO desorption temperature in CO temperature-programmed desorption measurement, as compared with Fe-N-C-Si (Fig. S42) [39].

To deeply understand the adsorption/desorption of intermediates during the CO_2_RR process, charge density distribution analysis upon the adsorption of *COOH, *CO, and *H intermediates in the proposed 5 configurations was also preformed via DFT calculations (Fig. 6D and Figs. S43 to S47). The corresponding results show that more electrons are localized around the Fe center in the configurations with Si doping, which will induce a modulated electronic structure of the atomic Fe sites, in good agreement with the previously mentioned XPS and XANES results. Meanwhile, Bader charge analysis further confirms the lower oxidation state of Fe sites upon the adsorption of *COOH, *CO, and *H intermediates on the Si-incorporated configurations (Table S6), which helps to donate more electrons to facilitate CO desorption on Fe sites [34]. Besides, the interaction between Fe and C atoms after the adsorption of the *CO intermediate was further assessed via the crystal orbital Hamilton population (COHP) analysis [57]. As shown in Fig. 6E and F and Table S7, compared with the pristine Fe-4N configuration, Si doping induces smaller energy gaps between bonding (negative COHP on the right) and antibonding (positive COHP on the left) states, as well as the longer bonding distances of Fe sites and the *CO intermediate, demonstrating the weakened Fe-C bond, which would result in the weaker *CO adsorption and thus favor its desorption [57,58]. Overall, both experimental and theoretical results confirm that the incorporation of Si atoms in Fe-N-C-Si could substantially favor the key pathway of *CO intermediate desorption on the atomic Fe sites, which ultimately boosts the CO_2_RR performance.

## Discussion

In summary, a Fe-based SAC with in situ Si doping was directly prepared via high-temperature pyrolysis of a Fe, Si-containing MOF precursor. Notably, the as-obtained Fe-N-C-Si exhibits an unprecedented CO_2_RR performance, capable of simultaneously delivering a high FE_CO_ (>95%) and industrial-level *j_CO_* (230.0 mA cm^−2^) at an ultralow overpotential of 300 mV, which is far superior to the Fe-N-C counterpart without Si doping and also outperforms the ever-reported Fe-based single-atom electrocatalysts. Furthermore, the assembled rechargeable ZCB with Fe-N-C-Si as the cathode exhibits a narrow charge–discharge voltage gap of 1.03 V at 0.5 mA cm^−2^ and achieves a maximum power density of 2.44 mW cm^−2^ at 0.30 V with a current density of 8.2 mA cm^−2^, as well as high FE_CO_ (>90%) and stability, ranking the highest among the state-of-the-art ZCBs. The in situ ATR-IR spectroscopy combined with DFT calculations determined that the incorporation of Si atoms modulates the electronic structure of the single-atom Fe sites, which obviously favors the desorption of the *CO intermediate under high CO coverage to ease the site poisoning problem, thereby dramatically improving the CO_2_RR performance, especially at high current densities. The ingenious Si-doping strategy put forward in this work will inspire corresponding researchers to design and prepare Si-doped SACs by using similar MOF precursors (Fig. S48). Meanwhile, this work also provides an electronic structure engineering toward adjustable adsorption/desorption behaviors of intermediates to promote the electrocatalytic performances of SACs.

## Materials and Methods

### Chemicals

Zn(NO_3_)_2_·6H_2_O, KHCO_3_, TEOS, anhydrous methanol, and hydrofluoric acid (HF, 40 wt%) were purchased from Sinopharm Chemical Reagent Co. Ltd. 2-MeIM was obtained from Aladdin Reagent. Ethylenediaminetetraacetic acid ferric sodium salt (EDTA-FeNa) and Nafion solution (5 wt%) were purchased from Alfa Aesar. All the chemicals were used as obtained without further purification. Deionized H_2_O was used in all experiments.

### Synthesis of Fe-N-C-Si

Fe-N-C-Si was synthesized via the following 2-step process (Fig. 2A). A Fe, Si-containing MOF precursor (ZnFe-ZIF-Si) was prepared in the first step. Briefly, 4.0 mmol Zn(NO_3_)_2_·6H_2_O and 0.2 mmol EDTA-FeNa were dissolved in 35.0 ml of H_2_O to form solution A. Meanwhile, 24.0 mmol 2-MeIM and 0.3 ml of TEOS were dissolved in 30.0 ml of methanol to form solution B. Then, solution B was poured into solution A and stirred at room temperature for 12 h. Afterward, ZnFe-ZIF-Si was obtained via centrifugation and washed 3 times with methanol and vacuum dried at room temperature overnight. Then, the as-prepared ZnFe-ZIF-Si was placed in a tube furnace and heated at 800 °C for 1 h, 900 °C for 1 h, and 1,000 °C for 1 h successively under Ar atmosphere. After naturally cooling to room temperature, the product was treated with diluted HF solution to remove the possible generated SiO_2_ and metal nanoparticles. After washing several times with deionized water and anhydrous ethanol, the Fe-N-C-Si electrocatalyst was obtained after drying at 60 °C overnight.

### Synthesis of Fe-N-C

Fe-N-C was synthesized similar to that of Fe-N-C-Si, except that TEOS was not added during the preparation of ZnFe-ZIF.

### Physical characterizations

The x-ray diffraction patterns of the samples were collected on a Rikagu Miniflex 600 Benchtop x-ray diffraction instrument with Cu Kα radiation. N_2_ adsorption–desorption isotherm and the BET surface area measurements were measured by using a Belsorp-max instrument at liquid nitrogen temperature (77 K) after dehydration under vacuum at 120 °C for 12 h, while CO_2_ adsorption isotherms were collected under ambient temperature after dehydration under vacuum at 120 °C for 12 h. The metal contents of the catalysts were analyzed using ICP-OES on an ULTIMA 2 ICP Optical Emission Spectrometer. The Raman spectra were recorded in a LabRAM HR Raman microscope with a 532-nm laser. SEM characterization was performed on a Carl Zeiss Sigma 300 instrument. TEM and high-resolution TEM images of the samples were obtained using a FEI Tecnai G^2^ F30 instrument. The atomic dispersion of Fe atoms was detected by JEOL ARM200 F aberration-corrected HAADF-STEM. XPS analysis was measured on a Thermo Fischer ESCALAB 250Xi x-ray photoelectron spectrometer with monochromatic Al Kα radiation (E = 1,486.2 eV), and the binding energies were calibrated by C 1s to 284.8 eV. Fe K-edge XANES and EXAFS spectra were recorded by fluorescence mode at the 1W1B beamline of the Beijing Synchrotron Radiation Facility (BSRF) using a beam line with an energy of 2.5 GeV. The Fe K-edge XANES data were recorded in fluorescence mode. Fe foil, Fe_2_O_3_, and FePc were used as references.

### Electrochemical characterizations

CO_2_RR measurements were first conducted in a proton exchange membrane (Nafion 117) separated H-type cell connected to an electrochemical workstation (CHI 760e), in which saturated Ag/AgCl and Pt mesh were used as the reference electrode and the counter electrode, respectively. CO_2_-saturated 0.5 m KHCO_3_ was used as electrolyte. CO_2_ with a flow rate of 20.0 sccm (standard cubic centimeter per minute) flowed through the electrolyte during electrolysis. To prepare the working electrode, 5.0 mg of sample was dispersed in a solution of H_2_O (600 μl), isopropanol (350 μl), and 5 wt% Nafion solution (50 μl) by sonication to form a homogeneous ink. Then, 200 μl of ink was drop-casted onto a carbon paper sized 1.0 × 1.0 cm^2^ to obtain a catalyst loading amount of 1.0 mg cm^−2^. All the measured potentials were converted to reversible hydrogen electrodes (RHE): E_RHE_ = E_Ag/AgCl_ + 0.0591 × pH + 0.197 (V). LSV curves were recorded at a scan rate of 10 mV s^−1^. The electrochemical impedance spectroscopy (EIS) was recorded with the frequency ranging from 0.01 Hz to 10^5^ Hz at the AC amplitude of 5 mV.

The flow cell tests were carried out in a self-designed reactor with gas, cathode, and anode chambers (Fig. 4D and Fig. S31). The saturated Ag/AgCl reference electrode was inserted into the cathode chamber. Fe-N-C- or Fe-N-C-Si-loaded GDE and Ni foam with a working area of 1.0 cm^−2^ were used as the cathode and the anode, respectively. KOH (1.0 m; pH ≈ 14.0) was used as electrolyte and was circulated with a rate of 5.0 ml min^−1^ through both the cathode and anode chambers. A piece of anion exchange membrane (Fumasep FAA-PK-130) was employed to separate the cathode and anode chambers. CO_2_ gas with a flow rate of 20.0 sccm was directly fed to the backside of the cathode GDE.

**Note:** The electrochemical data collected in the H-type cell are not *iR* compensated, while those obtained in the flow cell are *iR* corrected.

### Rechargeable Zn-CO_2_ battery measurements

The rechargeable Zn-CO_2_ battery measurements were conducted in a bipolar membrane separated H-type cell, in which CO_2_-saturated 1.0 m KHCO_3_ and 6.0 m KOH containing 0.2 m Zn(CH_3_COO)_2_ were used as the catholyte and the anolyte, respectively. CO_2_ with a flow rate of 20.0 sccm flowed through the catholyte during measurements. A mechanical polished zinc plate and a catalyst-loaded (1.0 mg cm^−2^) carbon paper were applied as the anode and the cathode, respectively.

### Product analysis

During CO_2_RR and rechargeable Zn-CO_2_ battery measurements, gas products (CO and H_2_) were quantified with a gas chromatograph (Agilent 7820A), which was equipped with a thermal conductivity detector and a flame ionization detector. Ar was used as the carrier gas.

## Data Availability

All data needed to evaluate the conclusions in the paper are present in the paper and Supplementary Materials. Additional data that are related to this paper may be requested from the authors.

## Supplementary Materials

Supplementary 1 1. Supplementary Methods. 1.1. In situ ATR-IR measurements. 1.2 Density functional theory calculation details. 2. Supplementary Figures. Fig. S1. PXRD patterns of ZnFe-ZIF and ZnFe-ZIF-Si. Fig. S2. SEM images of (A, B) ZnFe-ZIF and (C, D) ZnFe-ZIF-Si. Fig. S3. (A) N_2_ sorption isotherms and (B) micropore size distribution curves of ZnFe-ZIF and ZnFe-ZIF-Si. Fig. S4. SEM images of (A, B) Fe-N-C and (C, D) Fe-N-C-Si. Fig. S5. (A, B) TEM, (C) HAADF-STEM, and (D to H) EDX elemental mapping images of Fe-N-C. Fig. S6. PXRD patterns of Fe-N-C and Fe-N-C-Si. Fig. S7. (A) Raman and (B) EPR spectra of Fe-N-C and Fe-N-C-Si. Fig. S8. (A) N_2_ sorption isotherms, (B) micropore, and (C) mesopore size distribution curves of Fe-N-C and Fe-N-C-Si. Fig. S9. XPS survey spectra of Fe-N-C and Fe-N-C-Si. Fig. S10. The relative contents of N species in Fe-N-C and Fe-N-C-Si. Fig. S11. XPS Si 2p spectra of Fe-N-C-Si with different Ar etching durations. Fig. S12. EXAFS K-space spectrum and the corresponding fitting curve of Fe-N-C-Si. Fig. S13. (A, C) EXAFS k-space spectra and corresponding fitting curves and (B, D) Fourier-transformed EXAFS spectra and corresponding fitting curves of Fe foil and Fe-N-C. Fig. S14. EXAFS k-space spectra and corresponding fitting curves, and Fourier-transformed EXAFS spectra and corresponding fitting curves of (A, D) Fe-4N-Si-2, (B, E) Fe-4N-Si-3, and (C, F) Fe-4N-Si-4. (G) Comparison of the fitted coordination number and *R*-factor of Fe-N-C-Si-1, Fe-N-C-Si-2, Fe-N-C-Si-3, and Fe-N-C-Si-4. Fig. S15. CO_2_RR efficiency of Fe-N-C and Fe-N-C-Si in CO_2_-saturated 0.5 m KHCO_3_. Fig. S16. CO_2_ adsorption isotherms of Fe-N-C and Fe-N-C-Si at 298 K. Fig. S17. (A) Tafel plots, (B) electrochemical impedance plots, (C) capacitive against scan rate, and (D) *j_CO_* normalized by ECSAs of Fe-N-C and Fe-N-C-Si. Fig. S18. CV curves at different scan rates of (A) CP, (B) Fe-N-C, and (C) Fe-N-C-Si. Fig. S19. PXRD patterns of Fe-N-C and Fe-N-C-Si after the long-term stability test. Fig. S20. (A) SEM, (B) SEM-EDX, and (C) SEM-EDX mapping images of Fe-N-C after the long-term stability test. Fig. S21. (A to C) TEM and (D to H) EDX mapping images of Fe-N-C after the long-term stability test. Inset in (A) is the corresponding selected area electron diffraction (SAED) pattern. Fig. S22. (A) SEM, (B) SEM-EDX, and (C) SEM-EDX mapping images of Fe-N-C-Si after the long-term stability test. Fig. S23. (A to C) TEM and (D to H) EDX mapping images of Fe-N-C-Si after the long-term stability test. Inset in (A) is the corresponding SAED pattern. Fig. S24. (A) Survey, (B) Fe 2p, (C) N 1s, and (D) Si 2p XPS spectra of Fe-N-C and Fe-N-C-Si after the long-term stability test. Fig. S25. (A) LSV curves of N-C, N-C-Si, Fe-N-C, and Fe-N-C-Si and (B) potential-dependent FE_CO_ of N-C and N-C-Si measured in CO_2_-saturated 0.5 m KHCO_3_. Fig. S26. SEM images of (A) Zn-ZIF, (B) N-C, (C) Zn-ZIF-Si, and (D) N-C-Si. Fig. S27. SEM images of (A) ZnFe-ZIF, (B) ZnFe-ZIF-01Si, (C) ZnFe-ZIF-02Si, (D) ZnFe-ZIF-Si, (E) ZnFe-ZIF-04Si, and (F) ZnFe-ZIF-05Si. Fig. S28. SEM images of (A) Fe-N-C, (B) Fe-N-C-01Si, (C) Fe-N-C-02Si, (D) Fe-N-C-Si, (E) Fe-N-C-04Si, and (F) Fe-N-C-05Si. Fig. S29. (A) PXRD patterns, (B) N_2_ sorption isotherms, (C) micropore, and (D) mesopore size distribution curves of Fe-N-C-Si with different Si contents. Fig. S30. (A) LSV curves and (B) potential-dependent FE_CO_ of Fe-N-C-Si with different Si contents in CO_2_-saturated 0.5 m KHCO_3_. Fig. S31. Photos of the self-designed flow cell in this work. Fig. S32. Photographs of contact angle measurements on the (A) Fe-N-C- and (B) Fe-N-C-Si-based GDEs. Fig. S33. (A) CO/H_2_ ratios at different current densities and (B) overpotential-dependent FE_CO_ and *j_CO_* of Fe-N-C and Fe-N-C-Si measured in flow cell by using 1.0 m KOH as electrolyte. Fig. S34. Stability test of Fe-N-C-Si-based GDE at 200 mA cm^−2^. Fig. S35. (A) SEM, (B) SEM-EDX, and (C) SEM-EDX mapping images of Fe-N-C-Si-based GDE after electrolysis at 200 mA cm^−2^ for 4 h. Fig. S36. (A to C) TEM and (D to H) EDX mapping images of Fe-N-C-Si-based GDE after electrolysis at 200 mA cm^−2^ for 4 h. Inset in (A) is the corresponding SAED pattern. Fig. S37. (A) Survey, (B) Fe 2p, (C) N 1s, and (D) Si 2p XPS spectra of Fe-N-C-Si-based GDE after electrolysis at 200 mA cm^−2^ for 4 h. Fig. S38. (A) LSV curves and (B) potential-dependent FE_CO_ of Fe-N-C-Si for CO_2_RR collected in CO_2_-saturated KHCO_3_ with difference concentrations. (C) OER performances of Fe-N-C and Fe-N-C-Si in CO_2_-saturated KHCO_3_ with difference concentrations. Fig. S39. In situ ATR-IR spectra of Fe-N-C recorded while stepping the potentials in CO_2_-saturated 0.5 m KHCO_3_. Fig. S40. In situ ATR-IR spectra of Fe-N-C-Si recorded while stepping the potentials in CO_2_-saturated 0.5 m KHCO_3_. Fig. S41. Real-time ATR-IR spectra of (A) Fe-N-C and (B) Fe-N-C-Si collected at −0.28 V in CO_2_-saturated 0.5 m KHCO_3_. Fig. S42. CO-TPD data of Fe-N-C and Fe-N-C-Si. Fig. S43. Differential charge densities of Fe-4N after adsorbing the (A, D) *COOH, (B, E) *CO, and (C, F) *H intermediates to the single Fe atom. Fig. S44. Differential charge densities of Fe-4N-Si-1 after adsorbing the (A, D) *COOH, (B, E) *CO, and (C, F) *H intermediates to the single Fe atom. Fig. S45. Differential charge densities of Fe-4N-Si-2 after adsorbing the (A, D) *COOH, (B, E) *CO, and (C, F) *H intermediates to the single Fe atom. Fig. S46. Differential charge densities of Fe-4N-Si-3 after adsorbing the (A, D) *COOH, (B, E) *CO, and (C, F) *H intermediates to the single Fe atom. Fig. S47. Differential charge densities of Fe-4N-Si-4 after adsorbing the (A, D) *COOH, (B, E) *CO, and (C, F) *H intermediates to the single Fe atom. Fig. S48. CO_2_RR performance of (A, B) Co-N-C and Co-N-C-Si and (C, D) Ni-N-C and Ni-N-C-Si measured in an H-type cell. 3. Supplementary Tables Table S1. BET surface areas, pore volumes, and Fe contents (measured by ICP) of Fe-N-C-Si with different Si contents. Table S2. The fitting parameters of Fe K-edge EXAFS data for Fe foil, Fe-N-C, and Fe-N-C-Si. Table S3. Comparison of the electrocatalytic CO_2_RR performance in an H-type cell over Fe-N-C-Si and other carbon-based single-atom electrocatalysts. Table S4. Comparison of the electrocatalytic CO_2_RR performance in a flow cell over Fe-based electrocatalysts. Table S5. Comparison of the performance of the recently reported Zn-CO_2_ batteries. Table S6. Bader charges of a Fe atom in 5 single-atom Fe configurations after *COOH, *CO, and *H intermediates were adsorbed. Table S7. The distances between Fe and C atoms after the adsorption of the *CO intermediate and the corresponding -COHP values in 5 Fe single-atom configurations.
