# Supplementary material for: Si Doping-Induced Electronic Structure Regulation of Single-Atom Fe Sites for Boosted CO2 Electroreduction at Low Overpotentials
Source: Research (Wash D C). 2023 Mar 15;6:0079. doi: 10.34133/research.0079 (PMC10017332; doi:10.34133/research.0079)
Supplement: Supplementary 1 — 1. Supplementary Methods. 1.1. In situ ATR-IR measurements. 1.2 Density functional theory calculation details. 2. Supplementary Figures. Fig. S1. PXRD patterns of ZnFe-ZIF and ZnFe-ZIF-Si. Fig. S2. SEM images of (A, B) ZnFe-ZIF and (C, D) ZnFe-ZIF-Si. Fig. S3. (A) N2 sorption isotherms and (B) micropore size distribution curves of ZnFe-ZIF and ZnFe-ZIF-Si. Fig. S4. SEM images of (A, B) Fe-N-C and (C, D) Fe-N-C-Si. Fig. S5. (A, B) TEM, (C) HAADF-STEM, and (D to H) EDX elemental mapping images of Fe-N-C. Fig. S6. PXRD patterns of Fe-N-C and Fe-N-C-Si. Fig. S7. (A) Raman and (B) EPR spectra of Fe-N-C and Fe-N-C-Si. Fig. S8. (A) N2 sorption isotherms, (B) micropore, and (C) mesopore size distribution curves of Fe-N-C and Fe-N-C-Si. Fig. S9. XPS survey spectra of Fe-N-C and Fe-N-C-Si. Fig. S10. The relative contents of N species in Fe-N-C and Fe-N-C-Si. Fig. S11. XPS Si 2p spectra of Fe-N-C-Si with different Ar etching durations. Fig. S12. EXAFS K-space spectrum and the corresponding fitting curve of Fe-N-C-Si. Fig. S13. (A, C) EXAFS k-space spectra and corresponding fitting curves and (B, D) Fourier-transformed EXAFS spectra and corresponding fitting curves of Fe foil and Fe-N-C. Fig. S14. EXAFS k-space spectra and corresponding fitting curves, and Fourier-transformed EXAFS spectra and corresponding fitting curves of (A, D) Fe-4N-Si-2, (B, E) Fe-4N-Si-3, and (C, F) Fe-4N-Si-4. (G) Comparison of the fitted coordination number and R-factor of Fe-N-C-Si-1, Fe-N-C-Si-2, Fe-N-C-Si-3, and Fe-N-C-Si-4. Fig. S15. CO2RR efficiency of Fe-N-C and Fe-N-C-Si in CO2-saturated 0.5 m KHCO3. Fig. S16. CO2 adsorption isotherms of Fe-N-C and Fe-N-C-Si at 298 K. Fig. S17. (A) Tafel plots, (B) electrochemical impedance plots, (C) capacitive against scan rate, and (D) jCO normalized by ECSAs of Fe-N-C and Fe-N-C-Si. Fig. S18. CV curves at different scan rates of (A) CP, (B) Fe-N-C, and (C) Fe-N-C-Si. Fig. S19. PXRD patterns of Fe-N-C and Fe-N-C-Si after the long-term stability test. Fig. S20 [file research.0079.f1.docx]

**Supporting Information**

**Title**

**Si-doping induced electronic structure regulation of single-atom Fe sites for boosted CO_2_ electroreduction at low overpotentials**

**Authors**

Changsheng Cao^1^, Shenghua Zhou^1,3^, Shouwei Zuo^4^, Huabin Zhang^4^, Bo Chen^5^, Junheng Huang^6^, Xin-Tao Wu^1,2,3^, Qiang Xu^7,8^ and Qi-Long Zhu^1,2,3^

**Affiliations**

^1^State Key Laboratory of Structural Chemistry, Fujian Institute of Research on the Structure of Matter, Chinese Academy of Sciences, Fuzhou, 350002, China

^2^Fujian Science & Technology Innovation Laboratory for Optoelectronic Information of China, Fuzhou, 350108, China.

^3^University of Chinese Academy of Science, Beijing, 100049, China.

^4^KAUST Catalysis Center (KCC), King Abdullah University of Science and Technology (KAUST), Thuwal, 23955-6900, Saudi Arabia.

^5^Department of Chemistry, City University of Hong Kong, Hong Kong, 999077, China.

^6^CAS Key Laboratory of Design and Assembly of Functional Nanostructures, Fujian Provincial Key Laboratory of Nanomaterials, Fujian Institute of Research on the Structure of Matter, Chinese Academy of Sciences, Fuzhou, 350002, China.

^7^Institute for Integrated Cell-Material Sciences (iCeMS), Kyoto University, Kyoto 606-8501, Japan.

^8^Shenzhen Key Laboratory of Micro/Nano-Porous Functional Materials (SKLPM), SUSTech-Kyoto University Advanced Energy Materials Joint Innovation Laboratory (SKAEM-JIL), and Department of Materials Science and Engineering, Southern University of Science and Technology (SUSTech), Shenzhen, 518055, China.

Correspondence should be addressed to Qi-Long Zhu; qlzhu@fjirsm.ac.cn

**1. Supplementary Methods**

*1.1 In situ ATR-IR measurements.* The working electrodes for in situ ATR-IR measurements were fabricated similar to the previous works [1, 2]. Briefly, an Au thin film was chemically deposited onto the reflecting plane of a Si prism, and then drop-coated with the catalyst ink. The catalyst ink was prepared by dispersing 5 mg of sample in a mixture solution containing 1.0 mL H_2_O and 10 μL 5 wt% Nafion solution. The working electrode was mounted in a two-compartment electrochemical cell with a platinum wire and a standard Ag/AgCl electrode as the counter electrode and the reference electrode, respectively. CO_2_-saturated 0.5 m KHCO_3_ was used as the electrolyte. A bare Au film without catalyst loading was served as the background control. The ATR-IR spectra were collected using a Nicolet iS50 FT-IR spectrometer equipped with a MCT detector. The spectral resolution was set to 4 cm^–1^ for all measurements.

*1.2* *Density functional theory calculation details*. In our work, DFT calculations were performed in the Vienna ab initio simulation package (VASP) [3, 4]. The projector augmented wave (PAW) method was adopted to describe interactions between ions and electrons [5]. The generalized gradient approximation (GGA) in the form of Perdew, Burke, Ernzerhof (PBE) was used to describe electron exchange and correlation.[6] The plane-wave basis set along with a kinetic cutoff energy was 400 eV. The Brillouin zones were sampled with 4 × 4 × 1 Monkhorst-Pack meshes. The structures were fully relaxed until the maximum force on each atom was less than –0.02 eV/Å and 10^–5^ eV. A vacuum space of at least 10 Å was inserted along the z direction to avoid any interactions between the periodically repeated images. The van der Waals interaction was considered using the DFT-D3 scheme. The Gibbs free energy (ΔG) was defined as follow:

ΔG = ΔE + ΔE_ZPE_ –TΔS

where ΔE is the reaction energy from the density functional theory calculations, ΔE_ZPE_ and ΔS are the zero point energy difference and the entropy difference between the products and the reactants at room temperature, respectively.

**2. Supplementary Figures**





**Figure S1.** PXRD patterns of ZnFe-ZIF and ZnFe-ZIF-Si.


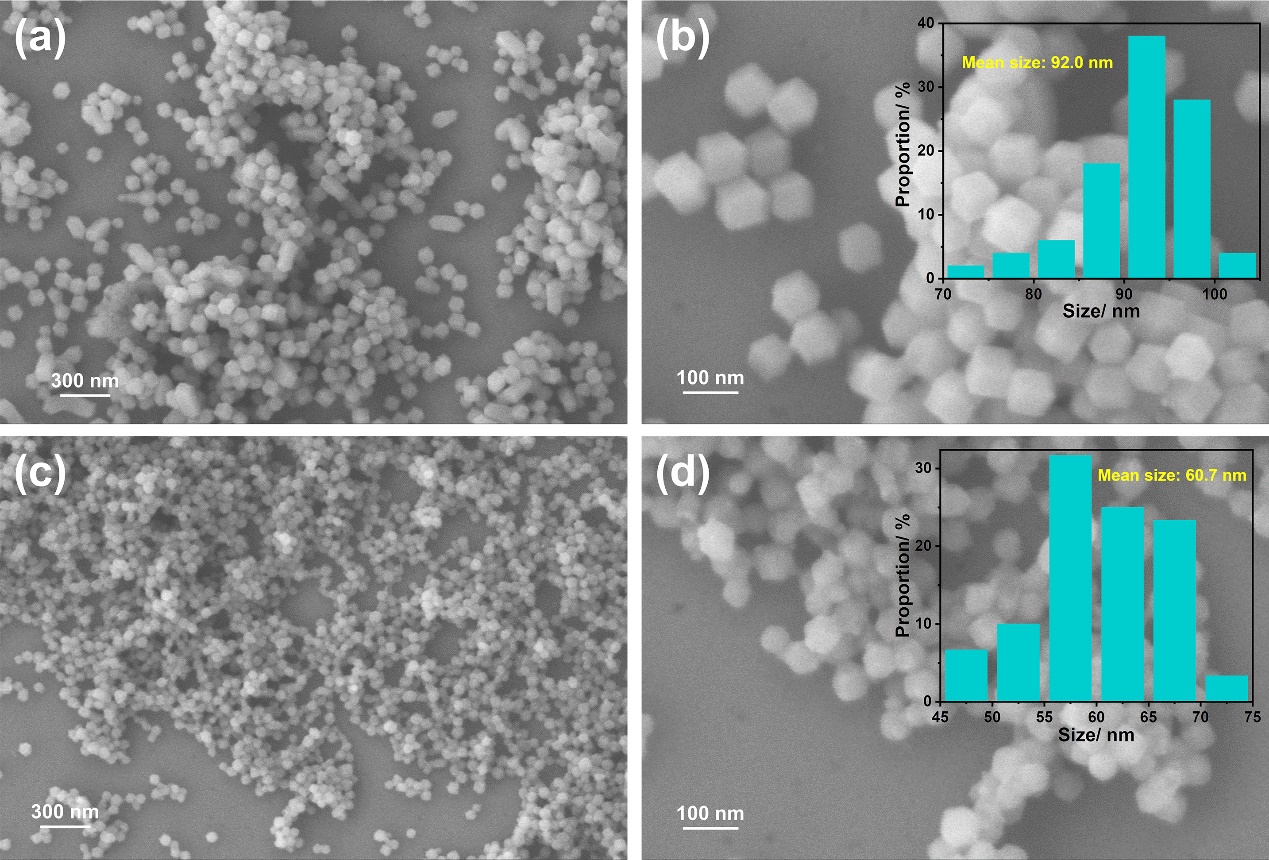


**Figure S2.** SEM images of (a, b) ZnFe-ZIF and (c, d) ZnFe-ZIF-Si.


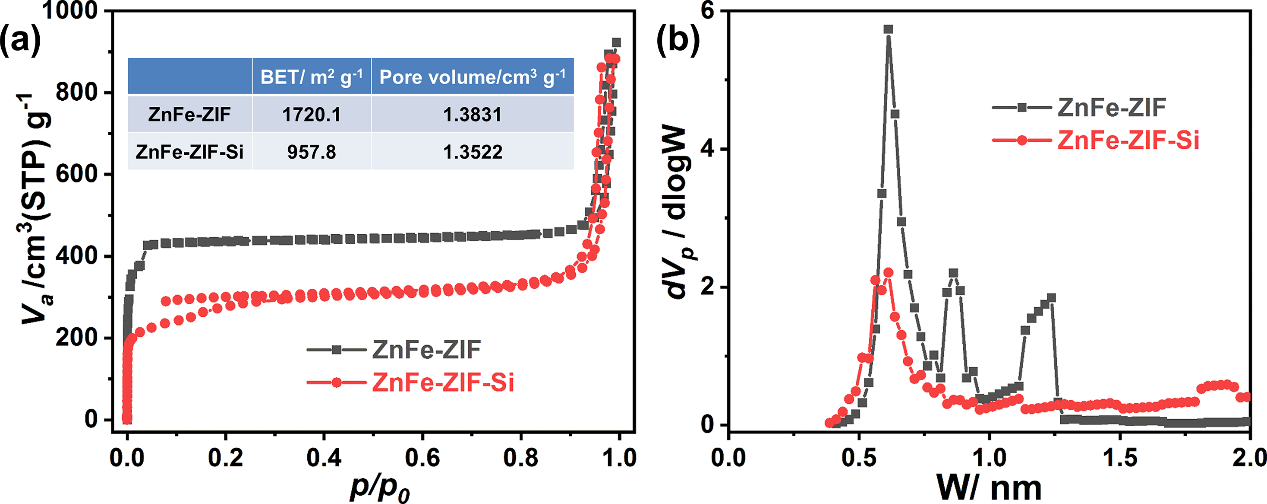


**Figure S3.** (a) N_2_ sorption isotherms and (b) micropore size distribution curves of ZnFe-ZIF and ZnFe-ZIF-Si.


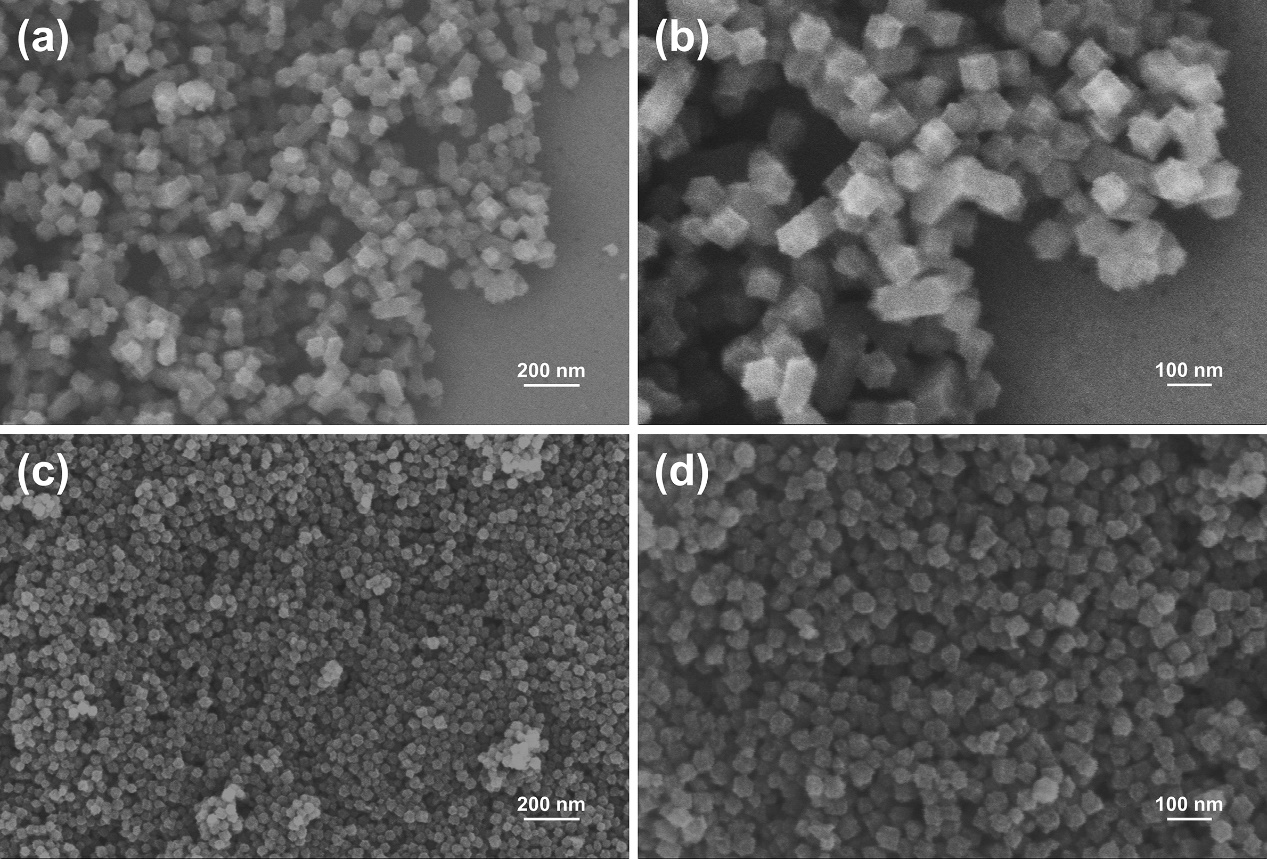


**Figure S4.** SEM images of (a, b) Fe-N-C and (c, d) Fe-N-C-Si.


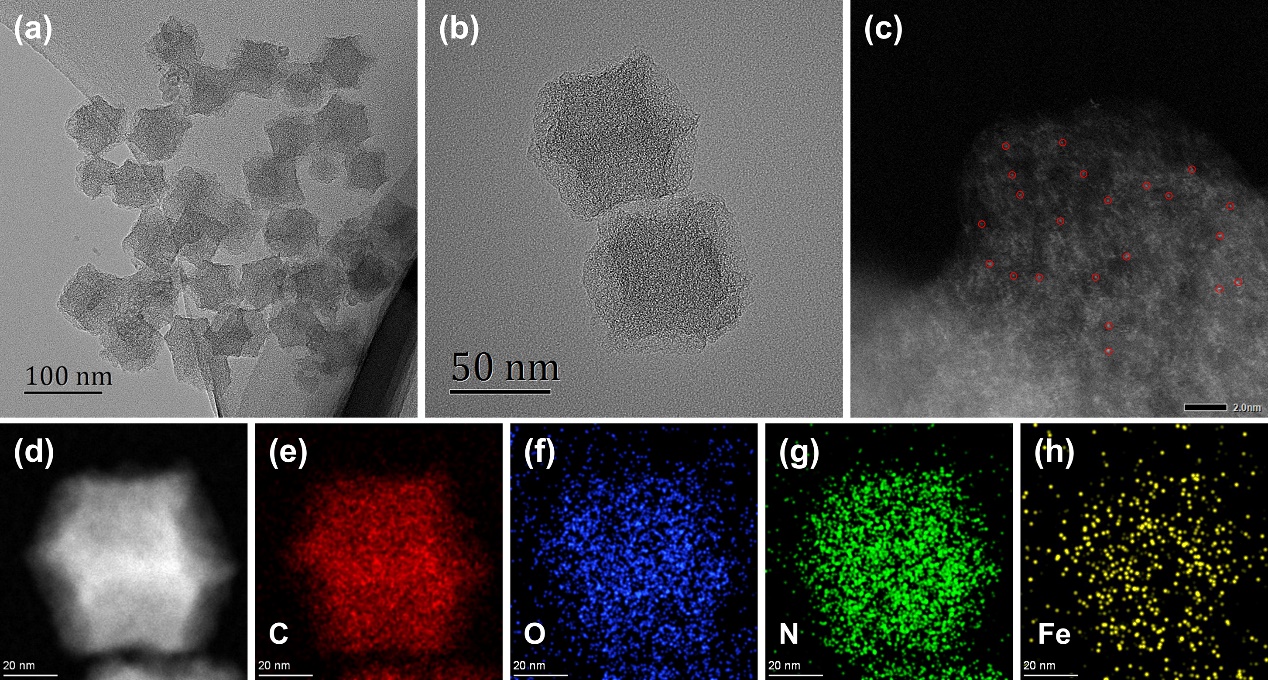


**Figure S5.** (a, b) TEM, (c) HAADF-STEM and (d–h) EDX elemental mapping images of Fe-N-C.





**Figure S6.** PXRD patterns of Fe-N-C and Fe-N-C-Si.


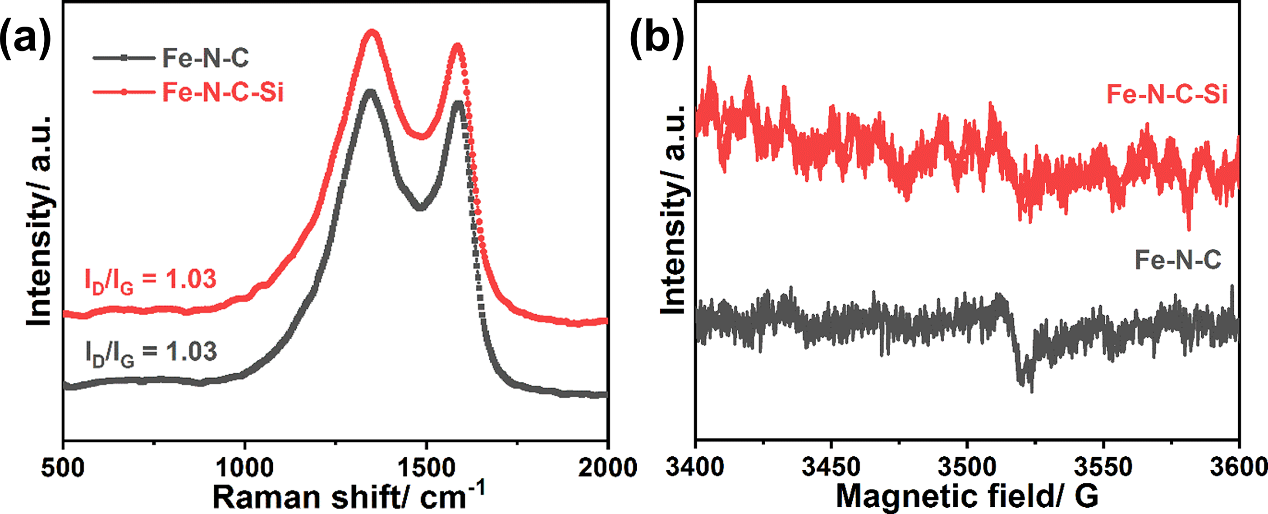


**Figure S7.** (a) Raman and (b) EPR spectra of Fe-N-C and Fe-N-C-Si.


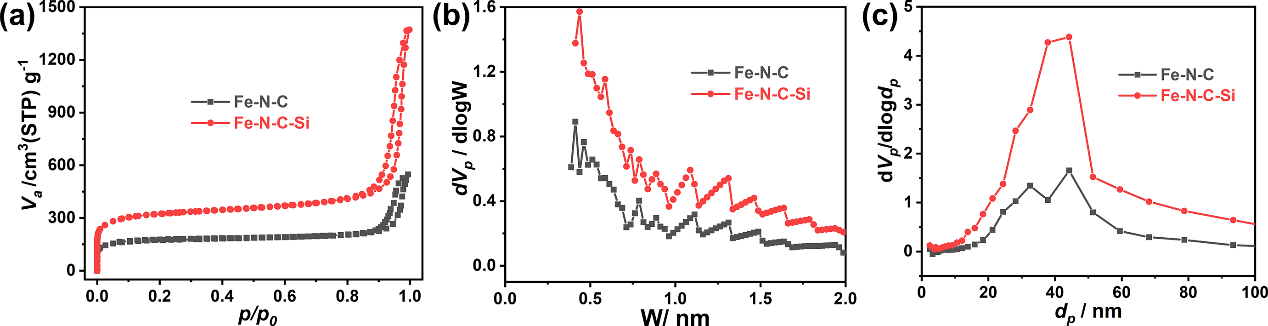


**Figure S8.** (a) N_2_ sorption isotherms, (b) micropore and (c) mesopore size distribution curves of Fe-N-C and Fe-N-C-Si.





**Figure S9.** XPS survey spectra of Fe-N-C and Fe-N-C-Si.





**Figure S10.** The relative contents of N species in Fe-N-C and Fe-N-C-Si.





**Figure S11.** XPS Si 2p spectra of Fe-N-C-Si with different Ar etching durations.





**Figure S12.** EXAFS K-space spectrum and corresponding fitting curve of Fe-N-C-Si.


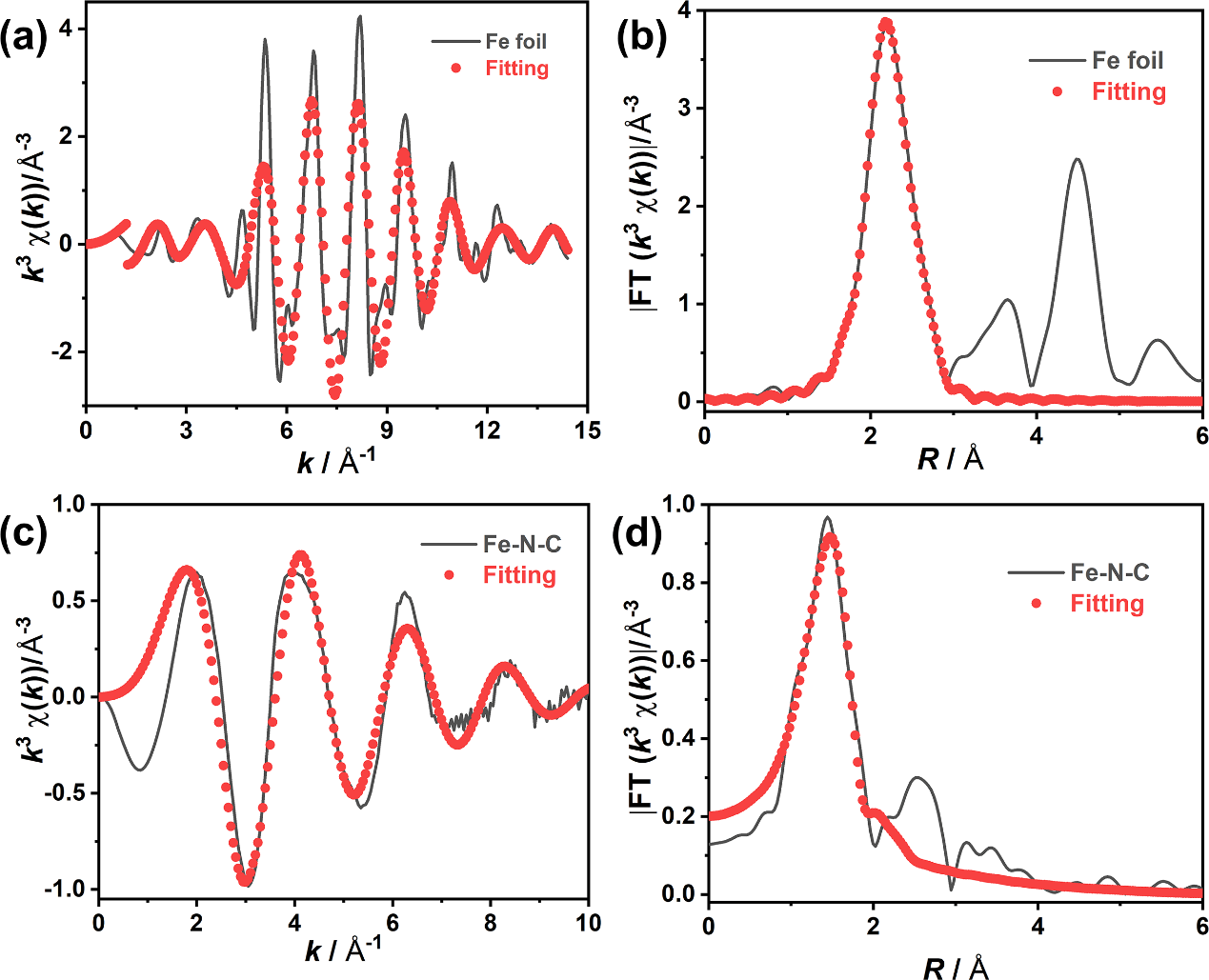


**Figure S13.** (a, c) EXAFS k-space spectra and corresponding fitting curves and (b, d) Fourier transformed EXAFS spectra and corresponding fitting curves of Fe foil and Fe-N-C.


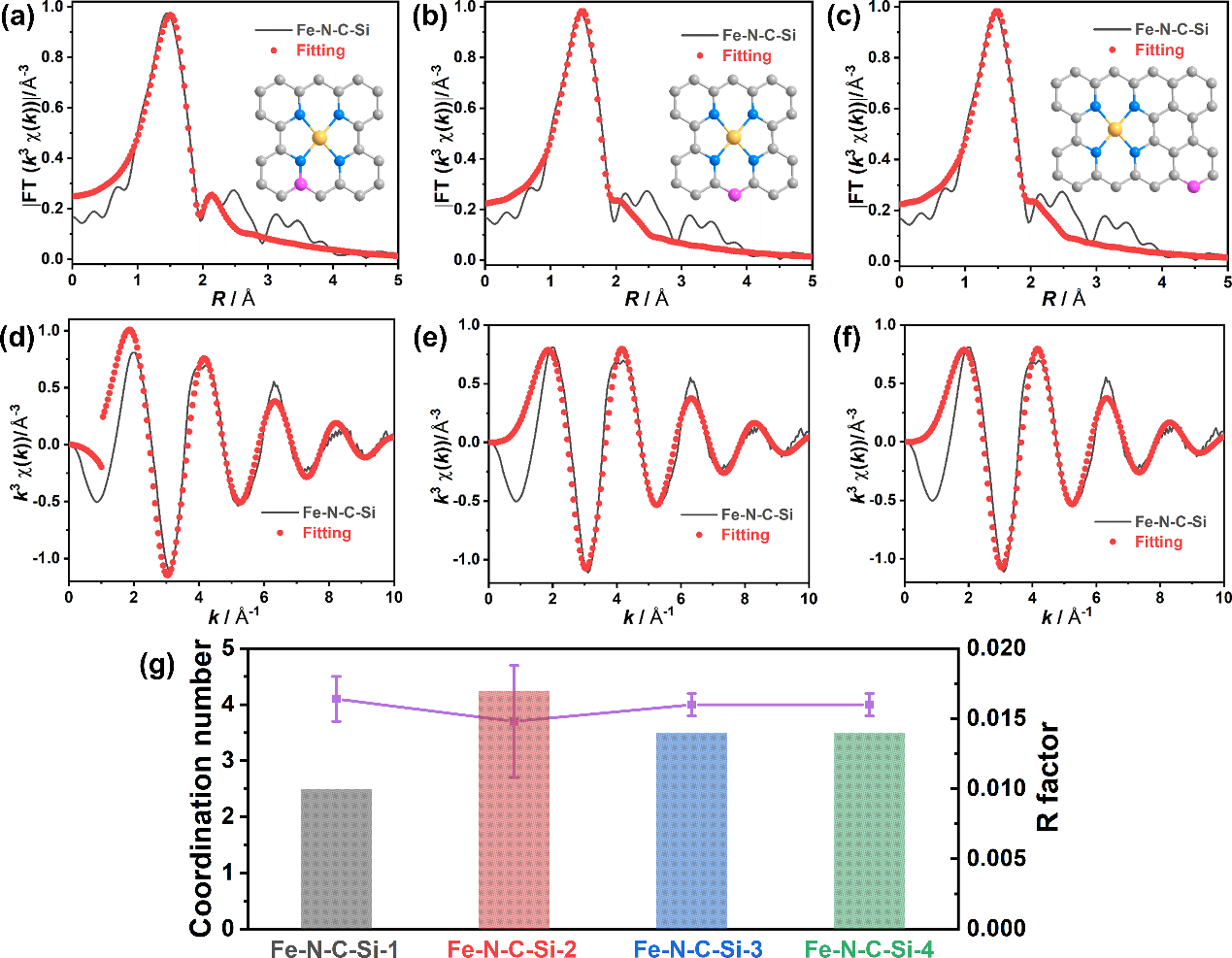


**Figure S14.** EXAFS k-space spectra and corresponding fitting curves, and Fourier transformed EXAFS spectra and corresponding fitting curves of (a, d) Fe-4N-Si-2, (b, e) Fe-4N-Si-3 and (c, f) Fe-4N-Si-4. (g) Comparison of the fitted coordination number and R-factor of Fe-N-C-Si-1, Fe-N-C-Si-2, Fe-N-C-Si-3 and Fe-N-C-Si-4.

Screened from different theoretical models **(Figures 3(g), Figures S12, 14)**, considering the small R-factor, the Fe-N-C-Si-1 model shows the best fitting goodness for the Fe-N-C-Si sample.

**

**

**Figure S15.** CO_2_RR efficiency of Fe-N-C and Fe-N-C-Si in CO_2_-saturated 0.5 m KHCO_3_.

The CO_2_ reduction efficiency of the electrocatalysts can be roughly calculated from the equation: (*j_CO_*_2_ – *j_Ar_*)/ *j_CO_*_2_ * 100%) [7]. As clearly shown in **Figure S15,** Fe-N-C-Si presents the higher CO_2_ reduction efficiency than that for Fe-N-C in the tested potential range.





**Figure S16.** CO_2_ adsorption isotherms of Fe-N-C and Fe-N-C-Si at 298 K.


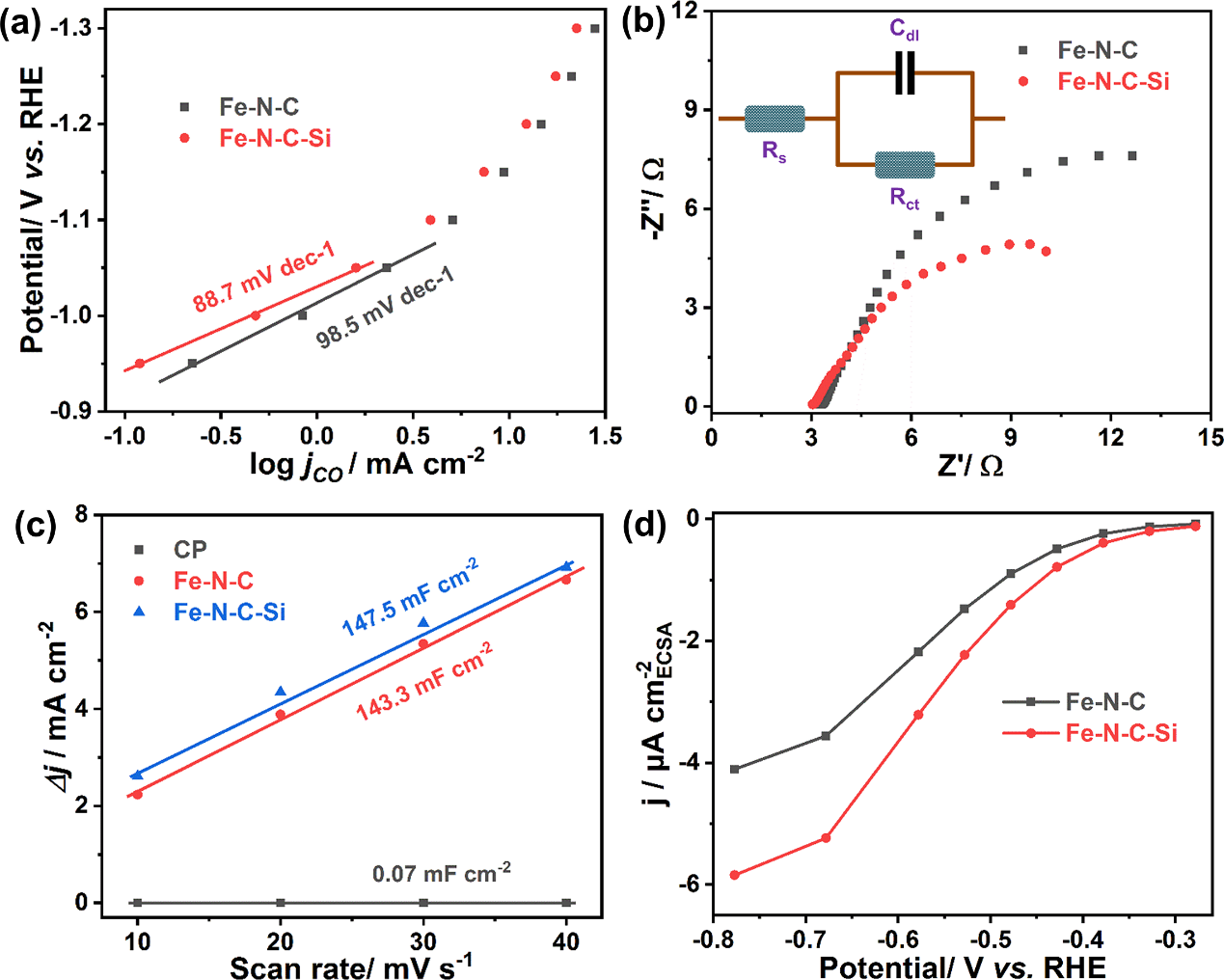


**Figure S17.** (a) Tafel plots, (b) electrochemical impedance plots, (c) capacitive against scan rate and (d) *j_CO_* normalized by ECSAs of Fe-N-C and Fe-N-C-Si.

Tafel plots were obtained according to a previous work, where the collected steady-state *j_CO_* are plotted against applied potentials with 100 % *iR* compensation [8]. As shown in **Figure S17**, Fe-N-C-Si exhibits a lower Tafel slope (88.7 mV dec ^–1^) than that of Fe-N-C (98.5 mV dec ^–1^), indicating its favorable CO_2_RR kinetics. Besides, the Tafel slopes of both samples imply that their rate-determining step (RDS) for CO production should be the first electron transfer process [9].


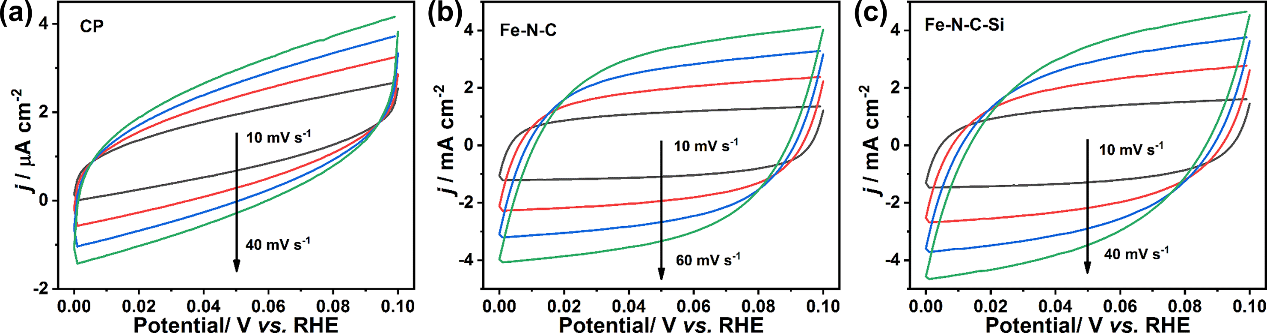


**Figure S18.** CV curves at different scan rates of (a) CP, (b) Fe-N-C and (c) Fe-N-C-Si.





**Figure S19.** PXRD patterns of Fe-N-C and Fe-N-C-Si after long-term stability test.


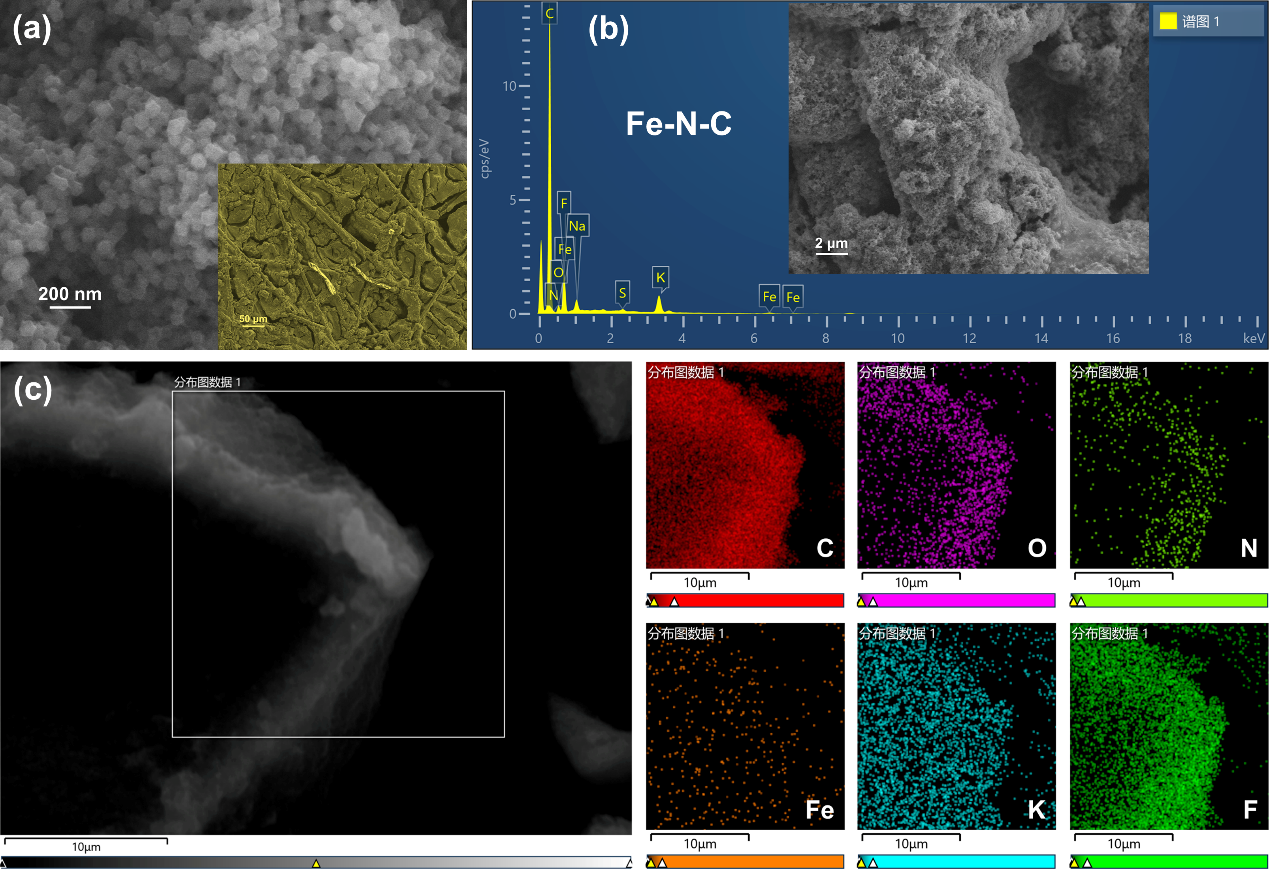


**Figure S20.** (a) SEM, (b)SEM-EDX and (c) SEM-EDX mapping images of Fe-N-C after long-term stability test.

The signals of F and Na shown in SEM-EDX spectra (Figure S20(b)) are originated from the Nafion binder, while the signal of K should be due to the absorption of K^+^ in electrolyte during electrolysis process.


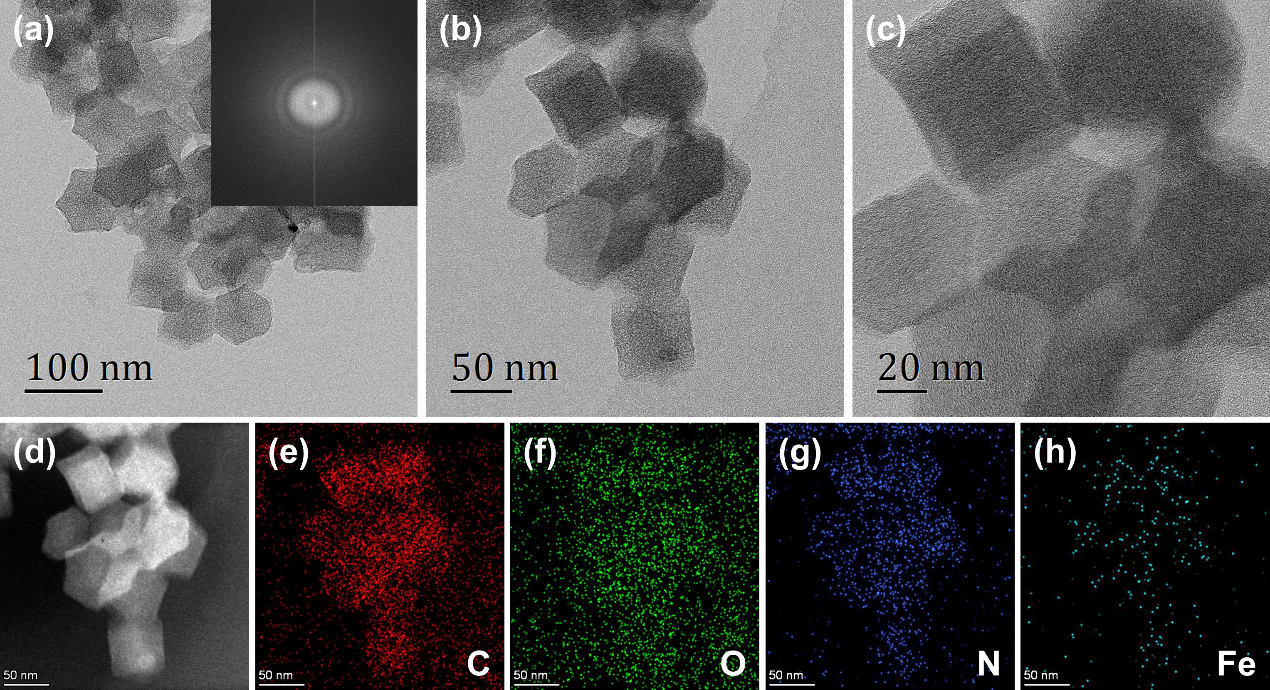


**Figure S21.** (a-c) TEM and (d-h) EDX mapping images of Fe-N-C after long-term stability test. Inset in (a) is the corresponding selected area electron diffraction (SAED) pattern.

The SAED shows a ring-like pattern (Figure S21(a), inset), implying the amorphous carbon structure of the sample.


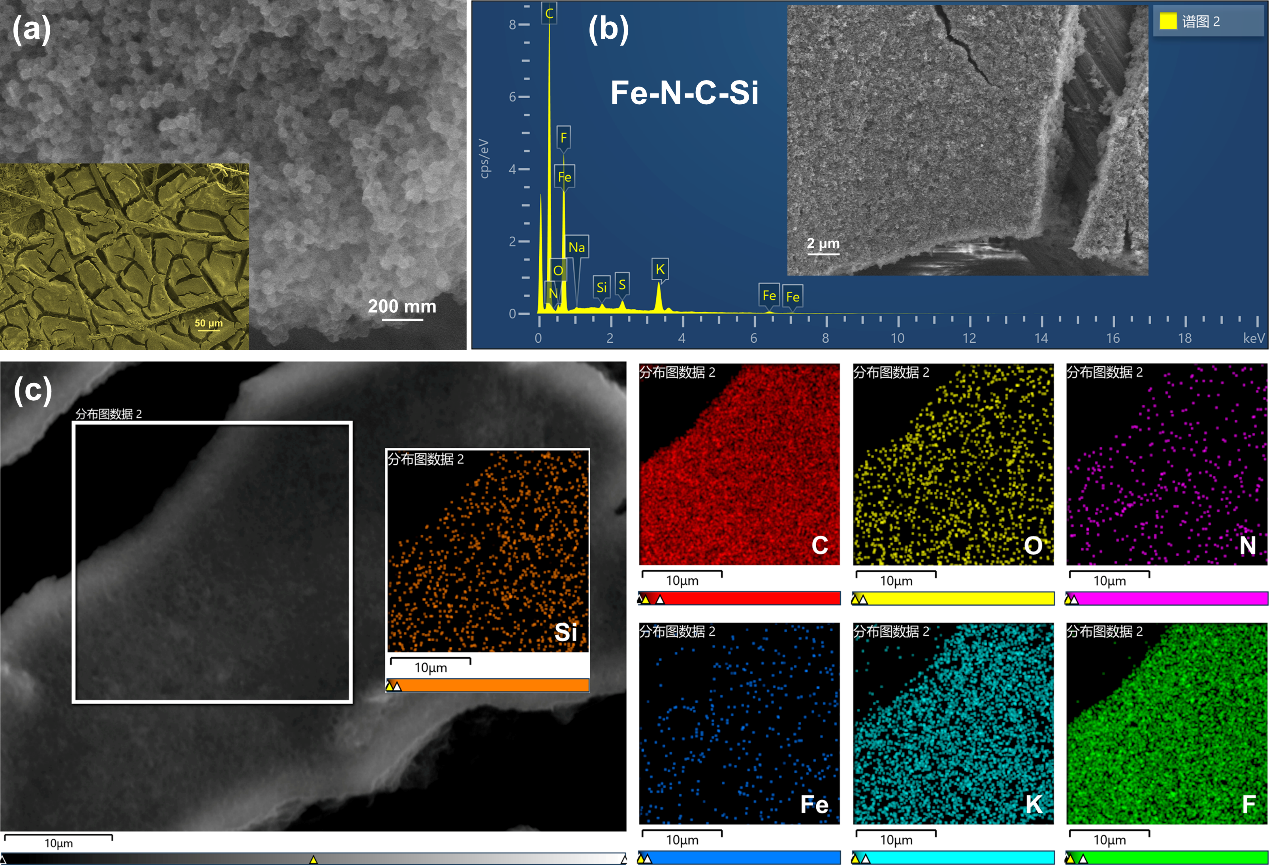


**Figure S22.** (a) SEM, (b)SEM-EDX and (c) SEM-EDX mapping images of Fe-N-C-Si after long-term stability test.


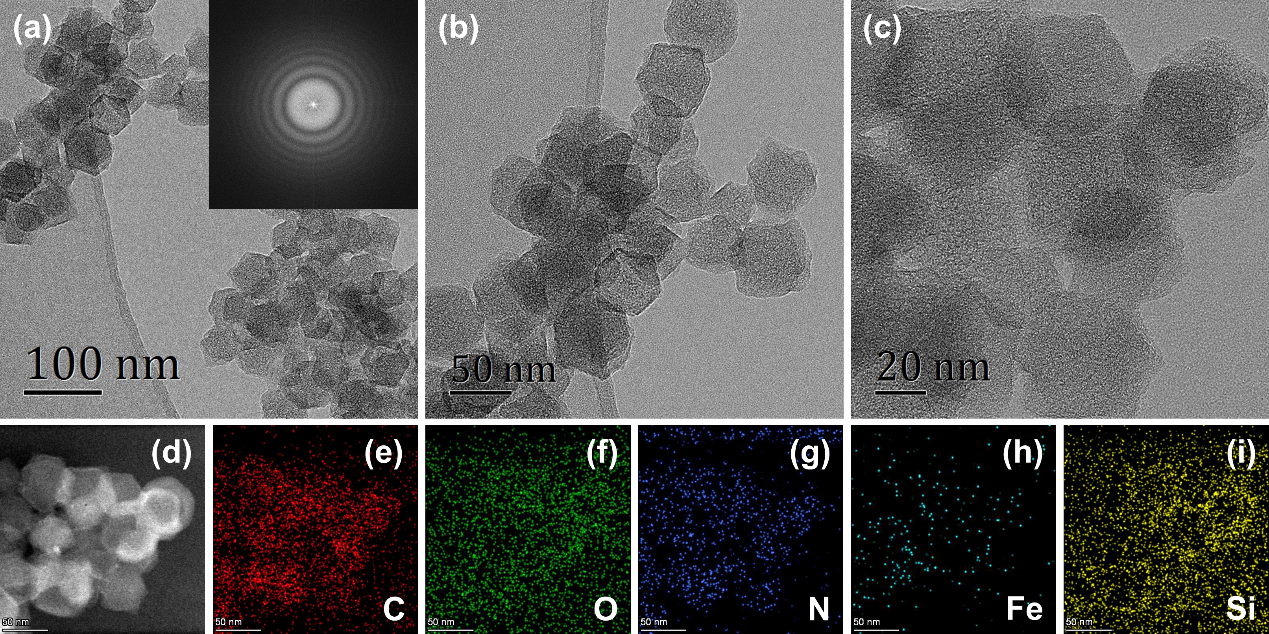


**Figure S23.** (a-c) TEM and (d-h) EDX mapping images of Fe-N-C-Si after long-term stability test. Inset in (a) is the corresponding SAED pattern.


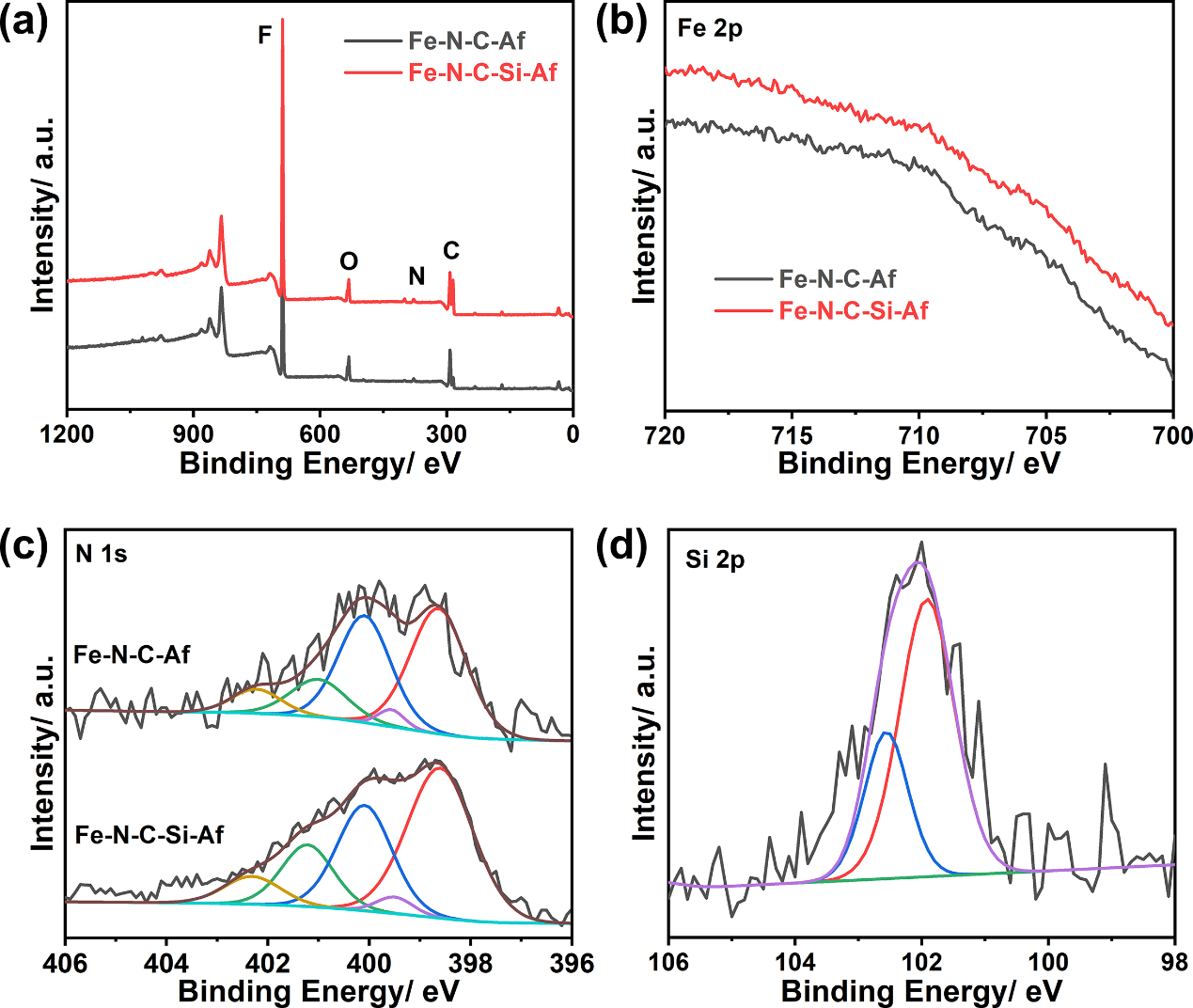


**Figure S24.** (a) Survey, (b) Fe 2p, (c) N 1s and (d) Si 2p XPS spectra of Fe-N-C and Fe-N-C-Si after long-term stability test.

The strong signal of F in survey XPS spectra (Figure S24(a)) is originated from the Nafion binder, while the weak signal of Fe in Fe 2p spectra (Figure S24(b)) is ascribed to the low content.


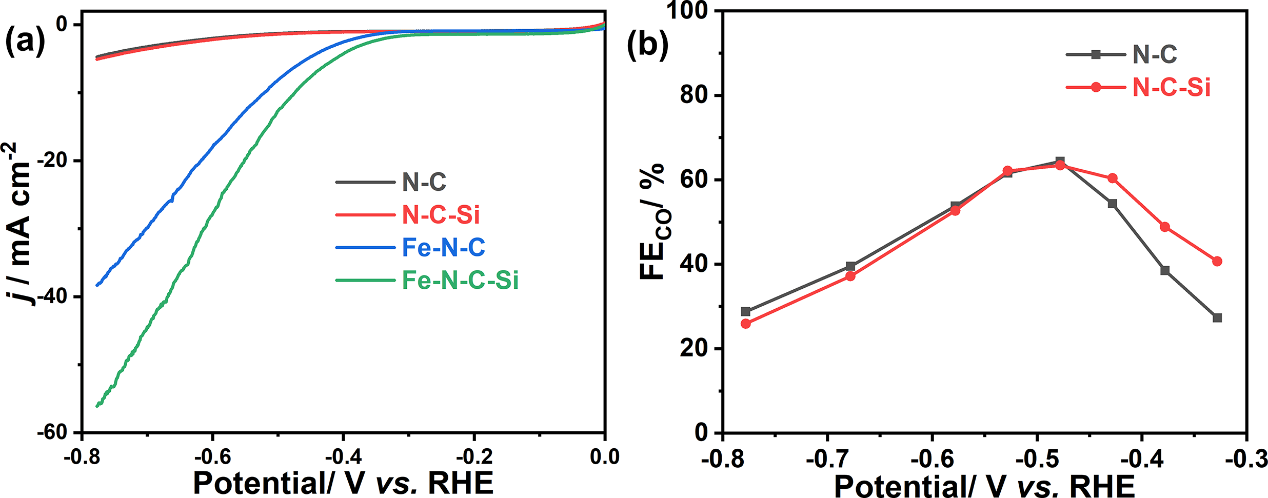


**Figure S25.** (a) LSV curves of N-C, N-C-Si, Fe-N-C and Fe-N-C-Si and (b) potential-dependent FE_CO_ of N-C and N-C-Si measured in CO_2_-saturated 0.5 m KHCO_3_.


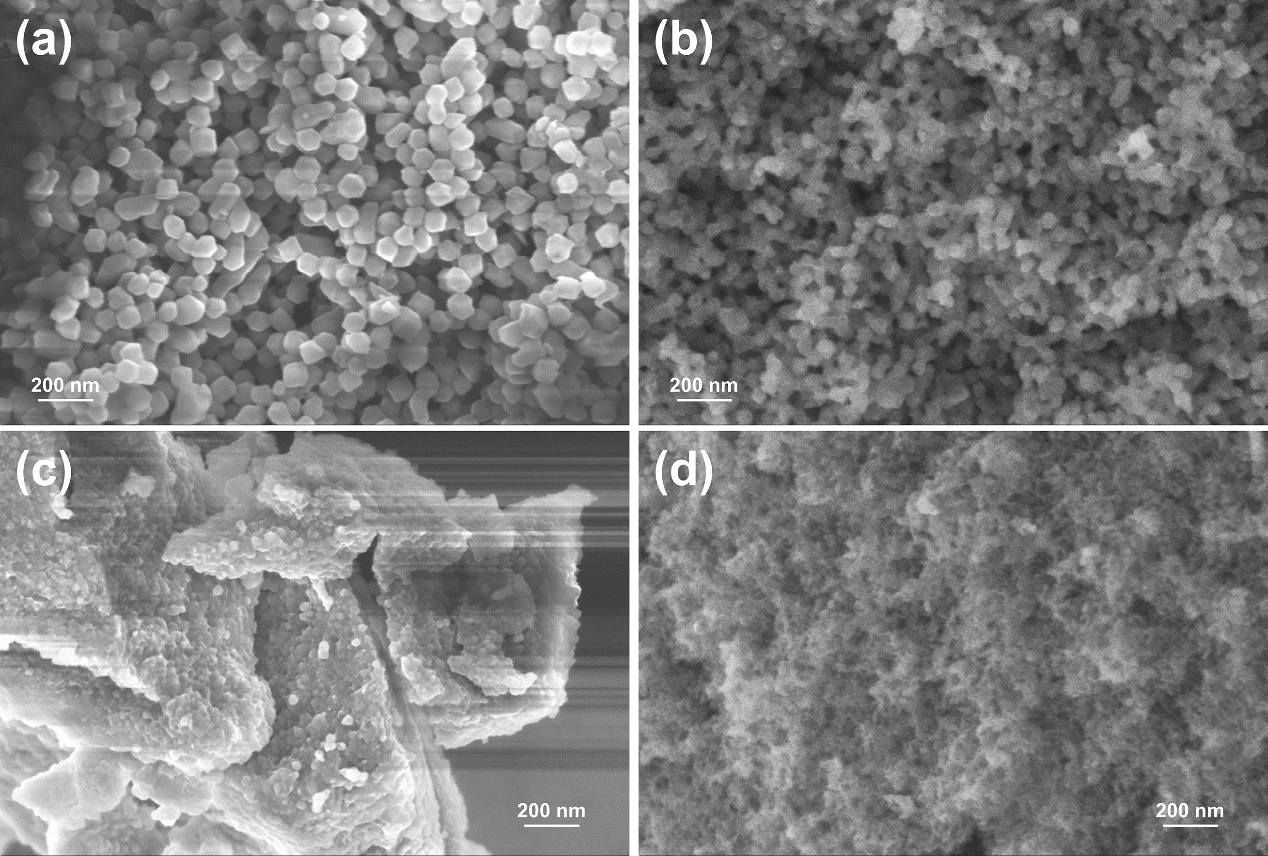


**Figure S26.** SEM images of (a) Zn-ZIF, (b) N-C, (c) Zn-ZIF-Si and (d) N-C-Si.


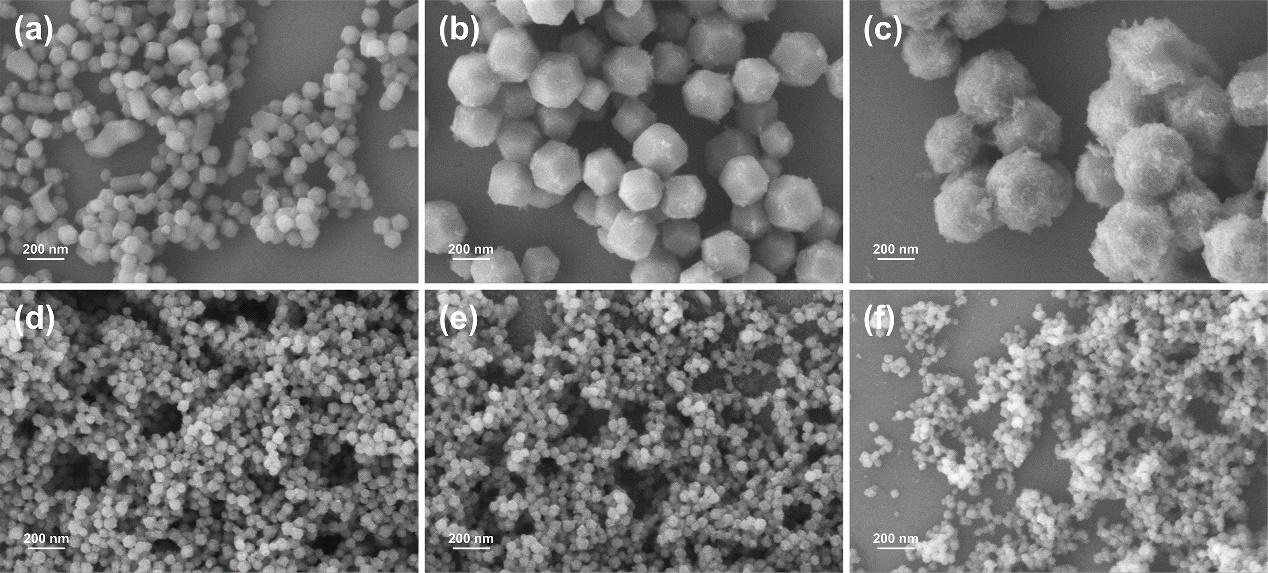


**Figure S27.** SEM images of (a) ZnFe-ZIF, (b) ZnFe-ZIF-01Si, (c) ZnFe-ZIF-02Si, (d) ZnFe-ZIF-Si, (e) ZnFe-ZIF-04Si and (f) ZnFe-ZIF-05Si.

**
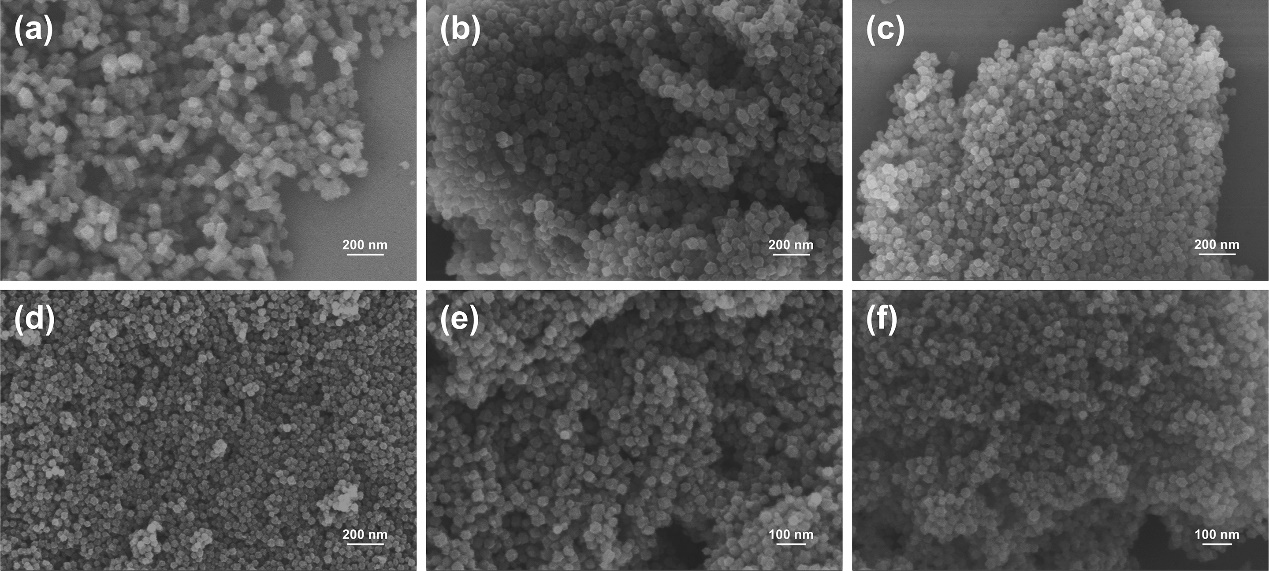
**

**Figure S28.** SEM images of (a) Fe-N-C, (b) Fe-N-C-01Si, (c) Fe-N-C-02Si, (d) Fe-N-C-Si, (e) Fe-N-C-04Si and (f) Fe-N-C-05Si.


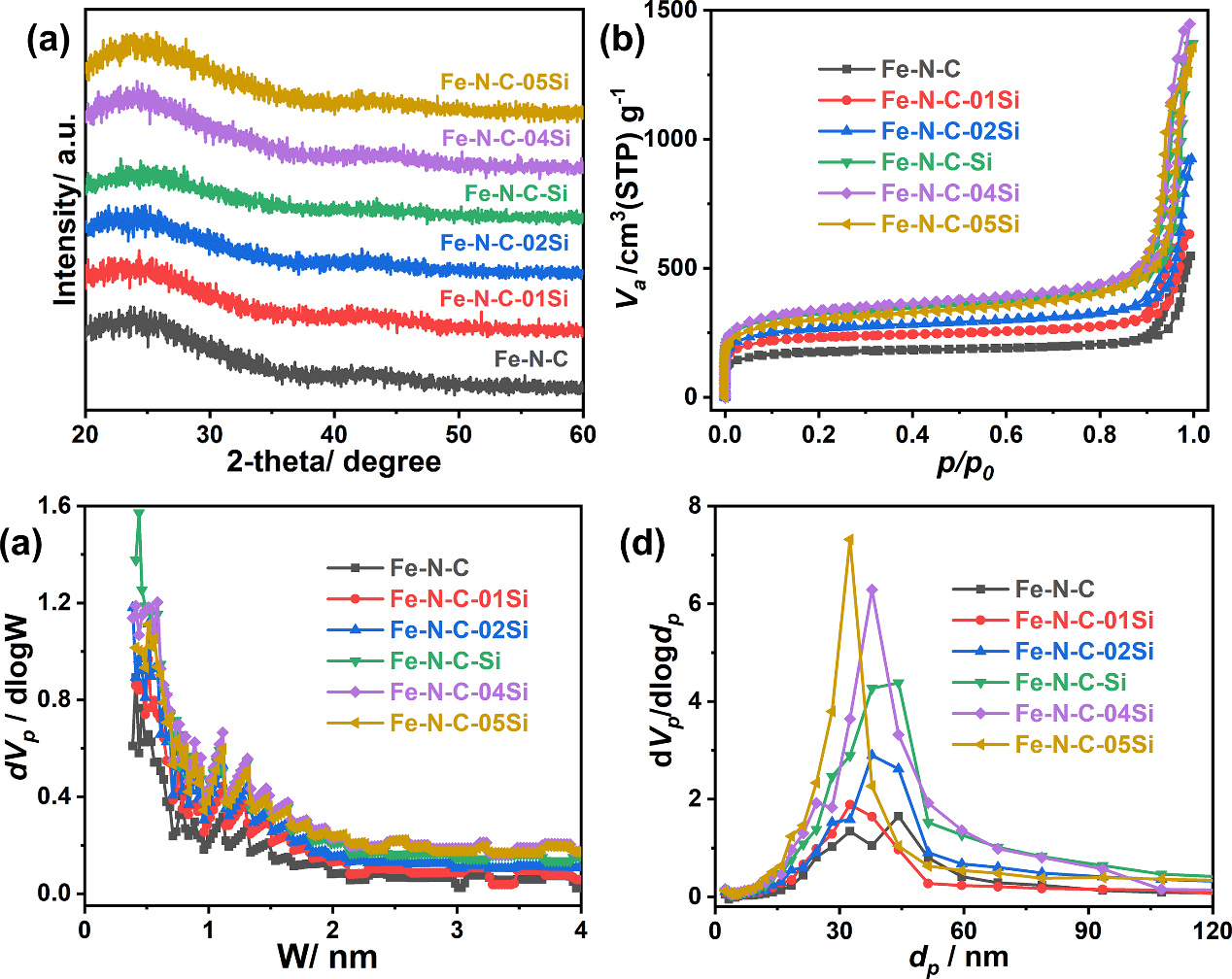


**Figure S29.** (a) PXRD patterns, (b) N_2_ sorption isotherms, (c) micropore and (d) mesopore size distribution curves of Fe-N-C-Si with different Si contents.


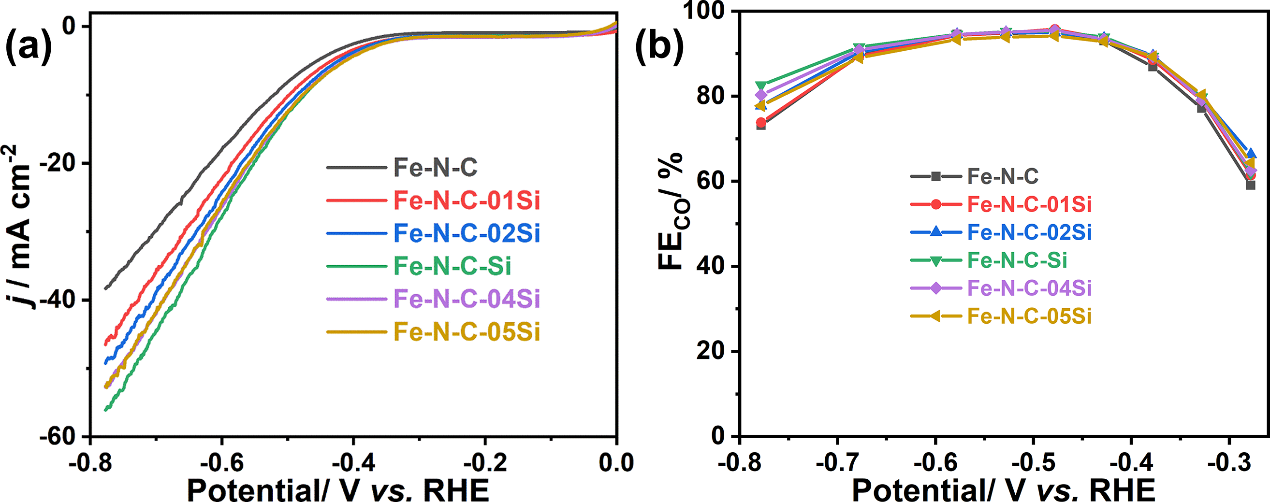


**Figure S30.** (a) LSV curves and (b) potential-dependent FE_CO_ of Fe-N-C-Si with different Si contents in CO_2_-saturated 0.5 m KHCO_3_.


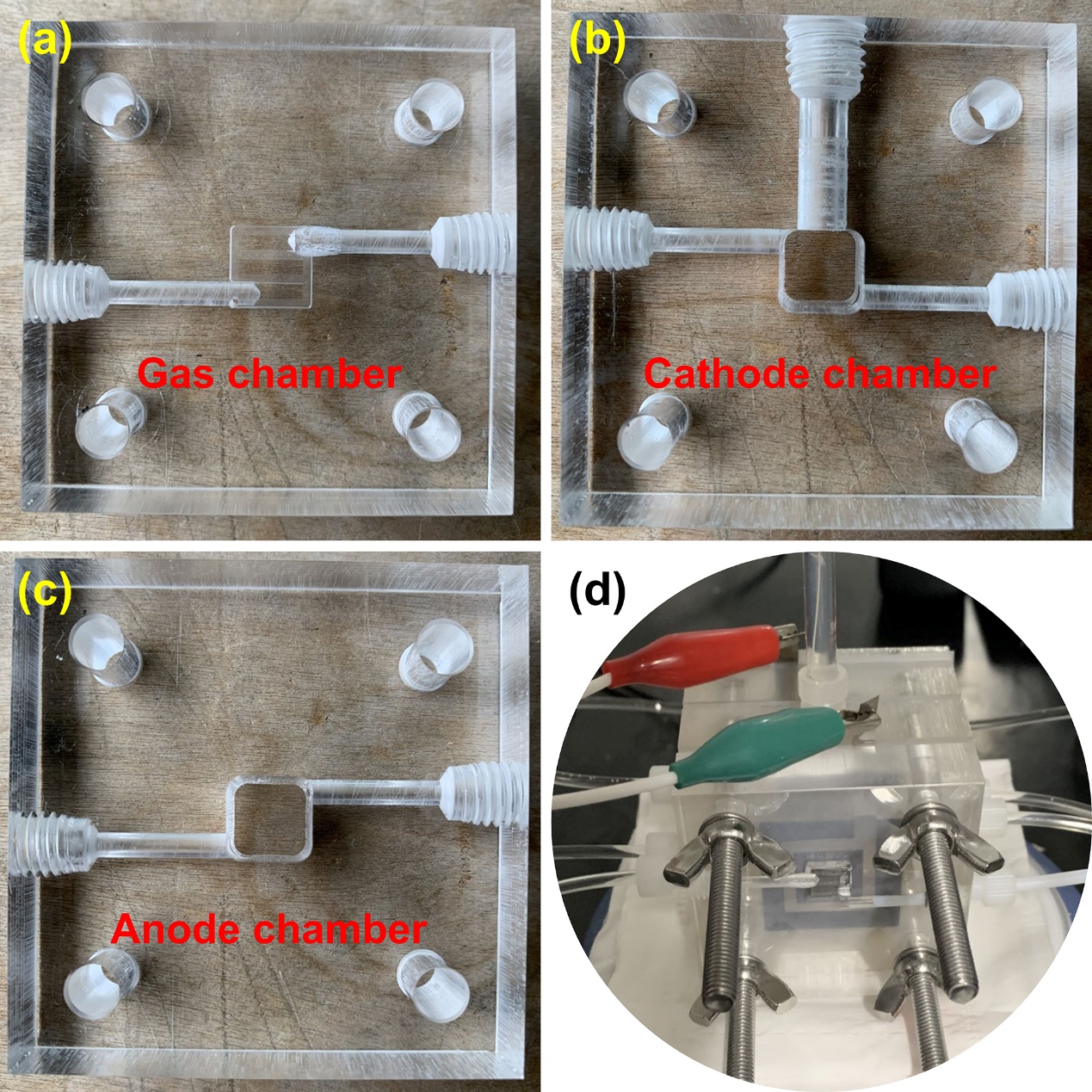


**Figure S31.** Photos of the self-designed flow cell in this work.


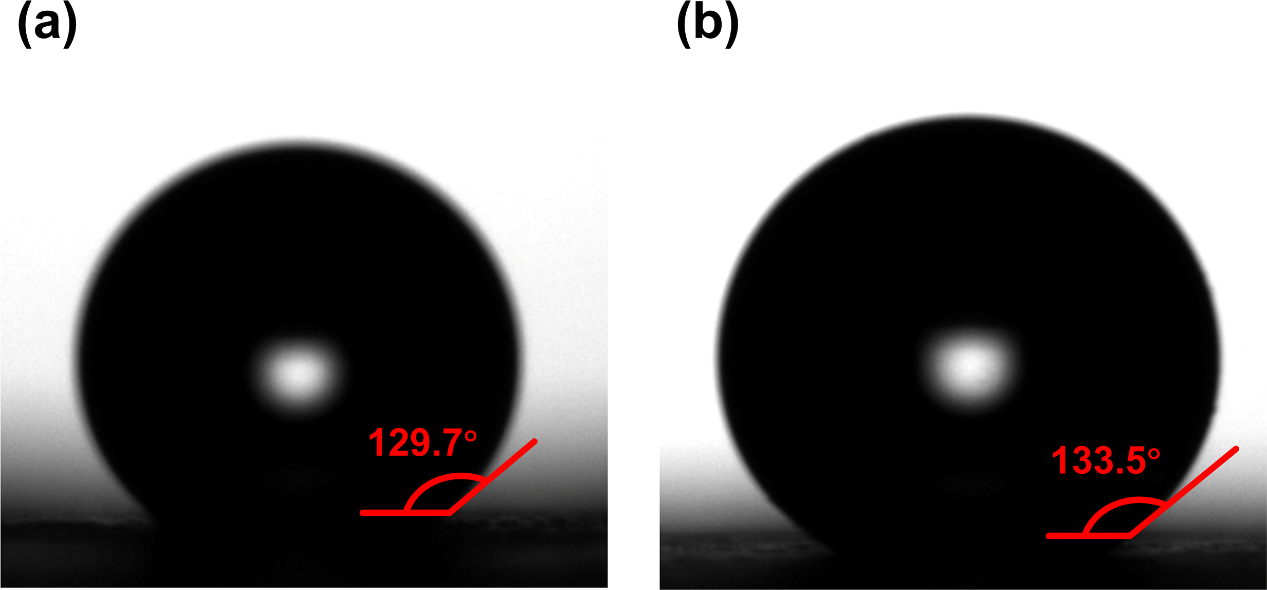


**Figure S32.** Photographs of contact angle measurements on the (a) Fe-N-C and (b) Fe-N-C-Si based GDEs.


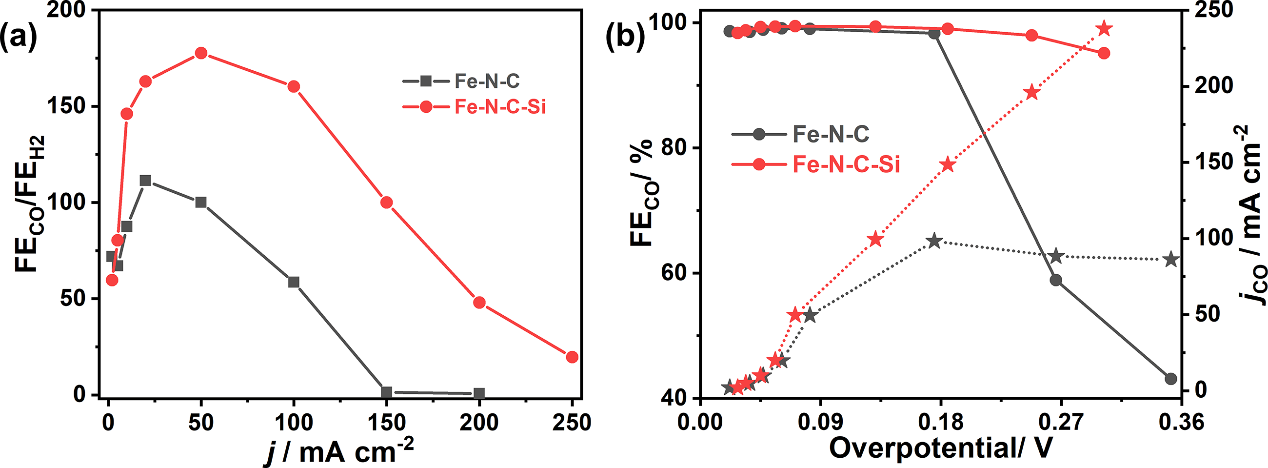


**Figure S33.** (a) CO/H_2_ ratios at different current densities and (b) overpotential-dependent FE_CO_ and *j_CO_* of Fe-N-C and Fe-N-C-Si measured in flow cell by using 1.0 m KOH as electrolyte.





**Figure S34.** Stability test of Fe-N-C-Si based GDE at 200 mA cm^–2^.


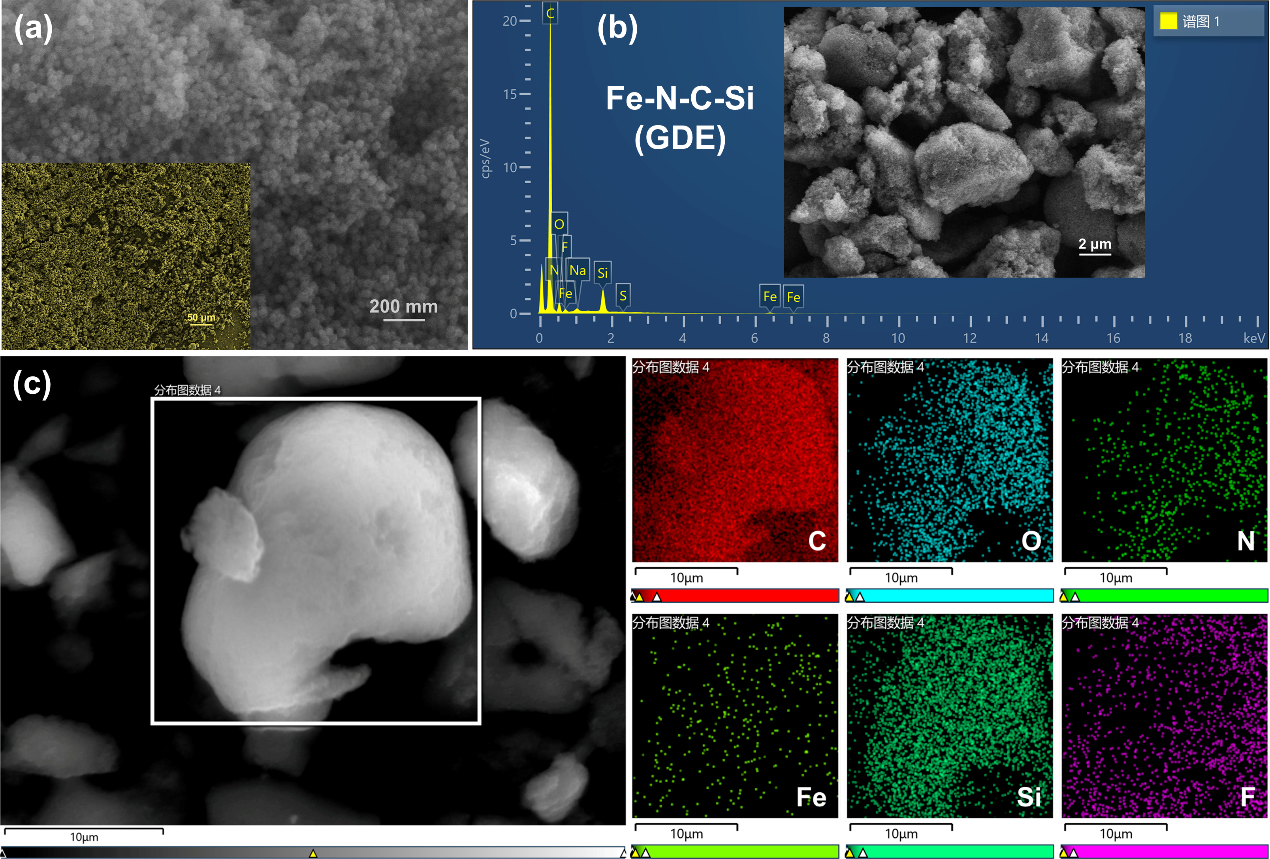


**Figure S35.** (a) SEM, (b)SEM-EDX and (c) SEM-EDX mapping images of Fe-N-C-Si based GDE after electrolysis at 200 mA cm^–2^ for 4h.


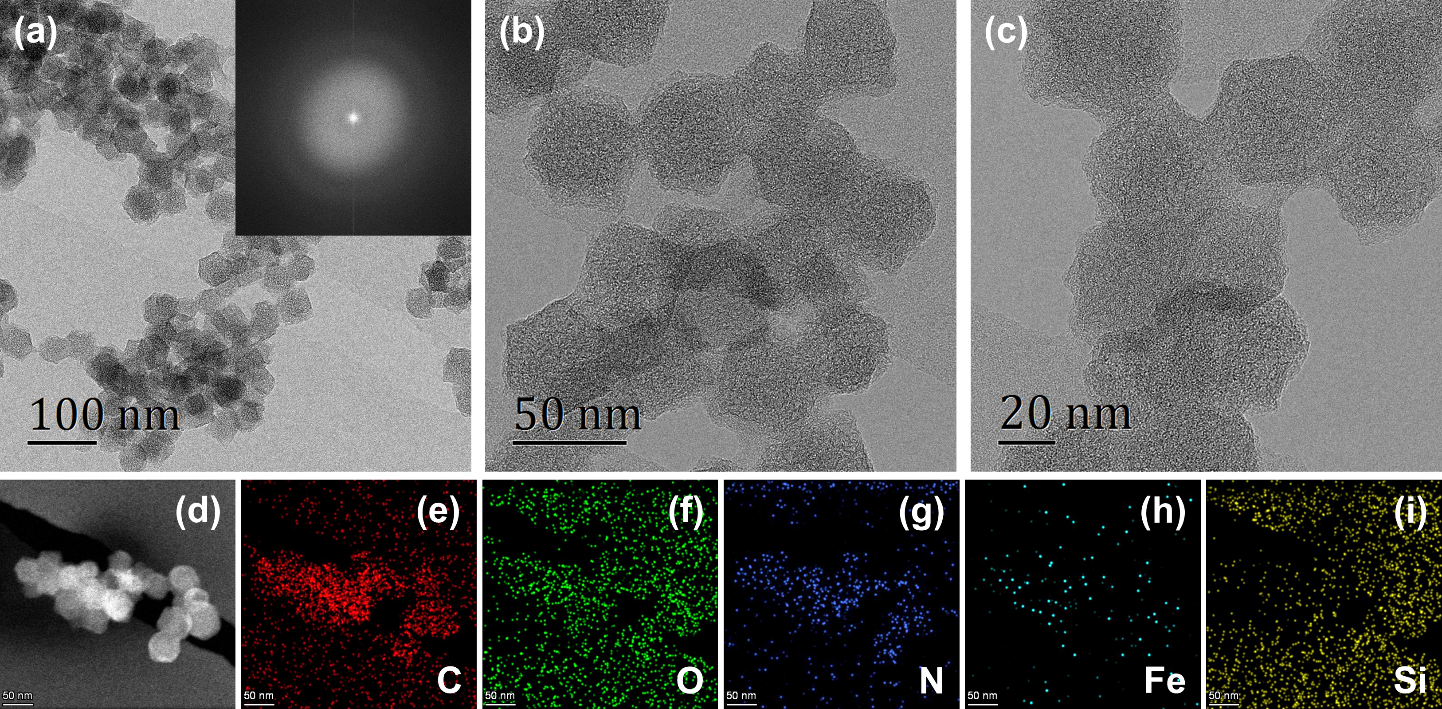


**Figure S36.** (a-c) TEM and (d-h) EDX mapping images of Fe-N-C-Si based GDE after electrolysis at 200 mA cm^–2^ for 4h. Inset in (a) is the corresponding SAED pattern.


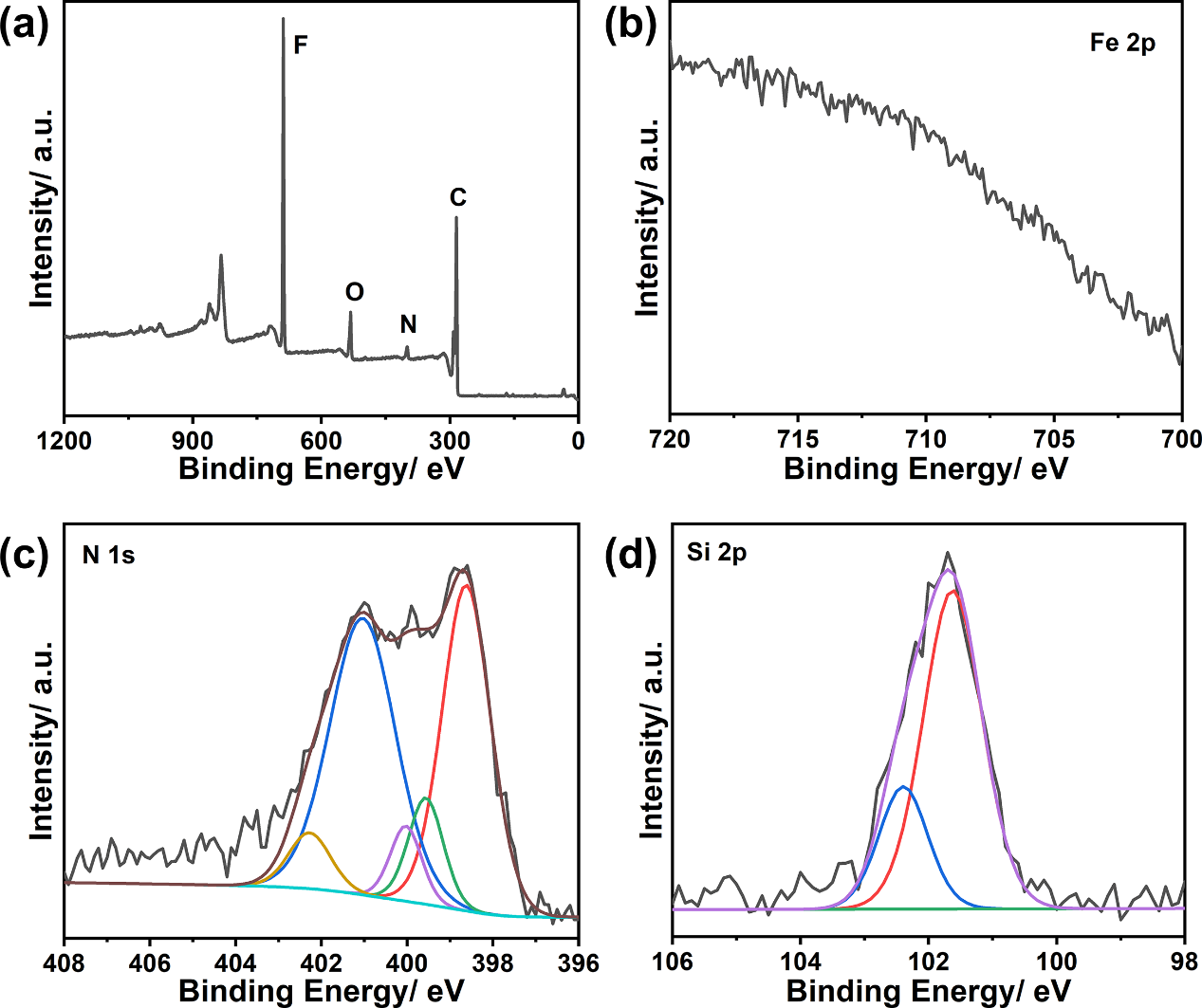


**Figure S37.** (a) Survey, (b) Fe 2p, (c) N 1s and (d) Si 2p XPS spectra of Fe-N-C-Si based GDE after electrolysis at 200 mA cm^–2^ for 4h.


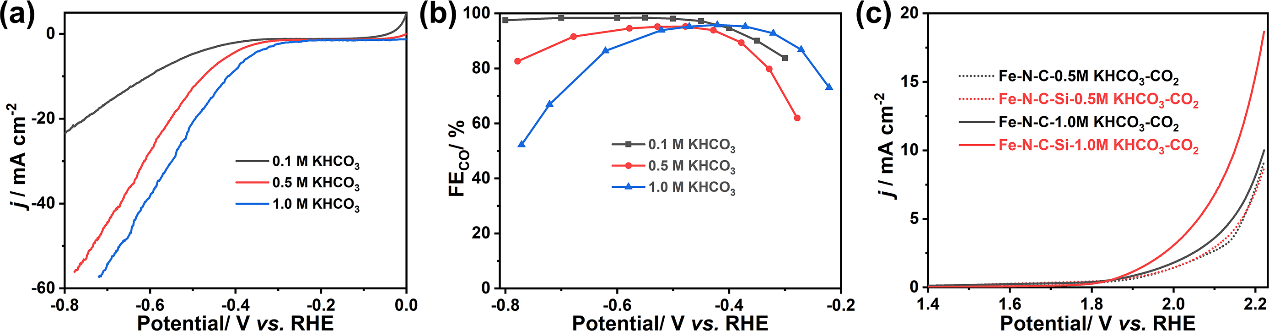


**Figure S38.** (a) LSV curves and (b) potential-dependent FE_CO_ of Fe-N-C-Si for CO_2_RR collected in CO_2_-saturated KHCO_3_ with difference concentrations. (c) OER performances of Fe-N-C and Fe-N-C-Si in CO_2_-saturated KHCO_3_ with difference concentrations.

As shown in Figure S38(a), the current density in CO_2_-saturated 1.0 m KHCO_3_ is much higher than that in CO_2_-saturated 0.5 m KHCO_3_ while not much sacrificing the values of FE_CO_ (Figure S38(b)). Moreover, Fe-N-C-Si shows much higher OER performance in CO_2_-saturated 1.0 m KHCO_3_ (Figure S38(c)). After comprehensive consideration, we chose CO_2_-saturated 1.0 m KHCO_3_ as the electrolyte for Zn-CO_2_ battery tests.


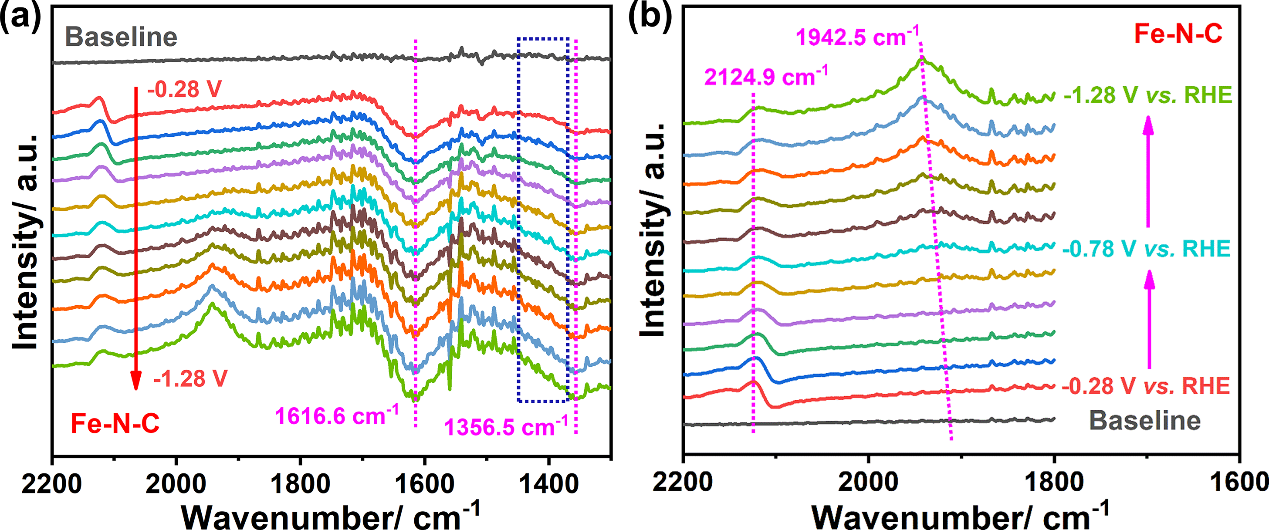


**Figure S39.** *In situ* ATR-IR spectra of Fe-N-C recorded while stepping the potentials in CO_2_-saturated 0.5 m KHCO_3_.





**Figure S40.** *In situ* ATR-IR spectra of Fe-N-C-Si recorded while stepping the potentials in CO_2_-saturated 0.5 m KHCO_3_.


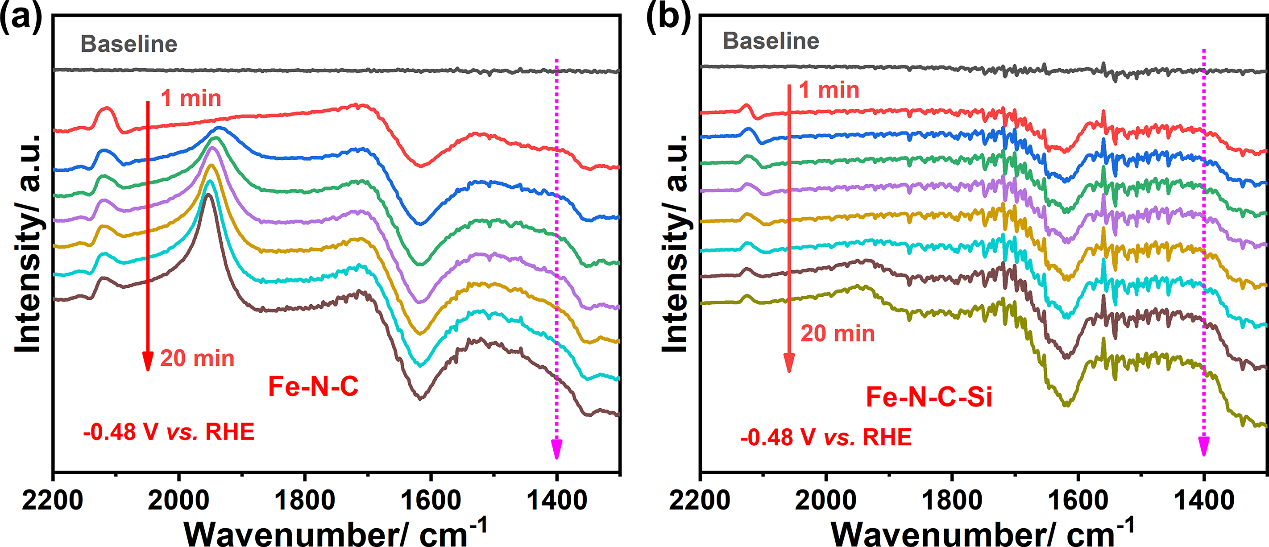


**Figure S41.** Real-time ATR-IR spectra of (a) Fe-N-C and (b) Fe-N-C-Si collected at –0.28 V in CO_2_-saturated 0.5 m KHCO_3_.





**Figure S42.** CO-TPD data of Fe-N-C and Fe-N-C-Si.

As shown in **Figure S42**, an obvious CO desorption peak centered at 351 ℃ was observed for Fe-N-C, much lower than that (315 ℃) for Fe-N-C-Si. The result demonstrates that a much weaker CO binding energy on Fe-N-C-Si surface than that of Fe-N-C, which, in turn, is beneficial to enhance the CO_2_RR performance [10, 11].


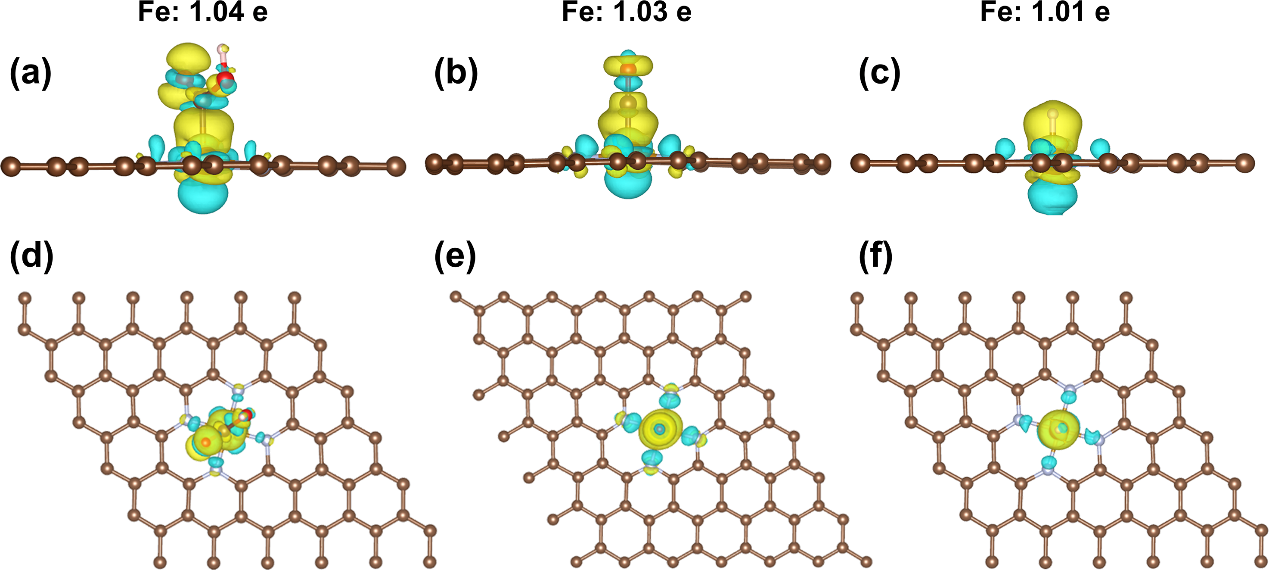


**Figure S43.** Differential charge densities of Fe-4N after adsorbing the (a, d) *COOH, (b, e) *CO, and (c, f) *H intermediates to the single Fe atom.


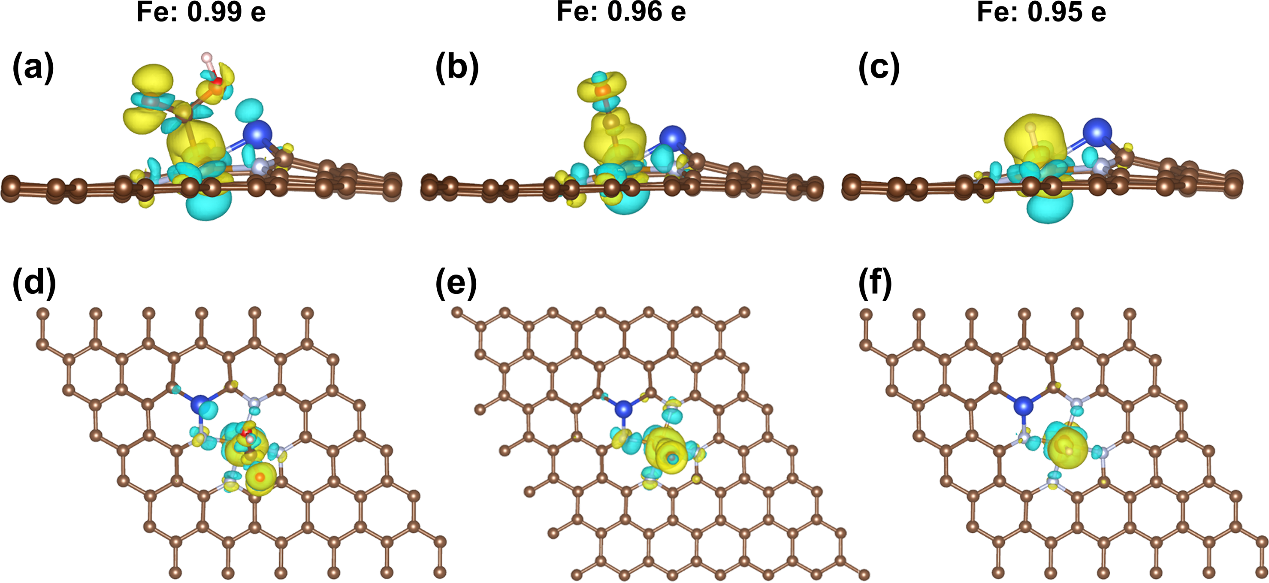


**Figure S44.** Differential charge densities of Fe-4N-Si-1 after adsorbing the (a, d) *COOH, (b, e) *CO, and (c, f) *H intermediates to the single Fe atom.


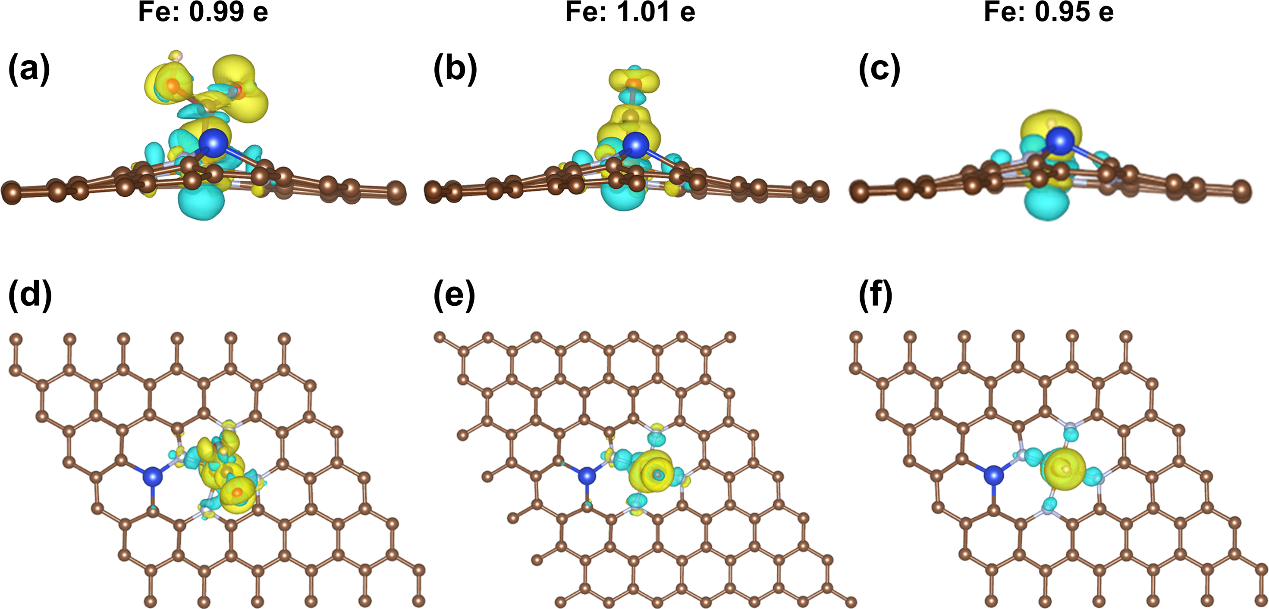


**Figure S45.** Differential charge densities of Fe-4N-Si-2 after adsorbing the (a, d) *COOH, (b, e) *CO, and (c, f) *H intermediates to the single Fe atom.


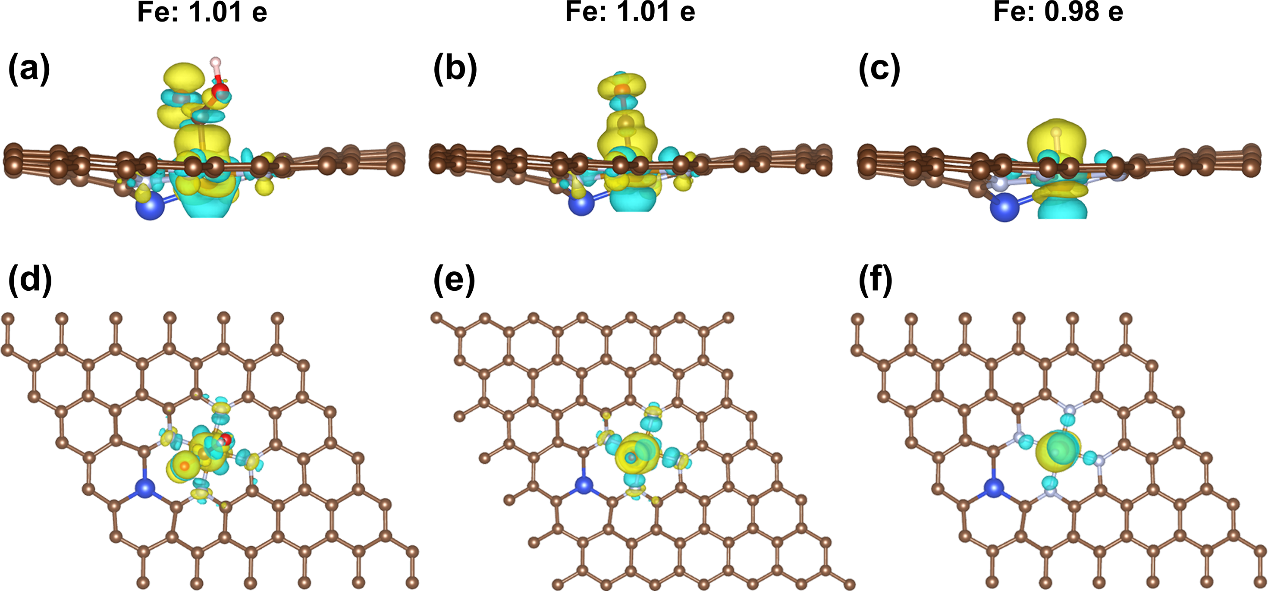


**Figure S46.** Differential charge densities of Fe-4N-Si-3 after adsorbing the (a, d) *COOH, (b, e) *CO, and (c, f) *H intermediates to the single Fe atom.


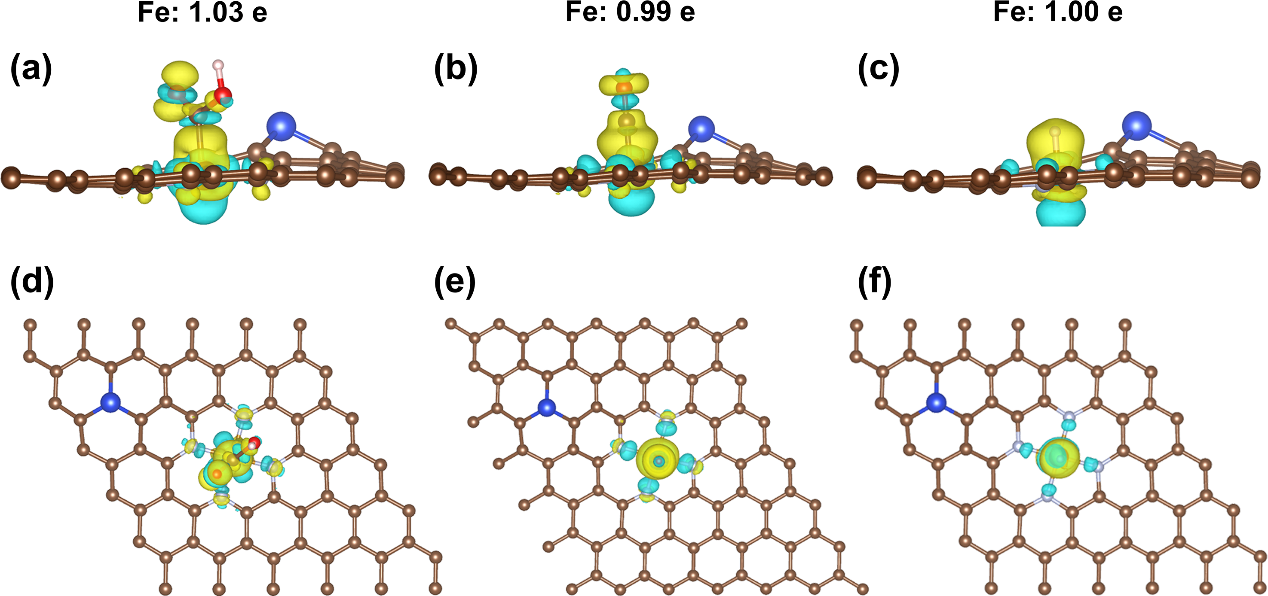


**Figure S47.** Differential charge densities of Fe-4N-Si-4 after adsorbing the (a, d) *COOH, (b, e) *CO, and (c, f) *H intermediates to the single Fe atom.


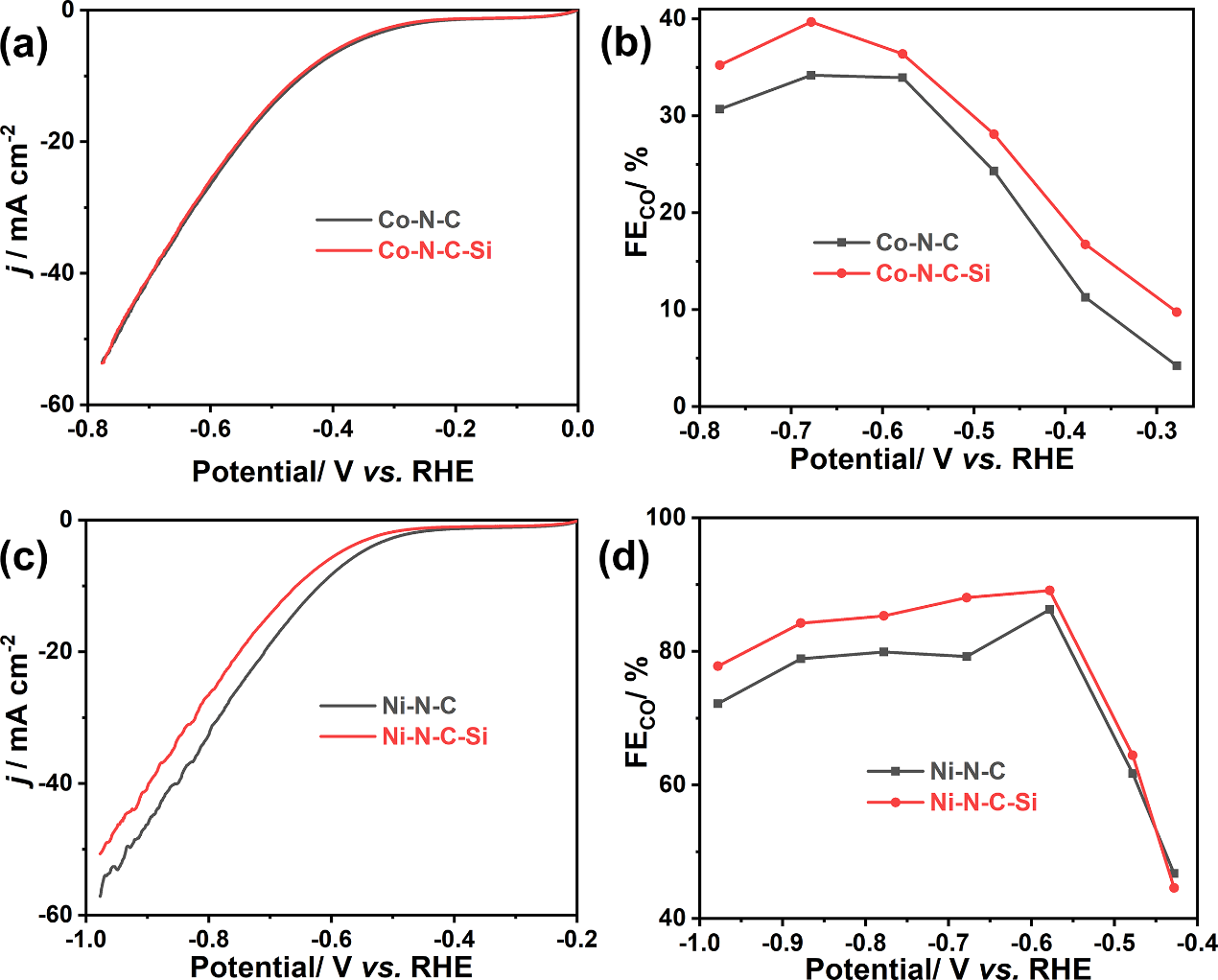


**Figure S48.** CO_2_RR performance of (a, b) Co-N-C and Co-N-C-Si and (c, d) Ni-N-C and Ni-N-C-Si measured in H-type cell.

**2. Supplementary Tables**

**Table S1.** BET surface areas, pore volumes and Fe contents (measured by ICP) of Fe-N-C-Si with different Si contents.

|  | **BET/ m^2^ g^-1^** | **Pore volume/cm^3^ g^-1^** | **Fe wt%-ICP** |
| --- | --- | --- | --- |
| **Fe-N-C** | **635.0** | **0.8191** | **0.42** |
| Fe-N-C-01Si | 845.29 | 0.9718 | 0.90 |
| Fe-N-C-02Si | 970.23 | 1.3566 | 1.24 |
| **Fe-N-C-Si** | **1172.4** | **2.0137** | **1.24** |
| Fe-N-C-04Si | 1217.9 | 2.2081 | 1.24 |
| Fe-N-C-05Si | 1075.2 | 1.9684 | 1.17 |

**Table S2.** The fitting parameters of Fe K-edge EXAFS data for Fe foil, Fe-N-C and Fe-N-C-Si.

| **Sample** | **Path** | **CN** | **R (Å)** | **σ^2^×10^3^ (Å^2^)** | **ΔE (eV)** | **R factor** |
| --- | --- | --- | --- | --- | --- | --- |
| Fe foil | Fe-Fe_1_ | 8 | 2.47 | 4.94 | 5.61 | 0.007 |
|  | Fe-Fe_2_ | 6 | 2.87 | 5.72 | 5.61 |  |
| Fe-N-C | Fe-N | 3.8±0.5 | 2.01 | 11.55 | –1.34 | 0.024 |
| Fe-N-C-Si | Fe-N | 4.1±0.4 | 2.01 | 11.94 | 0.16 | 0.010 |

S_0_^2^= 0.723 is determined by fitting EXAFS spectrum of Fe foil; CN is the coordination number; R is the interatomic distance (bond length from center atom to coordination atom); σ^2^ represents the Debye-Waller factor to evaluate thermal and static disorder; ΔE indicates edge-energy shift; R-factor indicates the accuracy of the fitting.

**Table S3**. Comparison of the electrocatalytic CO_2_RR performance in **H-type cell** over Fe-N-C-Si and other carbon-based single-atom electrocatalysts.

| Catalysts | Electrolyte | ^a^η_onset_  (mV) | ^b^*E*_app._  (V *vs.* RHE) | ^c^FE_CO_  (%) | ^d^*j_CO_*  (mA cm^−2^) | Ref. |
| --- | --- | --- | --- | --- | --- | --- |
| **Fe-N-C** | **0.5 m KHCO_3_** | **170** | **–0.48** | **94.18** | **6.13** | **This work** |
| **Fe-N-C-Si** |  | **170** | **–0.43** | **93.89** | **5.54** |  |
|  |  |  | **–0.48** | **95.22** | **9.91** |  |
|  |  |  | **–0.53** | **95.15** | **15.68** |  |
|  |  |  | **–0.58** | **94.55** | **22.57** |  |
| Fe-NS-C | 0.1 m KHCO_3_ | 140 | –0.58 | 98 | 7.1 | [12] |
|  | 0.5 m KHCO_3_ | ^e^n.m. | –0.56 | 93 | 12.1 |  |
| Fe-CNPs | 1.0 m KHCO_3_ | 270 | –0.58 | 98.8 | 6 | [13] |
| Fe-N-C | 0.5 m KHCO_3_ | 260 | –0.47 | 85 | 1.28 | [14] |
| Fe_0.5_d | 0.1 m NaHCO_3_ | 190 | –0.6 | 91 | 6.82 | [15] |
| Fe-NC-S | 0.5 m KHCO_3_ | 190 | –0.4 | 93 | 4 | [16] |
| Fe−N/CNF-2 | 0.1 m KHCO_3_ | 270 | –0.53 | 95 | 4.47 | [17] |
| Fe/ NG-750 | 0.1 m KHCO_3_ | 160 | –0.57 | 80 | 1.65 | [18] |
| Fe_3_C\|Fe_1_N_4_ | 0.5 m KHCO_3_ | 190 | –0.5 | 94.6 | 1 | [19] |
| DNG-SAFe | 0.1 m KHCO_3_ | 140 | –0.75 | 90 | 15.3 | [20] |
| Fe_1_NC/S_1_-1000 | 0.5 m KHCO_3_ | 190 | –0.5 | 96 | 5 | [21] |
| Fe^3+^–N–C | 0.5 m KHCO_3_ | 80 | –0.45 | 94 | ~16 | [22] |
| FeN_4_-O_1_ | 0.1 m NaHCO_3_ | n.m. | –0.83 | ~100 | 11.23 | [23] |
| A-Fe@_NG_-Li_1_K_3_ | 0.5 m KHCO_3_ | 140 | –0.45 | 95 | 10 | [24] |
| FeN_4_Cl/_NC_-7.5 | 0.5 m KHCO_3_ | 200 | –0.6 | 90.5 | 9.8 | [25] |
| H_2_–FeN_4_/C | 0.1 m NaHCO_3_ | 90 | –0.6 | 97 | 6.66 | [26] |
| Fe-SAC/NPC | 0.5 m KHCO_3_ | 90 | –0.43 | 97 | 5 | [27] |
|  |  |  | –0.53 | 98 | 11 |  |
| 200 nm FeNC | 0.1 m NaHCO_3_ | n.m. | –0.49 | 95.3 | 8.0 | [28] |
| Fe-N-C | 0.5 m KHCO_3_ | n.m. | –0.56 | 49.7 | ~3 | [29] |
| Fe-SA/BNC | 0.1 m KHCO_3_ | n.m. | –0.7 | 94 | ~7 | [30] |
| Fe-N_4_/CF-1,000 | 0.5 m KHCO_3_ | n.m. | –0.5 | 94.9 | 5 | [31] |
| FeN/CNT@GNR | 0.1 m KHCO_3_ | 190 | –0.76 | 98 | 10.6 | [32] |
|  | 0.5 m KHCO_3_ | n.m. | –0.76 | 96 | 22.7 |  |
| O-Fe-N-C | 0.5 m NaHCO_3_ | n.m. | –0.5 | 95 | 4.4 | [33] |
| C-AFC©ZIF-8 | 0.1 m KHCO_3_ | 220 | –0.63 | 91.6 | 4.1 | [34] |
|  | 0.5 m KHCO_3_ | n.m. | –0.53 | 89.6 | 4.8 |  |
| Co-N_5_/HNPCSs | 0.2 m NaHCO_3_ | n.m. | –0.79 | 99.3 | 10.13 | [35] |
| CoN_4_-CB | 0.5 m KHCO_3_ | 250 | –0.66 | 98.7 | 21.2 | [36] |
| Ni SAs/N-C | 0.5 m KHCO_3_ | 460 | –1.0 | 70.3 | 7.36 | [37] |
| Ni-N-C | 0.1 m KHCO_3_ | 490 | –0.75 | 97 | 7.5 | [38] |
| 3D SANi-G | 0.5 m KHCO_3_ | 290 | –0.8 | 98.9 | 42.9 | [39] |
| Ni-NCB | 0.5 m KHCO_3_ | 290 | –0.68 | 99 | 6.8 | [40] |
| Cu–N–C | 0.5 m KHCO_3_ | 260 | –0.67 | 98 | 3.6 | [41] |
| ZnN_x_/C | 0.5 m KHCO_3_ | 24 | –0.43 | 95 | 4.56 | [42] |
| Mn-_C3N4_/CNT | 0.5 m KHCO_3_ | n.m. | –0.55 | 98.8 | 14.0 | [43] |
| (Cl, N)-Mn/G | 0.5 m KHCO_3_ | n.m. | –0.6 | 97 | 9.2 | [44] |
| Bi SAs/NC | 0.1 m NaHCO_3_ | n.m. | –0.5 | 97 | 3.9 | [45] |
| Mg-C_3_N_4_ | 0.5 m KHCO_3_ | n.m. | –0.978 | 92 | 14 | [46] |
| Co-N-Ni/NPCNSs | 0.1 m KHCO_3_ | 190 | –0.48 | 96.4 | 3.2 | [47] |
| Fe_1_−Ni_1_−N−C | 0.5 m KHCO_3_ | 190 | –0.5 | 96.2 | 2.4 | [48] |
| 4H-Au nanoribbon | 0.1 m KHCO_3_ | 190 | –0.7 | 90 | 7.7 | [49] |
| Pd@Pd_3_Au_7_ NCs | 0.1 m KHCO_3_ | n.m. | –0.5 | 94 | 2.43 | [50] |
| Ag-D | 0.1 m KHCO_3_ | n.m. | –0.81 | ~100 | 2.73 | [51] |
| 5.1 nm Pd | 0.1 m NaHCO_3_ | 90 | –0.5 | 90 | 3 | [52] |

**^a^η_onset_**: Onset overpotential; **^b^*E*_app._**: The applied potential to get the maximum FE_CO_; **^c^FE_CO_**: The maximum FE_CO_ values; **^d^*j_CO_***: The *j_CO_* at the maximum FE_CO_; **^e^n.m.**: Not mentioned.

**Note:** If the η_onset,_ FE_CO_ and *j_CO_* values are not specifically stated, they are derived from graphical results or calculated with the available information.

**Table S4**. Comparison of the electrocatalytic CO_2_RR performance in **flow cell** over Fe-based electrocatalysts.

| Catalysts | Electrolyte | *j_CO_*  (mA cm^−2^) | *E*_app._  (V *vs.* RHE) | FE_CO_  (%) | Ref. |
| --- | --- | --- | --- | --- | --- |
| **Fe-N-C** | **1.0 m KOH** | **98.32** | **–0.285** | **98.32** | **This work** |
| **Fe-N-C-Si** |  | **49.72** | **–0.181** | **99.44** |  |
|  |  | **99.38** | **–0.242** | **99.38** |  |
|  |  | **148.52** | **–0.295** | **99.01** |  |
|  |  | **195.92** | **–0.358** | **97.96** |  |
|  |  | **237.85** | **–0.412** | **95.14** |  |
| A-Fe@NG-Li_1_ K_3_ | 1.0 m KOH | 144.7 | –0.5 | 91.3 | [24] |
|  |  | 187.2 | –0.65 | 84 |  |
| Fe^3+^–N–C | 0.5 m KHCO_3_ | 94 | –0.45 | 90 | [22] |
| Fe–N–C | 0.5 m KOH | 24.6 | −0.31 | 90 | [53] |
| SM-Fe | 1.0 m KOH | 65 | −0.35 | 89 | [54] |
| Fe-N-C | 1.0 m KOH | 112.5 | −0.3 | 90 | [11] |
| Fe_3_C\|Fe_1_N_4_ | 1.0 m KHCO_3_ | 8.8 | −0.75 | 88 | [19] |
| Fe-_SAC_ (Pc) | 1.0 m KHCO_3_ | 50.1 | −0.72 | 50.1 | [55] |
| Ni-SA/NC | 1.0 m KOH | 213.2 | −0.66 | 96.9 | [56] |
| CNNi-700 | 1.0 m KOH | 223 | −0.93 | 97 | [57] |

**Note:** If the FE_CO_ and *j_CO_* values are not specifically stated, they are derived from graphical results or calculated with the available information.

**Table S5.** Comparison of the performance of recently reported Zn-CO_2_ batteries.

| Cathode | Electrolyte  Catholyte // Anolyte | Products | FE / *j*  (% / mA cm^−2^) | Powder density | Ref. |
| --- | --- | --- | --- | --- | --- |
| **Fe-N-C** | **1.0 m KHCO_3_ //**  **6.0 m KOH + 0.2 m Zn(AC)_2_** | **CO** | **90.1% / 5 mA cm^−2^** | **2.13 mW cm^−2^ at ~8.0 mA cm^−2^** | **This work** |
| **Fe-N-C-Si** |  | **CO** | **94.5% / 5 mA cm^−2^** | **2.44 mW cm^−2^ at ~8.2 mA cm^−2^** |  |
| CoPc@DNHCS-8 | 0.8 m KHCO_3_ //  0.8 m KOH + 0.02 m Zn(AC)_2_ | CO | 94% / 3 mA cm^−2^ | 1.02 mW cm^−2^ at ~3.0 mA cm^−2^ | [58] |
| FeN_4_Cl/NC-7.5 | 0.8 m KHCO_3_ //  0.8 m KOH + 0.02 m Zn(AC)_2_ | CO | n.m. | 0.545 mW cm^−2^ | [25] |
| Fe_1_NC/S_1_-1000 | 0.8 m KHCO_3_ //  0.8 m KOH + 0.02 m Zn(AC)_2_ | CO | n.m. | 0.526 mW cm^−2^ at ~2.5 mA cm^−2^ | [21] |
| Fe-SA/BNC | 0.8 m KHCO_3_ //  0.8 m KOH + 0.02 m Zn(AC)_2_ | CO | 91.4% / 1 mA cm^−2^ | 1.18 mW cm^−2^ at ~4.0 mA cm^−2^ | [30] |
| CBNGC-2 | 0.8 m KHCO_3_ //  0.8 m KOH + 0.02 m Zn(AC)_2_ | CO | 80.4% / 2.56 mA cm^−2^ | 0.51 mW cm^−2^ at 2.14 mA cm^−2^ | [59] |
| Fe_3_C\|Fe_1_N_4_ | 0.8 m KHCO_3_ //  0.8 m KOH + 0.02 m Zn(AC)_2_ | CO | n.m. | 0.33 mW cm^−2^ | [19] |
| Ni−N_3_−NCNFs | 0.5 m KHCO_3_ //  6 m KOH + 0.2 m Zn(AC)_2_ | CO | 96% / 3 mA cm^−2^ | 1.05 mW cm^−2^ | [60] |
| Cu–N_2_/GN | 0.5 m KHCO_3_ //  6 m KOH + 0.2 m Zn(AC)_2_ | CO | 64% / 1.4 mA cm^−2^ | 0.62 mW cm^−2^ | [61] |
| DNG-SAFe | 1.0 m KHCO_3_ //  6 m KOH + 0.2 m Zn(AC)_2_ | CO | 86.5% / 5 mA cm^−2^ | 0.925 mW cm^−2^ at 4.3 mA cm^−2^ | [20] |
| Ir@Au | 0.8 m KHCO_3_ //  0.8 m KOH + 0.02 m Zn(AC)_2_ | CO | 90% / 8.3 mA cm^−2^ | n.m. | [62] |
| Ni-N_3_-C | 0.8 m KHCO_3_ //  0.8 m KOH + 0.02 m Zn(AC)_2_ | CO | 93% / 2 mA | n.m. | [63] |
| Fe-SA/BNC | 0.8 m KHCO_3_ //  0.8 m KOH + 0.02 m Zn(AC)_2_ | CO | 91.4% / 1.0 mA cm^−2^ | 1.18 mW cm^−2^ at 4 mA cm^−2^ | [30] |
| Fe_1_−Ni_1_−N−C | 0.8 m KHCO_3_ //  0.8 m KOH + 0.02 m Zn(AC)_2_ | CO | 93.4% / 1 mA | n.m. | [48] |
| SAs–Ni–N–C | 0.5 m KHCO_3_ //  6 m KOH + 0.2 m Zn(AC)_2_ | CO | 93.3% / 2.0 mA cm^−2^ | 1.4 mW cm^−2^ at 5.3 mA cm^−2^ | [64] |
| NiFe-DASC | 2 m KCl //  2 m KOH + 0.02 m Zn(AC)_2_ | CO | 90.6% / 5 mA cm^−2^ | 1.36 mW cm^−2^ at 8.5 mA cm^−2^ | [65] |
| NOMC | 0.8 m KHCO_3_ //  6 m KOH + 0.2 m Zn(AC)_2_ | CO | 76% / 1.0 mA cm^−2^ | 0.71 mW cm^−2^ at 3 mA cm^−2^ | [66] |
| VO-rich N-SnO_2_ | 0.5 m KHCO_3_ //  6 m KOH + 0.2 m Zn(AC)_2_ | Formate | 74% / 6.0 mA cm^−2^ | 3.67 mW cm^−2^ at 15.5 mA cm^−2^ | [67] |
| Bi-D | 2 m KHCO_3_ //  2 m KOH + 0.02 m Zn(AC)_2_ | Formate | n.m. | 1.16 mW cm^−2^ | [68] |

**Table S6**. Bader charges of Fe atom in five single-atom Fe configurations after *COOH, *CO, and *H intermediates were adsorbed.

| **Configurations** | **Atom** | ***COOH (e)** | ***CO (e)** | ***H (e)** |
| --- | --- | --- | --- | --- |
| Fe-4N | Fe | 1.04 | 1.03 | 1.01 |
| Fe-4N-Si-1 | Fe | 0.99 | 0.96 | 0.95 |
| Fe-4N-Si-2 | Fe | 0.99 | 1.01 | 0.95 |
| Fe-4N-Si-3 | Fe | 1.01 | 1.01 | 0.98 |
| Fe-4N-Si-4 | Fe | 1.03 | 0.99 | 1.00 |

**Table S7.** The distances between Fe and C atoms after the adsorption of *CO intermediate and the corresponding -COHP values in five Fe single-atom configurations.

| **Configurations** | **Fe-C distance/ Å** | **-COHP** |
| --- | --- | --- |
| Fe-4N | 1.7020 | 5.22445 |
| **Fe-4N-Si-1** | **1.7095** | **5.03002** |
| Fe-4N-Si-2 | 1.7071 | 5.14098 |
| Fe-4N-Si-3 | 1.7071 | 5.14087 |
| Fe-4N-Si-4 | 1.7071 | 5.14089 |

References

[1] Deng, W.; Zhang, L.; Li, L.; et al., “Crucial Role of Surface Hydroxyls on the Activity and Stability in Electrochemical CO_2_ Reduction,” *Journal of The American Chemical Society*, vol. 141, no. 7, pp. 2911–2915, 2019.

[2] Xiong, L.; Zhang, X.; Yuan, H.; et al., “Breaking the Linear Scaling Relationship by Compositional and Structural Crafting of Ternary Cu–Au/Ag Nanoframes for Electrocatalytic Ethylene Production,” *Angewandte Chemie International Edition*, vol. 60, no. 5, pp. 2508–2518, 2021.

[3] Kresse, G.; Hafner, J., “Ab initio molecular dynamics for liquid metals,” *Physical Review B*, vol. 47, no. 1, pp. 558–561, 1993.

[4] Kresse, G.; Furthmüller, J., “Efficiency of ab-initio total energy calculations for metals and semiconductors using a plane-wave basis set,” *Computational Materials Science*, vol. 6, no. 1, pp. 15–50, 1996.

[5] Blöchl, P. E., “Projector augmented-wave method,” *Physical Review B*, vol. 50, no. 24, pp. 17953–17979, 1994.

[6] Perdew, J. P.; Burke, K.; Ernzerhof, M., “Generalized Gradient Approximation Made Simple,” *Physical Review Letters*, vol. 77, no. 18, pp. 3865–3868, 1996.

[7] Cao, C.; Wen, Z., “Cu nanoparticles decorating rGO nanohybrids as electrocatalyst toward CO_2_ reduction,” *Journal of CO_2_ Utilization*, vol. 22, pp. 231–237, 2017.

[8] ACS Earth and Space ChemistryAnantharaj, S.; Noda, S.; Driess, M.; et al., “The Pitfalls of Using Potentiodynamic Polarization Curves for Tafel Analysis in Electrocatalytic Water Splitting,” *ACS Energy Letters*, vol. 6, no. 4, pp. 1607–1611, 2021.

[9] Dunwell, M.; Lu, Q.; Heyes, J. M.; et al., “The Central Role of Bicarbonate in the Electrochemical Reduction of Carbon Dioxide on Gold,” *Journal of The American Chemical Society*, vol. 139, no. 10, pp. 3774–3783, 2017.

[10] Xia, R.; Zhang, S.; Ma, X.; et al., “Surface-functionalized palladium catalysts for electrochemical CO_2_ reduction,” *Journal of Materials Chemistry A*, vol. 8, no. 31, pp. 15884–15890, 2020.

[11] Lin, L.; Li, H.; Wang, Y.; et al., “Temperature-Dependent CO_2_ Electroreduction over Fe-N-C and Ni-N-C Single-Atom Catalysts,” *Angewandte Chemie International Edition*, vol. 60, no. 51, pp. 26582–26586, 2021.

[12] Pan, F.; Li, B.; Sarnello, E.; et al., “Boosting CO_2_ reduction on Fe-N-C with sulfur incorporation: Synergistic electronic and structural engineering,” *Nano Energy*, vol. 68, p. 104384, 2020.

[13] Hu, C.; Bai, S.; Gao, L.; et al., “Porosity-Induced High Selectivity for CO_2_ Electroreduction to CO on Fe-Doped ZIF-Derived Carbon Catalysts,” *ACS Catalysis*, vol. 9, no.12, pp. 11579–11588, 2019.

[14] Hu, X.-M.; Hval, H. H.; Bjerglund, E. T.; et al., “Selective CO_2_ Reduction to CO in Water using Earth-Abundant Metal and Nitrogen-Doped Carbon Electrocatalysts,” *ACS Catalysis*, vol. 8, no.7, pp. 6255–6264, 2018.

[15] Huan, T. N.; Ranjbar, N.; Rousse, G.; et al., “Electrochemical Reduction of CO_2_ Catalyzed by Fe-N-C Materials: A Structure–Selectivity Study,” *ACS Catalysis*, vol. 7, no. 3, pp. 1520–1525, 2017.

[16] Li, X.; Zeng, Y.; Tung, C.-W.; et al., “Unveiling the In Situ Generation of a Monovalent Fe(I) Site in the Single-Fe-Atom Catalyst for Electrochemical CO_2_ Reduction,” *ACS Catalysis*, vol. 11, no. 12, pp. 7292–7301, 2021.

[17] Cheng, Q.; Mao, K.; Ma, L.; et al., “Encapsulation of Iron Nitride by Fe–N–C Shell Enabling Highly Efficient Electroreduction of CO_2_ to CO,” *ACS Energy Letters*, vol. 3, no. 5, pp. 1205–1211, 2018.

[18] Zhang, C.; Yang, S.; Wu, J.; et al., “Electrochemical CO_2_ Reduction with Atomic Iron‐Dispersed on Nitrogen‐Doped Graphene,” *Advanced Energy Materials*, vol. 8, no. 19, p. 1703487, 2018.

[19] Chen, J.; Wang, T.; Wang, X.; et al., “Promoting Electrochemical CO_2_ Reduction via Boosting Activation of Adsorbed Intermediates on Iron Single-Atom Catalyst,” *Advanced Functional Materials*, vol. 32, no. 21, p. 2110174, 2022.

[20] Ni, W.; Liu, Z.; Zhang, Y.; et al., “Electroreduction of Carbon Dioxide Driven by the Intrinsic Defects in the Carbon Plane of a Single Fe–N_4_ Site,” *Advanced Materials*, vol. 33, no. 1, p. 2003238, 2020.

[21] Wang, T.; Sang, X.; Zheng, W.; et al., “Gas Diffusion Strategy for Inserting Atomic Iron Sites into Graphitized Carbon Supports for Unusually High-Efficient CO_2_Electroreduction and High-Performance Zn–CO_2_ Batteries,” *Advanced Materials*, vol. 32, no. 29, p. 2002430, 2020.

[22] Gu, J.; Hsu, C.-S.; Bai, L.; et al., “Atomically dispersed Fe^3+^ sites catalyze efficient CO_2_ electroreduction to CO,” *Science*, vol. 364, no. 6445, pp. 1091–1094, 2019.

[23] Chen, Z.; Huang, A.; Yu, K.; et al., “Fe_1_N_4_–O_1_ site with axial Fe–O coordination for highly selective CO_2_ reduction over a wide potential range,” *Energy & Environmental Science*, vol. 14, no. 6, pp. 3430–3437, 2021.

[24] Liu, W.; Wang, K.; Gong, L.; et al., “Edge-located Fe-N_4_ sites on porous Graphene-like nanosheets for boosting CO_2_ electroreduction,” *Chemical Engineering Journal*, vol. 431, p. 134269, 2022.

[25] Li, Z.; Wu, R.; Xiao, S.; et al., “Axial chlorine coordinated iron-nitrogen-carbon single-atom catalysts for efficient electrochemical CO_2_ reduction,” *Chemical Engineering Journal*, vol. 430, p. 132882, 2022.

[26] Liu, C.; Wu, Y.; Sun, K.; et al., “Constructing FeN_4_/graphitic nitrogen atomic interface for high-efficiency electrochemical CO_2_ reduction over a broad potential window,” *Chem*, vol. 7, no. 5, pp. 1297–1307, 2021.

[27] Sun, X.; Tuo, Y.; Ye, C.; et al., “Phosphorus Induced Electron Localization of Single Iron Sites for Boosted CO_2_ Electroreduction Reaction,” *Angewandte Chemie International Edition*, vol. 60, no. 44, pp. 23614–23618, 2021.

[28] Mohd Adli, N.; Shan, W.; Hwang, S.; et al., “Engineering Atomically Dispersed FeN_4_ Active Sites for CO_2_ Electroreduction,” *Angewandte Chemie International Edition*, vol. 60, no. 2, pp. 1022–1032, 2020.

[29] Wang, C.; Hu, X.; Hu, X.; et al., “Typical transition metal single-atom catalysts with a metal-pyridine N structure for efficient CO_2_ electroreduction,” *Applied Catalysis B: Environmental*, vol. 296, p. 120331, 2021.

[30] Liu, S.; Jin, M.; Sun, J.; et al., “Coordination environment engineering to boost electrocatalytic CO_2_ reduction performance by introducing boron into single-Fe-atomic catalyst,” *Chemical Engineering Journal*, vol. 437, p. 135294, 2022.

[31] Zhang, Z.; Ma, C.; Tu, Y.; et al., “Multiscale carbon foam confining single iron atoms for efficient electrocatalytic CO_2_ reduction to CO,” *Nano Research*, vol. 12, no. 9, pp. 2313–2317, 2019.

[32] Pan, F.; Li, B.; Sarnello, E.; et al., “Atomically Dispersed Iron–Nitrogen Sites on Hierarchically Mesoporous Carbon Nanotube and Graphene Nanoribbon Networks for CO_2_ Reduction,” *ACS nano*, vol. 14, no. 5, pp. 5506–5516, 2020.

[33] Zhang, T.; Han, X.; Liu, H.; et al., “Site-Specific Axial Oxygen Coordinated FeN_4_ Active Sites for Highly Selective Electroreduction of Carbon Dioxide,” *Advanced Functional Materials*, vol. 32, no. 18, pp. 2111446, 2022.

[34] Ye, Y.; Cai, F.; Li, H.; et al., “Surface functionalization of ZIF-8 with ammonium ferric citrate toward high exposure of Fe-N active sites for efficient oxygen and carbon dioxide electroreduction,” *Nano Energy*, vol. 38, p. 281–289, 2017.

[35] Pan, Y.; Lin, R.; Chen, Y.; et al., “Design of Single-Atom Co–N_5_ Catalytic Site: A Robust Electrocatalyst for CO_2_ Reduction with Nearly 100% CO Selectivity and Remarkable Stability,” *Journal of The American Chemical Society*, vol. 140, no. 12, pp. 4218–4221, 2018.

[36] Wang, C.; Ren, H.; Wang, Z.; et al., “A promising single-atom Co-N-C catalyst for efficient CO2 electroreduction and high-current solar conversion of CO_2_ to CO,” *Applied Catalysis B: Environmental*, vol. 304, no., pp. 120958, 2022.

[37] Zhao, C.; Dai, X.; Yao, T.; et al., “Ionic Exchange of Metal–Organic Frameworks to Access Single Nickel Sites for Efficient Electroreduction of CO_2_,” *Journal of The American Chemical Society*, vol. 139, no. 24, pp. 8078–8081, 2017.

[38] Pan, F.; Zhang, H.; Liu, Z.; et al., “Atomic-level active sites of efficient imidazolate framework-derived nickel catalysts for CO_2_ reduction,” *Journal of Materials Chemistry A*, vol. 7, no. 46, pp. 26231–26237, 2019.

[39] Zhai, P.; Gu, X.; Wei, Y.; et al., “Enhanced mass transfer in three-dimensional single-atom nickel catalyst with open-pore structure for highly efficient CO_2_ electrolysis,” *Journal of Energy Chemistry*, vol. 62, p. 43–50, 2021.

[40] Zheng, T.; Jiang, K.; Ta, N.; et al., “Large-Scale and Highly Selective CO_2_ Electrocatalytic Reduction on Nickel Single-Atom Catalyst,” *Joule*, vol. 3, no. 1, pp. 265–278, 2019.

[41] Chen, S.; Li, Y.; Bu, Z.; et al., “Boosting CO_2_-to-CO conversion on a robust single-atom copper decorated carbon catalyst by enhancing intermediate binding strength,” *Journal of Materials Chemistry A*, vol. 9, no. 3, pp. 1705–1712, 2021.

[42] Yang, F.; Song, P.; Liu, X.; et al., “Highly Efficient CO_2_ Electroreduction on ZnN_4_-based Single-Atom Catalyst,” *Angewandte Chemie International Edition*, vol. 57, no. 38, pp. 12303–12307, 2018.

[43] Feng, J.; Gao, H.; Zheng, L.; et al., “A Mn-N_3_ single-atom catalyst embedded in graphitic carbon nitride for efficient CO_2_ electroreduction,” *Nature Communications*, vol. 11, no. 1, p. 4341, 2020.

[44] Zhang, B.; Zhang, J.; Shi, J.; et al., “Manganese acting as a high-performance heterogeneous electrocatalyst in carbon dioxide reduction,” *Nature Communications*, vol. 10, no. 1, p. 2980, 2019.

[45] Zhang, E.; Wang, T.; Yu, K.; et al., “Bismuth Single Atoms Resulting from Transformation of Metal–Organic Frameworks and Their Use as Electrocatalysts for CO_2_ Reduction,” *Journal of The American Chemical Society*, vol. 141, no. 42, pp. 16569–16573, 2019.

[46] Wang, Q.; Liu, K.; Fu, J.; et al., “Atomically Dispersed s-Block Magnesium Sites for Electroreduction of CO_2_ to CO,” *Angewandte Chemie International Edition*, vol. 60, no. 48, pp. 25241–25245, 2021.

[47] Pei, J.; Wang, T.; Sui, R.; et al., “N-bridged Co-N-Ni: new bimetallic sites for promoting electrochemical CO_2_ reduction,” *Energy & Environmental Science*, vol. 14, no. 5, pp. 3019–3028, 2021.

[48] Jiao, L.; Zhu, J.; Zhang, Y.; et al., “Non-Bonding Interaction of Neighboring Fe and Ni Single-Atom Pairs on MOF-Derived N-Doped Carbon for Enhanced CO_2_ Electroreduction,” *Journal of The American Chemical Society*, vol. 143, no. 46, pp. 19417–19424, 2021.

[49] Wang, Y.; Li, C.; Fan, Z.; et al., “Undercoordinated Active Sites on 4H Gold Nanostructures for CO_2_ Reduction,” *Nano Letters*, vol. 20, no. 11, pp. 8074–8080, 2020.

[50] Yuan, X.; Zhang, L.; Li, L.; et al., “Ultrathin Pd–Au Shells with Controllable Alloying Degree on Pd Nanocubes toward Carbon Dioxide Reduction,” *Journal of The American Chemical Society*, vol. 141, no. 12, pp. 4791–4794, 2019.

[51] Wu, X.; Guo, Y.; Sun, Z.; et al., “Fast operando spectroscopy tracking in situ generation of rich defects in silver nanocrystals for highly selective electrochemical CO_2_ reduction,” *Nature Communications*, vol. 12, no. 1, pp. 660, 2021.

[52] Zhu, W.; Zhang, L.; Yang, P.; et al., “Low-Coordinated Edge Sites on Ultrathin Palladium Nanosheets Boost Carbon Dioxide Electroreduction Performance,” *Angewandte Chemie International Edition*, vol. 57, no. 36, pp. 11544–11548, 2018.

[53] Lin, L.; Li, H.; Yan, C.; et al., “Synergistic Catalysis over Iron-Nitrogen Sites Anchored with Cobalt Phthalocyanine for Efficient CO_2_ Electroreduction,” *Advanced Materials*, vol. 31, no. 41, p. 1903470, 2019.

[54] Yang, H.; Wang, X.; Wang, S.; et al., “Double boosting single atom Fe–N_4_ sites for high efficiency O_2_ and CO_2_ electroreduction,” *Carbon*, vol. 182, p. 109–116, 2021.

[55] Wang, Y.; Jiang, Z.; Zhang, X.; et al., “Metal Phthalocyanine-Derived Single-Atom Catalysts for Selective CO_2_ Electroreduction under High Current Densities,” *ACS applied materials & interfaces*, vol. 12, no. 30, pp. 33795–33802, 2020.

[56] Wang, X.; Ding, S.; Yue, T.; et al., “Universal domino reaction strategy for mass production of single-atom metal-nitrogen catalysts for boosting CO_2_ electroreduction,” *Nano Energy*, vol. 82, p. 105689, 2021.

[57] Liu, W.; Wei, S.; Bai, P.; et al., “Robust coal matrix intensifies electron/substrate interaction of nickel-nitrogen (Ni-N) active sites for efficient CO_2_ electroreduction at industrial current density,” *Applied Catalysis B: Environmental*, vol. 299, p. 120661, 2021.

[58] Gong, S.; Wang, W.; Zhang, C.; et al., “Tuning the Metal Electronic Structure of Anchored Cobalt Phthalocyanine via Dual-Regulator for Efficient CO_2_ Electroreduction and Zn–CO_2_ Batteries,” *Advanced Functional Materials*, vol. 32, no. 17, p. 2110649, 2022.

[59] Hao, X.; An, X.; Patil, A. M.; et al., “Biomass-Derived N-Doped Carbon for Efficient Electrocatalytic CO_2_ Reduction to CO and Zn–CO_2_ Batteries,” *ACS applied materials & interfaces*, vol. 13, no. 3, pp. 3738–3747, 2021.

[60] Zheng, W.; Wang, Y.; Shuai, L.; et al., “Highly Boosted Reaction Kinetics in Carbon Dioxide Electroreduction by Surface-Introduced Electronegative Dopants,” *Advanced Functional Materials*, vol. 31, no. 15, p. 2008146, 2021.

[61] Zheng, W.; Yang, J.; Chen, H.; et al., “Atomically Defined Undercoordinated Active Sites for Highly Efficient CO_2_ Electroreduction,” *Advanced Functional Materials*, vol. 30, no. 4, p. 1907658, 2019.

[62] Wang, X.; Xie, J.; Ghausi, M. A.; et al., “Rechargeable Zn–CO_2_ Electrochemical Cells Mimicking Two-Step Photosynthesis,” *Advanced Materials*, vol. 31, no. 17, p. 1807807, 2019.

[63] Zhang, Y.; Jiao, L.; Yang, W.; et al., “Rational Fabrication of Low-Coordinate Single-Atom Ni Electrocatalysts by MOFs for Highly Selective CO_2_ Reduction,” *Angewandte Chemie International Edition*, vol. 60, no. 14, pp. 7607–7611, 2021.

[64] Zheng, W.; Chen, F.; Zeng, Q.; et al., “A Universal Principle to Accurately Synthesize Atomically Dispersed Metal–N_4_ Sites for CO_2_ Electroreduction,” *Nano-Micro Letters*, vol. 12, no. 1, p. 108, 2020.

[65] Zeng, Z.; Gan, L. Y.; Bin Yang, H.; et al., “Orbital coupling of hetero-diatomic nickel-iron site for bifunctional electrocatalysis of CO_2_ reduction and oxygen evolution,” *Nature Communications*, vol. 12, no. 1, p. 4088, 2021.

[66] Gao, S.; Liu, Y.; Xie, Z.; et al., “Metal-Free Bifunctional Ordered Mesoporous Carbon for Reversible Zn-CO_2_ Batteries,” *Small Methods*, vol. 5, no. 4, p. 2001039, 2021.

[67] Li, Z.; Cao, A.; Zheng, Q.; et al., “Elucidation of the Synergistic Effect of Dopants and Vacancies on Promoted Selectivity for CO_2_ Electroreduction to Formate,” *Advanced Materials*, vol. 33, no. 2, p. 2005113, 2021.

[68] Wang, Y.; Huang, Z.; Lei, Y.; et al., “Bismuth with abundant defects for electrocatalytic CO_2_ reduction and Zn–CO_2_ batteries,” *Chemical Communications*, vol. 58, no. 22, pp. 3621–3624, 2022.
